# Supplementary material for: Within-host genetic diversity of extended-spectrum beta-lactamase-producing Enterobacterales in long-term colonized patients
Source: Nat Commun. 2023 Dec 21;14:8495. doi: 10.1038/s41467-023-44285-w (PMC10739949; doi:10.1038/s41467-023-44285-w)
Supplement: Supplementary file 1 — Supplementary Information file [file 41467_2023_44285_MOESM1_ESM.pdf]

Supplementary Information file

Within-host genetic diversity of extended-spectrum beta-lactamase-producing Enterobacterales in long-term colonized patients

AUTHOR LIST

Lisandra Aguilar-Bultet[1,2]; Ana B. García-Martín[1,2]; Isabelle Vock[1,2]; Laura Maurer Pekerman[1,2]; Rahel Stadler[1,2]; Ruth Schindler[1,2]; Manuel Battegay[1,2]; Tanja Stadler[3,4]; Elena Gómez-Sanz[1,2]; Sarah Tschudin-Sutter[1,2*].

AFFILIATIONS

[1] Division of Infectious Diseases and Hospital Epidemiology, University Hospital Basel, University of Basel, Basel, Switzerland

[2] Department of Clinical Research, University Hospital Basel, University of Basel, Basel, Switzerland

[3] Swiss Institute of Bioinformatics, Lausanne, Switzerland

[4] Department of Biosystems Science and Engineering, ETH Zurich, Basel, Switzerland

*Corresponding author:

Prof. Dr. Sarah Tschudin-Sutter

University Hospital Basel

Department of Infectious Diseases & Hospital Epidemiology

Petersgraben 4

CH-4031 Basel

Phone: +41 61 328 45 70

sarah.tschudin@usb.ch

Supplementary figures

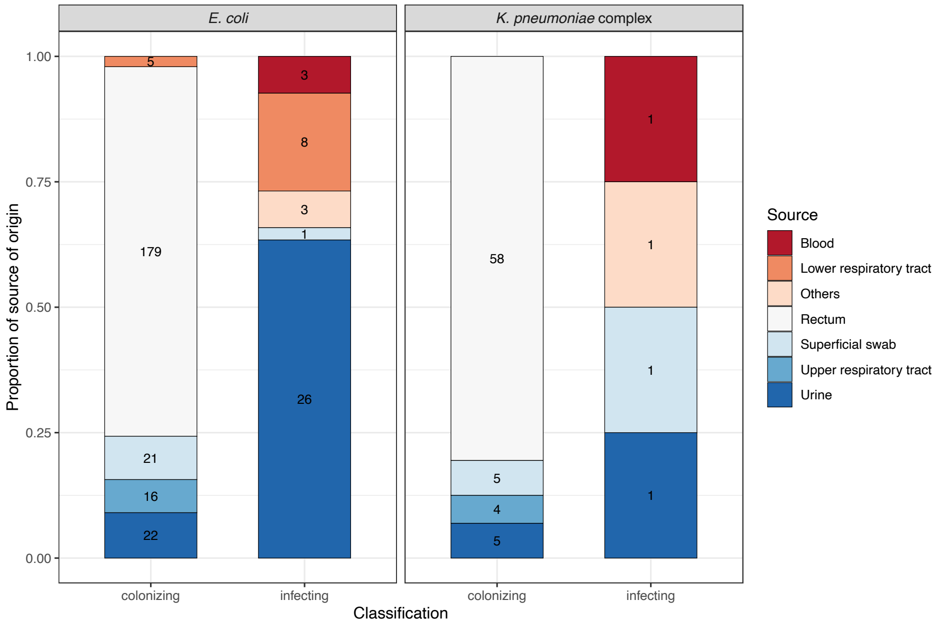

Fig. S1. Distribution of the ESBL-PE isolates stratified by their species (*E. coli* or *K. pneumoniae* species complex) according to their source of origin (different body sites), and classification as colonizing or infecting. Total counts of isolates are indicated inside the stacked bar.

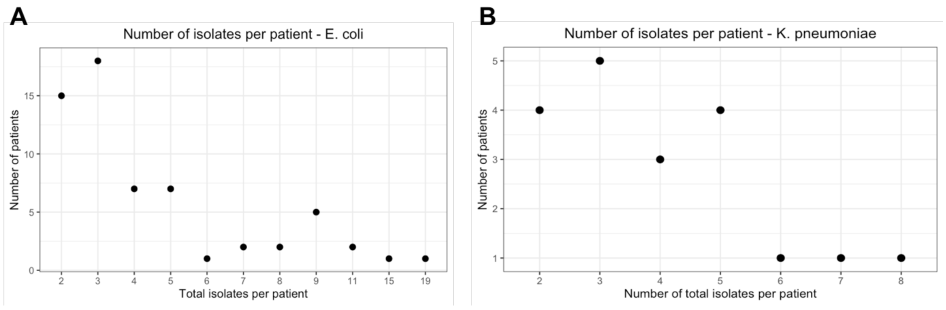

Fig. S2. Total isolates collected per patient in both species. A: *E. coli*. B: *K. pneumoniae* species complex.

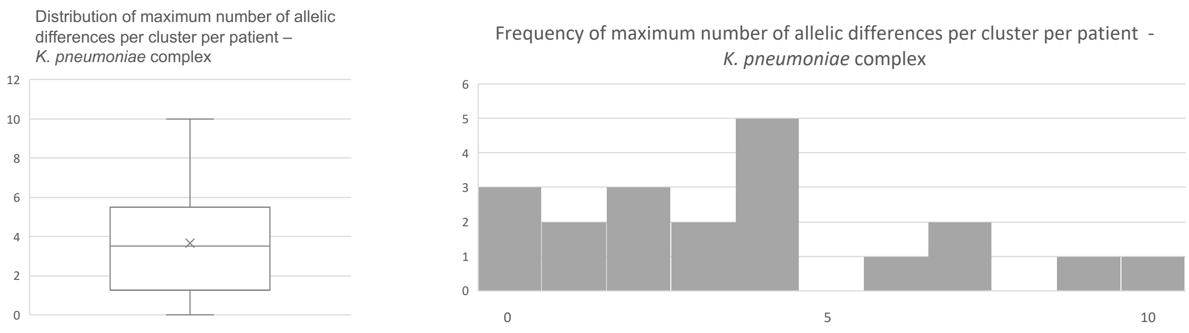

Fig. S3. Distribution and frequency of the number of allelic differences per cluster per patient – *K. pneumoniae* species complex.

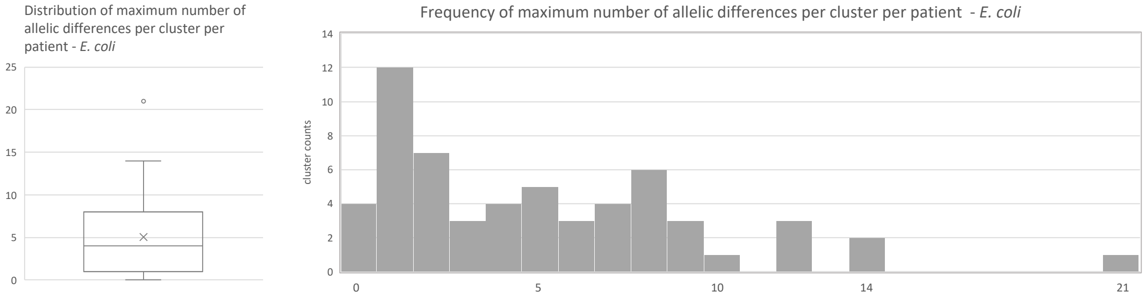

Fig. S4. Distribution and frequency of the number of allelic differences per cluster per patient – *E. coli.*

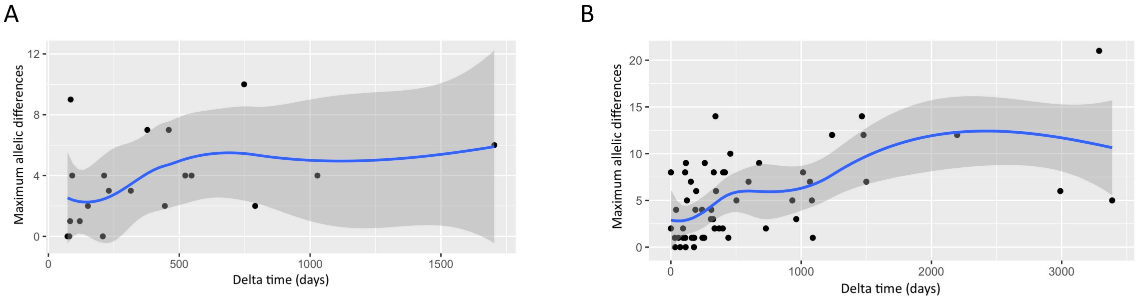

Fig. S5. Maximum allelic differences versus delta time. Each dot represents the maximum number of allelic differences found in this cluster/strain and the maximum delta time. LOESS regression line (Locally Weighted Scatterplot Smoothing) and confidence interval are displayed in blue and grey, respectively. **A.** *K. pneumoniae* species complex. **B:** *E. coli*.

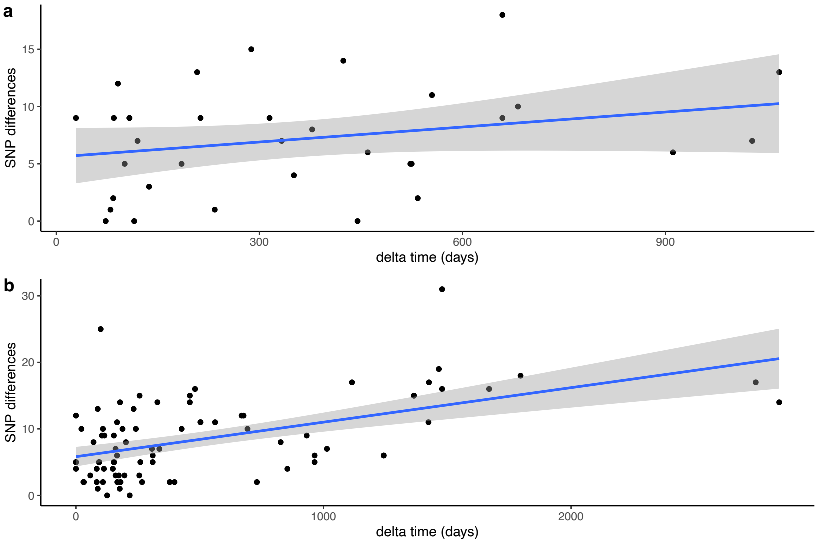

Fig. S6. Overall long-term within-host diversity. Dots represent all SNP differences between the first isolate of the cluster and the rest of the isolates collected subsequently against the delta time between both isolates. *n*-1 (being *n* number of isolates per patient) data points are represented for each patient since the comparison of the first isolate against itself is not included in this analysis. Regression line and confidence interval are displayed in blue and grey, respectively. **A:** *Klebsiella pneumoniae* species complex. **B:** *Escherichia coli*.

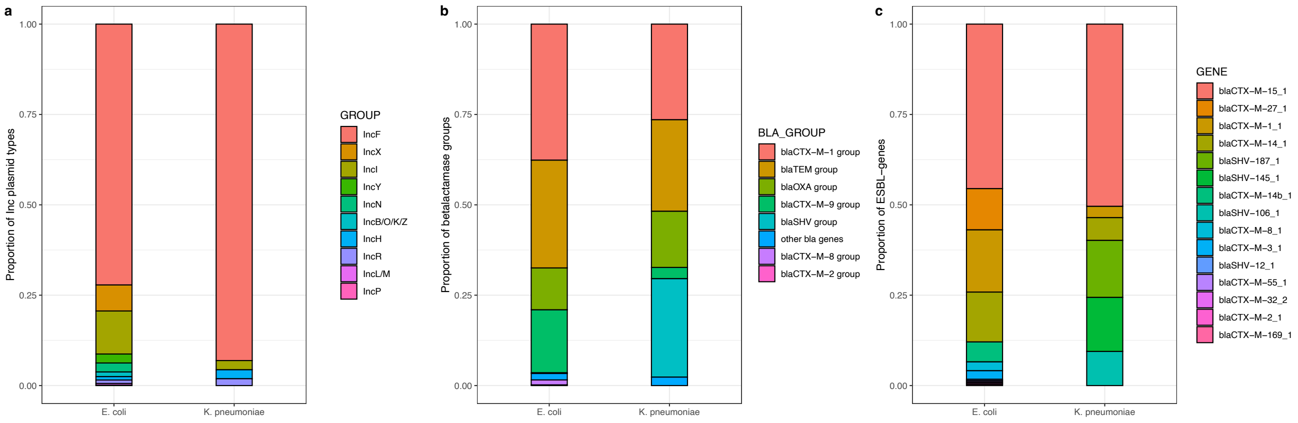

Fig. S7. Distribution of Inc plasmid types and ESBL genes across all isolates included in the study. Hits with < 70% reference-gene coverage and identity were excluded from the analyses. **A:** Proportion of Inc plasmid types per species. **B:** Proportion of beta-lactamase groups per species. **C:** Proportion of ESBL genes per species.

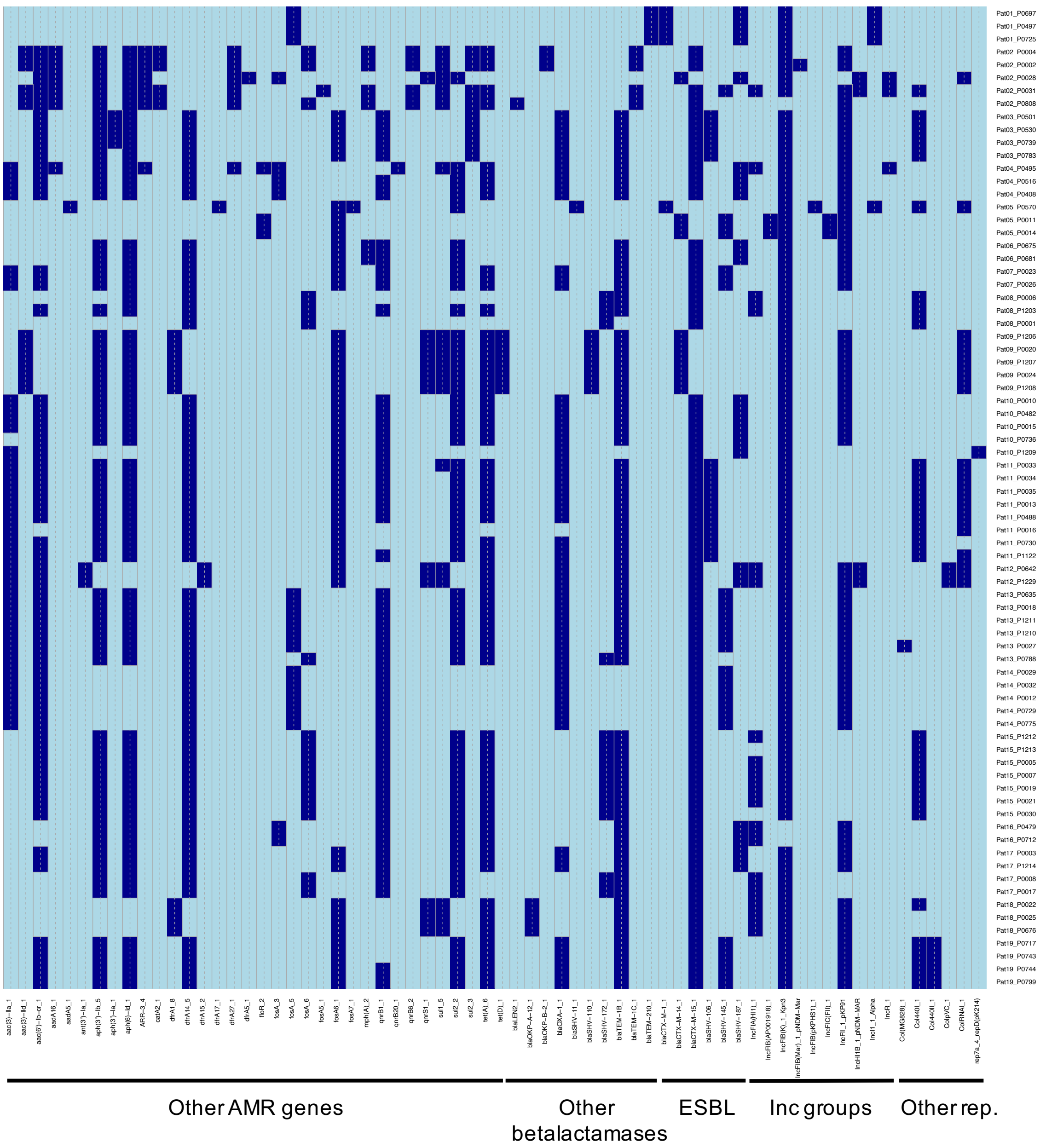

Fig. S8. Heatmap representing the presence/absence of plasmid-associated elements (columns) in isolates of the same host (rows) in *K. pneumoniae* complex. Dark blue: presence of the element; clear blue: absence. Horizontal black lines delimit isolates of the same patient. According to the PacBio sequencing, the genes *oqxA* and *oqxB* are part of the chromosome in the tested isolates and not plasmid elements, hence they were excluded from the analysis. Other

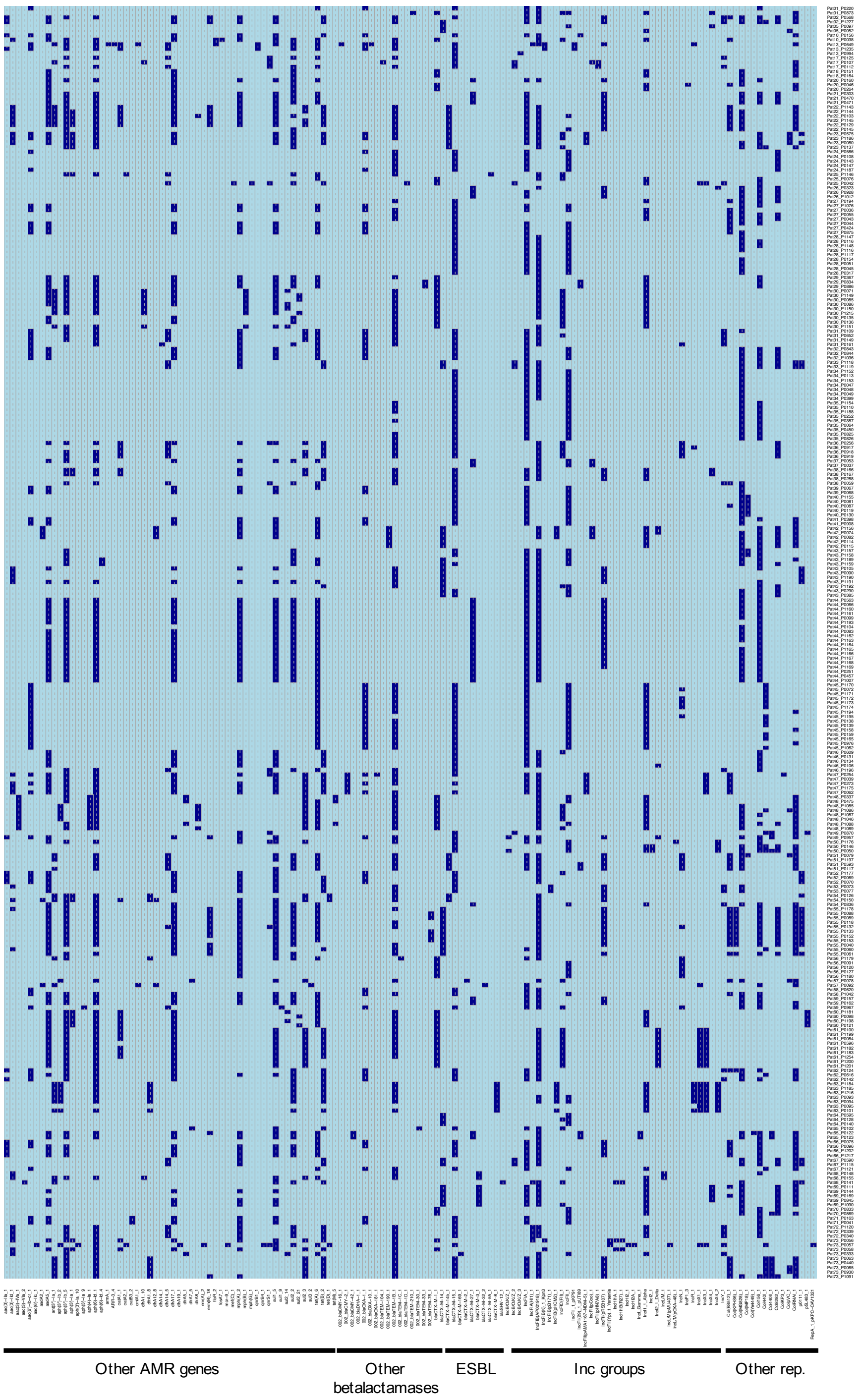

Fig. S9. Heatmap representing the presence/absence of plasmid-associated elements (columns) in isolates of the same host (rows) in *E. coli*. Dark blue: presence of the gene; clear blue: absence. Horizontal black lines delimit isolates of the same patient. According to the PacBio sequencing, *mdf*(A) seems to be part of the chromosome and not plasmid elements.

Supplementary tables

Table S1. Baseline characteristics of patients included in the study, stratified by species. Patients colonized with both species were allocated to each group.

	***Klebsiella pneumoniae* species complex**		***Escherichia coli***	
	**Counts/median**	**Proportions (%)/IQR**	**Counts/median**	**Proportions (%)/IQR**
**Number of patients**	*n*=19		*n*=61	
**Sex (female)**	7	36.8	32	52.5
**Age (years)[a]**	61	IQR 35 – 67	62	IQR 50 – 77.5
**CCI[a]**	2	IQR 1 – 4	1	IQR 0 – 4
**History of hospitalization**	14	73.7	42	52.5
**Previous hospitalization in ICU**	3	15.8	8	10.0
**Travel history**	4	21.1	6	7.5
**Hospitalization abroad**	2	10.5	3	3.8
**History of ESBL-PE colonization**	9	47.4	24	30.0
**History of ESBL-PE infection**	1	5.3	12	15.0
**Permanent urinary catheterization**	0	0.0	6	7.5
**Organ transplantation**	3	15.8	3	3.8
**Allogenic stem cell transplantation**	3	15.8	2	2.5
**Immunosuppressive treatment**	9	47.4	19	23.8
**Proton pump inhibitor treatment**	15	78.4	41	51.3
**Treatment with other antacids**	0	0.0	0	0.0
**Dialysis during hospitalization**	0	0.0	1	1.3

[a] Values shown for age and Charlson Comorbidity Index (CCI) correspond with median and interquartile range (IQR).

Table S2. Maximum number of allelic differences per cluster per patient in *K. pneumoniae* species complex.

**Patient ID**	**Delta time (days)**	**Maximum number of allelic differences**
Pat01	523	4
Pat02	460	7
Pat03	445	2
Pat04	213	4
Pat05	91	4
Pat06	73	0
Pat07	85	9
Pat08	315	3
Pat09	151	2
Pat10	790	2
Pat11	748	10
Pat12	120	1
Pat13	547	4
Pat14	1028	4

Pat15	1704	6
Pat16	378	7
Pat17_cluster1	80	0
Pat17_cluster2	83	1
Pat18	208	0
Pat19	231	3

Table S3. Maximum number of allelic differences per cluster per patient in *E. coli*.

Patient ID	Delta time (days)	Maximum number of allelic differences
Pat20	109	8
Pat21	173	1
Pat22	1067	7
Pat23	239	4
Pat24	1082	5
Pat26	414	8
Pat29	188	4
Pat31	329	8
Pat32	256	1
Pat33	115	9
Pat34	1500	7
Pat35	1466	14
Pat36	342	14
Pat37	71	0
Pat38	260	9
Pat39	58	1
Pat40	441	1
Pat41	177	0
Pat42_cluster1	677	9
Pat42_cluster2	110	1
Pat44	3287	21
Pat45	2989	6
Pat46	324	3
Pat47	194	6
Pat48_cluster1	310	4
Pat48_cluster2	456	10
Pat50	242	1
Pat51	307	3
Pat52	179	1
Pat53	93	2
Pat13	40	4
Pat55	2197	12
Pat56	1089	1
Pat59	32	0
Pat60	596	7

Pat62	398	2
Pat63	346	6
Pat64	962	3
Pat65	30	1
Pat66	252	1
Pat67	729	2
Pat17	337	2
Pat68	113	0
Pat69	1479	12
Pat18	94	1
Pat71	932	5
Pat72	1014	8
Pat73	153	7
Pat27_cluster1	370	2
Pat27_cluster2	160	1
Pat28_cluster1	1237	12
Pat28_cluster2	123	5
Pat30_cluster1	503	5
Pat30_cluster2	0	8
Pat43_cluster1	3387	5
Pat43_cluster2	401	8
Pat61_cluster1	0	2
Pat61_cluster2	335	2

Table S4. ESBL-plasmids obtained by long-read sequencing assemblies, which served as references to map the contigs of the other isolates of the same patient (by Illumina).

**Species**	**Plasmid**	**Size (kb)**	**ESBL gene**	**Inc group**	**Patient**	**Circularized**
K. pneumoniae	P0501_ESBLp	234	*bla*$_{\text{CTX-M-15}}$	IncFIB(K)_Kpn3; IncFII_pKP91; IncQ1	Pat03	yes
K. pneumoniae	P1206_ESBLp	267	*bla*$_{\text{CTX-M-14}}$	IncFIB(K)_Kpn3; IncFII_pKP91	Pat09	yes
K. pneumoniae	P0010_ESBLp	242	*bla*$_{\text{CTX-M-15}}$	IncFIB(K)_Kpn3; IncFII_pKP91	Pat10	yes
K. pneumoniae	P0034_ESBLp	155	*bla*$_{\text{CTX-M-15}}$	IncFIB(K)_Kpn3	Pat11	yes
K. pneumoniae	P0635_ESBLp	223	*bla*$_{\text{CTX-M-15}}$	IncFIB(K)_Kpn3; IncFII_pKP91	Pat13	yes
K. pneumoniae	P0029_ESBLp	165	*bla*$_{\text{CTX-M-15}}$	IncFIB(K)_Kpn3	Pat14	no
K. pneumoniae	P1212_ESBLp	99	*bla*$_{\text{CTX-M-15}}$	IncFIA(HI1)	Pat15	no
K. pneumoniae	P0717_ESBLp	102	*bla*$_{\text{CTX-M-15}}$	IncFII_pKP91	Pat19	yes
E. coli	P1143_ESBLp	139	*bla*$_{\text{CTX-M-14b}}$	IncFIA; IncFIB(AP001918); IncQ1	Pat22	yes
E. coli	P0586_ESBLp	118	*bla*$_{\text{CTX-M-15}}$	IncFIA; IncFII	Pat24	yes
E. coli	P1076_ESBLp	107	*bla*$_{\text{CTX-M-15}}$	IncFIA; IncFII	Pat27	yes
E. coli	P0109_ESBLp	118	*bla*$_{\text{CTX-M-15}}$	IncQ1, IncY	Pat31	yes
E. coli	P0563_ESBLp	135	*bla*$_{\text{CTX-M-159}}$	IncFIA; IncFIB(AP001918); IncFII(pRSB107)	Pat44	yes
E. coli	P0609_ESBLp	136	*bla*$_{\text{CTX-M-15}}$	IncFIA; IncFIB(AP001918); IncFIC(FII)	Pat46	yes
E. coli	P0254_ESBLp	97	*bla*$_{\text{CTX-M-15}}$	IncFIA; IncFIB(AP001918); IncFII(pRSB107); IncQ1	Pat47	no
E. coli	P1085_ESBLp	100	*bla*$_{\text{CTX-M-1}}$	IncI1_Alpha	Pat48	yes
E. coli	P1178_ESBLp	125	*bla*$_{\text{CTX-M-14}}$	IncFIB(AP001918); IncFII(pRSB107)	Pat55	yes
E. coli	P1179_ESBLp	43	*bla*$_{\text{CTX-M-1}}$	IncN	Pat56	yes
E. coli	P0100_ESBLp	59	*bla*$_{\text{CTX-M-1}}$	IncI2_Delta	Pat61	yes
E. coli	P0111_ESBLp_1	86	*bla*$_{\text{CTX-M-3}}$	IncFII	Pat69	no
E. coli	P0111_ESBLp_2	108	*bla*$_{\text{CTX-M-14}}$	IncI-1(Alpha)	Pat69	yes

Table S5. Command line codes used in the data analysis

Tool	Version	Command used
fastp	0.20.0	fastp --in1 ./${PREFIX}_R1.fastq.gz --in2 ./${PREFIX}_R2.fastq.gz -c -p --adapter_fasta Nextera_adapt.fasta --out1 ./${PREFIX}_R1.ftd.fq.gz --out2 ./${PREFIX}_R2.ftd.fq.gz -h "${PREFIX}.html" -j "${PREFIX}.json"
kraken2	2.0.8	kraken2 --use-names -db kraken2_db --threads 8 --gzip-compressed --report ${PREFX}_kraken2.report --paired ./${PREFIX}_R1.ftd.fq.gz ./${PREFIX}_R2.ftd.fq.gz > ${PREFIX}_kraken2.info
shovill	1.0.9	shovill --R1 ${PREFIX}_R1.ftd.fq.gz --R2 ${PREFIX}\_R2.ftd.fq.gz --gsize 5M --outdir shovill_${PREFIX} --minlen 500 --tmpdir $TMPDIR/shovill_inhost --cpus 8 --ram 16 --assembler spades
flye	2.6	flye --pacbio-raw $sample --genome-size 5m --out-dir ${sample}_flye_contigs --threads 8 --iterations 3 --plasmids
flye	2.9.2	flye --pacbio-hifi $sample --genome-size 5m --out-dir ${sample}_flye_contigs --threads 8 --iterations 3 --plasmids
unicycler	4.6	unicycler -1 ${PREFIX}_R1.ftq.fq.gz -2 ${PREFIX}_R2.ftq.fq.gz -l $sample -o ${PREFIX}_unicycler --mode normal --verbosity 2 --min_fasta_length 500 --keep 2 --bcftools_path /scicore/soft/apps/BCFtools/1.9-fo ss-2018b/bin/bcftools --vcf -t 8 --min_polish_size 500 --min_component_size 500 --min_dead_end_size 500
prokka	1.12	prokka --outdir ${PREFIX2} --force --kingdom Bacteria --gram neg --locustag ${PREFIX2} --genus $1 --species $2 --strain ${PREFIX2} --usegenus --rfam --mincontiglen 500 ${PREFIX1}
snippy	4.6.0	snippy --cpus ${SLURM_CPUS_PER_TASK} --outdir ${line}_snippy --ref ../${REFERENCE} --R1 ${line}_R1.ftd.fq.gz --R2 ${line}_R2.ftd.fq.gz
snippy	4.6.0	snippy-core --prefix core --ref ../${REFERENCE} ${CORE_LIST}
abricate	0.8.7	abricate --threads 8 --datadir ~/abricate_dbs/ -db plasmidfinder $g > ${PREFIX1}.plasmidfinder
abricate	0.8.7	abricate --threads 8 --datadir ~/abricate_dbs/ -db resfinder $g > ${PREFIX1}.resfinder
kleborate	2.3.2	kleborate-runner.py --all -o ${g}_all_kleborate.txt -a ${g}

Table S6. Antimicrobial resistance genes detected only in the chromosome of the isolates sequenced with PacBio (*Escherichia coli* n= 98, *Klebsiella pneumoniae* species complex n=26).

Species	AMR gene	Number of isolates with gene in the chromosome	Frequency
E. coli	*blaCMY-2_1*	2	2
E. coli	*blaTEM-190_1*	3	3.1
E. coli	*mdf(A)_1*	98	100
E. coli	*tet(M)_5*	2	2
K. pneumoniae species complex	*blaLEN2_1*	1	3.8
K. pneumoniae species complex	*blaOKP-B-2_1*	2	7.7
K. pneumoniae species complex	*blaSHV-106_1*	2	7.7
K. pneumoniae species complex	*blaSHV-110_1*	2	7.7
K. pneumoniae species complex	*blaSHV-172_1*	4	15.4
K. pneumoniae species complex	*blaSHV-187_1*	7	26.9
K. pneumoniae species complex	*blaSHV-194_1*	2	7.7
K. pneumoniae species complex	*blaSHV-26_1*	4	15.4
K. pneumoniae species complex	*blaSHV-28_1*	2	7.7
K. pneumoniae species complex	*fosA_3*	2	7.7
K. pneumoniae species complex	*fosA_5*	5	19.2
K. pneumoniae species complex	*fosA_6*	7	26.9
K. pneumoniae species complex	*fosA5_1*	1	3.8
K. pneumoniae species complex	*fosA6_1*	11	42.3
K. pneumoniae species complex	*oqxA*	26	100
K. pneumoniae species complex	*oqxB*	26	100
