# Supplementary material for: Within-host genetic diversity of extended-spectrum beta-lactamase-producing Enterobacterales in long-term colonized patients
Source: Nat Commun. 2023 Dec 21;14:8495. doi: 10.1038/s41467-023-44285-w (PMC10739949; doi:10.1038/s41467-023-44285-w)
Supplement: Supplementary file 3 — Description of Additional Supplementary Files [file 41467_2023_44285_MOESM3_ESM.pdf]

Description of Additional Supplementary Data files

File Name: Supplementary Data S1

Description:

- List of *Klebsiella pneumoniae* species complex isolates used for analysis with metadata (Patient ID, cluster per patient - CPP, sample date, origin, classification in colonizing or infecting isolate, species verification by Kraken2, sequence type - ST, Complex type, MLST profile, cgMLST cluster).
- *K. pneumoniae* isolates present at the same time period (up to 3 months), with delta time per patient, information about being same cgMLST cluster or not and how many allelic differences.
- List of *Escherichia coli* isolates used for analysis with metadata (Patient ID, cluster per patient - CPP, sample date, origin, classification in colonizing or infecting isolate, sequence type – ST by Warwick system, Complex type, MLST profile, cgMLST cluster).
- *E. coli* isolates present at the same time period (up to 3 months), with delta time per patient, information about being same cgMLST cluster or not and how many allelic differences.
- All NCBI accession numbers for Bioproject, Biosample and SRA (Illumina and PacBio).

File Name: Supplementary Data S2

Description:

- AMR and replicon genes identified in *K. pneumoniae* species complex isolates, with their Class, group assignation, percentage of coverage and identity, accession numbers and putative phenotype (resistance).
- For each gene detected in *K. pneumoniae* species complex, we calculated the number of patients (and percentage) with this gene, the median number of isolates (in percent) of this patient carrying the gene, and the total number of isolates in the dataset carrying the gene. Median gives an idea of the persistence of the gene within the same patient.
- AMR and replicon genes identified in *E. coli* isolates, with their Class, group assignation, percentage of coverage and identity, accession numbers and putative phenotype (resistance).
- For each gene detected in *E. coli*, we calculated the number of patients (and percentage) with this gene, the median number of isolates (in percent) of this patient carrying the gene, and the total number of isolates in the dataset carrying the gene. Median gives an idea of the persistence of the gene within the same patient.
- Patients with more than one species, and their isolates (with metadata).
- AMR and replicon genes in patients with multiple species. ESBL genes and Incompatibility types shares between isolates of different species.

File Name: Supplementary Data S3

Description: Kleborate results.

File Name: Supplementary Data S4

Description:

- *E. coli* patients (and classification in short/long colonizers) and their baseline characteristics. Fisher test results.
- *K. pneumoniae* species complex patients (and classification in short/long colonizers) and their baseline characteristics. Fisher test results.

File Name: Supplementary Data S5

Description:

- Most common ESBL genes and Incompatibility (Inc) types detected in *E. coli* strains. Fisher test results.
- Most common ESBL genes and Incompatibility (Inc) types detected in *K. pneumoniae* species complex strains. Fisher test results.

File Name: Supplementary Data S6

Description:

- Persistence of whole ESBL-plasmids within the same patient in *E. coli* isolates at different levels (isolates of the same strain – biggest clusters or longest clusters, and isolates of different strains).
- Persistence of whole ESBL-plasmids within the same patient in *K. pneumoniae* species complex isolates at different levels (isolates of the same strain – biggest clusters or longest clusters, and isolates of different strains).
- Presence of the same ESBL-plasmid in isolates of different species within the same patient.
