# Supplementary material for: Within-host genetic diversity of extended-spectrum beta-lactamase-producing Enterobacterales in long-term colonized patients
Source: Nat Commun. 2023 Dec 21;14:8495. doi: 10.1038/s41467-023-44285-w (PMC10739949; doi:10.1038/s41467-023-44285-w)
Supplement: Supplementary file 10 — Reporting Summary [file 41467_2023_44285_MOESM10_ESM.pdf]

nature portfolio

Corresponding author(s): Sarah Tschudin Sutter

Last updated by author(s): Nov 10, 2023

Reporting Summary

Nature Portfolio wishes to improve the reproducibility of the work that we publish. This form provides structure for consistency and transparency in reporting. For further information on Nature Portfolio policies, see our Editorial Policies and the Editorial Policy Checklist.

Statistics

For all statistical analyses, confirm that the following items are present in the figure legend, table legend, main text, or Methods section.

n/a	Confirmed	
☐	☒	The exact sample size (*n*) for each experimental group/condition, given as a discrete number and unit of measurement
☐	☒	A statement on whether measurements were taken from distinct samples or whether the same sample was measured repeatedly
☐	☒	The statistical test(s) used AND whether they are one- or two-sided<br>*Only common tests should be described solely by name; describe more complex techniques in the Methods section.*
☐	☒	A description of all covariates tested
☐	☒	A description of any assumptions or corrections, such as tests of normality and adjustment for multiple comparisons
☐	☒	A full description of the statistical parameters including central tendency (e.g. means) or other basic estimates (e.g. regression coefficient) AND variation (e.g. standard deviation) or associated estimates of uncertainty (e.g. confidence intervals)
☐	☒	For null hypothesis testing, the test statistic (e.g. *F*, *t*, *r*) with confidence intervals, effect sizes, degrees of freedom and *P* value noted<br>*Give P values as exact values whenever suitable.*
☒	☐	For Bayesian analysis, information on the choice of priors and Markov chain Monte Carlo settings
☒	☐	For hierarchical and complex designs, identification of the appropriate level for tests and full reporting of outcomes
☒	☐	Estimates of effect sizes (e.g. Cohen's *d*, Pearson's *r*), indicating how they were calculated

Our web collection on statistics for biologists contains articles on many of the points above.

Software and code

Policy information about availability of computer code

Data collection

Patient data collection was performed in RedCap.

Data analysis

For data analysis, we used the following programs and versions:
fastp v.0.20.0
kraken2 v.2.0.8
shovill v.1.0.9
flye v.2.6 and v.2.9
unicycler v.4.6
prokka v.1.12
Ridom SeqSphere+ v.6.0
snippy v.4.6.0
ClonalFrameML v.1.12
snp-dist v.0.8.2
abricate v.1.0.1
kleborate v.2.3.2
All details of the commands used are found in the Supplementary Information file.

Databases accessed for data analysis:
Resfinder (accessed on July 1st 2021)
Plasmidfinder (accessed on July 1st 2021)

For statistical analysis we used R v. 4.2.1.

For manuscripts utilizing custom algorithms or software that are central to the research but not yet described in published literature, software must be made available to editors and reviewers. We strongly encourage code deposition in a community repository (e.g. GitHub). See the Nature Portfolio guidelines for submitting code & software for further information.

Data

Policy information about availability of data

All manuscripts must include a data availability statement. This statement should provide the following information, where applicable:

- Accession codes, unique identifiers, or web links for publicly available datasets
- A description of any restrictions on data availability
- For clinical datasets or third party data, please ensure that the statement adheres to our policy

All sequencing and sample data from this study can be accessed at the NCBI database under the BioProject number PRJNA910977. The Source Date file can be accessed at: https://doi.org/10.5281/zenodo.10116672.

Research involving human participants, their data, or biological material

Policy information about studies with human participants or human data. See also policy information about sex, gender (identity/presentation), and sexual orientation and race, ethnicity and racism.

Reporting on sex and gender	Sex (biological attribute) was indicated in the Supplementary Table S1.
Reporting on race, ethnicity, or other socially relevant groupings	We did not use any socially constructed or socially relevant categorization variable(s) in our manuscript.
Population characteristics	Pertinent clinical and microbiological data were collected retrospectively from electronic medical records and entered into a secured REDCap database. Assessed variables were (1) demographics (2) previous hospitalizations (defined as any hospitalization of at least two days within the past 12 months prior to index hospitalization), (3) travel history (defined as a stay outside of Switzerland within 12 months prior to the index hospitalization), hospitalization abroad (defined as hospitalization outside of Switzerland within 12 months prior to the index hospitalization), (4) comorbidities based on the Charlson Comorbidity Index (CCI), (5) receipt of dialysis during hospitalization, (6) history of organ or (7) allogenic stem cell transplantation, (8) permanent urinary catheterization, (9) proton-pump inhibitor (PPI) and/or other antacid usage concomitant medication, immunosuppressive medication within 12 months prior to index sample, and a known (10) history of ESBL colonization and/or infection within the previous 12 months.<br>The Supplementary Table S1 summarizes the baseline characteristics of all the patients included in this study, stratified by species.
Recruitment	This observational cohort study was performed at the University Hospital Basel, a 735-bed tertiary care center in Basel (Switzerland), which admits approximately 35'000 adult patients annually. Since 2003, all patients with recovery of ESBL-PE from any specimen obtained by routine clinical practice in both in and outpatient settings are routinely screened to determine further colonization sites. Screening for ESBL-PE carriage is performed by selective plating of rectal swabs, swabs from open wounds or drainages, as well as urine samples from patients with urinary catheters. Patients admitted to the University Hospital Basel from 01/2008 to 12/2018 with detection of ESBL-PE isolates belonging to the same species (K. pneumoniae or E. coli) in at least two consecutive rectal swabs were included in this study. Patients with rejection of the general informed consent were excluded. As participation was not based upon voluntary participation, we rule out the potential for self-selection bias. As recovery of ESBL-PE in at least two consecutive rectal swabs was defined as an inclusion criterium of our study and rectal swabs are systematically performed at hospital admission, the study population may be biased towards a "sicker" patient population requiring re-admission. We added the last consideration to the limitation section of our manuscript.
Ethics oversight	This study adhered to the Strengthening the Reporting of Observational Studies in Epidemiology (STROBE) guidelines for reporting of observational studies. It was approved by the local ethics committee (EKNZ-2017 00100) and it is part of the registered NRP project "Transmission of ESBL-producing Enterobacteriaceae" (ClinicalTrials.gov Identifier: NCT03465683).

Note that full information on the approval of the study protocol must also be provided in the manuscript.

Field-specific reporting

Please select the one below that is the best fit for your research. If you are not sure, read the appropriate sections before making your selection.

☒ Life sciences ☐ Behavioural & social sciences ☐ Ecological, evolutionary & environmental sciences

For a reference copy of the document with all sections, see nature.com/documents/nr-reporting-summary-flat.pdf

Life sciences study design

All studies must disclose on these points even when the disclosure is negative.

Sample size	No sample size calculation was applied. Our study involves 73 consecutive patients and 360 bacterial isolates. Patients admitted to the University Hospital Basel from 01/2008 to 12/2018 with detection of ESBL-PE isolates belonging to the same species (K. pneumoniae or E. coli) in at least two consecutive rectal swabs were included in this study. Colonizing and/or infecting isolates collected from different body sites from patients meeting these inclusion criteria were included. Thus, the sample size was choosen based on the number of patients presenting to our institution during the defined time-period. As our study is exploratory and not designed to prove or refute the effect of any intervention or difference between two groups, we consider our sample size appropriate to derive some meaningful insights into the research question addressed.
Data exclusions	Patients with rejection of the general informed consent were excluded.
Replication	This is an observational retrospective study and hence does not involve any experiment in nature. Thus, no replication of our analyses were performed. Replication of the sequencing runs was not performed since this is not a common practice, and well established sequencing protocols and software for data analysis were used.
Randomization	This is not a randomized controlled clinical trial and no experimental groups were involved. Thus, no randomization was performed. As no allocation to two different intervention groups was performed and the study did not aim to demonstrate any effect or risk factor, no controlling for potential confounders was required.
Blinding	This is not a randomized controlled clinical trial and no experimental groups were involved. Thus, no blinding was performed.

Reporting for specific materials, systems and methods

We require information from authors about some types of materials, experimental systems and methods used in many studies. Here, indicate whether each material, system or method listed is relevant to your study. If you are not sure if a list item applies to your research, read the appropriate section before selecting a response.

Materials & experimental systems

n/a	Involved in the study	
☒	☐	Antibodies
☒	☐	Eukaryotic cell lines
☒	☐	Palaeontology and archaeology
☒	☐	Animals and other organisms
☐	☒	Clinical data
☒	☐	Dual use research of concern
☒	☐	Plants

Methods

n/a	Involved in the study	
☒	☐	ChIP-seq
☒	☐	Flow cytometry
☒	☐	MRI-based neuroimaging

Clinical data

Policy information about clinical studies

All manuscripts should comply with the ICMJE guidelines for publication of clinical research and a completed CONSORT checklist must be included with all submissions.

Clinical trial registration	ClinicalTrials.gov Identifier: NCT03465683
Study protocol	The study protocol was submitted to the local ethics committee (EKNZ-2017 00100). The study protocol was not published as this is not a clinical trial. Furthermore the study represents a sub-project of a larger study, the study protocol of which has been published and is cited in the manuscript (Ref. 24)
Data collection	This observational cohort study was performed at the University Hospital Basel, a 735-bed tertiary care center in Basel (Switzerland), which admits approximately 35’000 adult patients annually. Since 2003, all patients with recovery of ESBL-PE from any specimen obtained by routine clinical practice in both in and outpatient settings are routinely screened to determine further colonization sites. Screening for ESBL-PE carriage is performed by selective plating of rectal swabs, swabs from open wounds or drainages, as well as urine samples from patients with urinary catheters. Patients admitted to the University Hospital Basel from 01/2008 to 12/2018 with detection of ESBL-PE isolates belonging to the same species (K. pneumoniae or E. coli) in at least two consecutive rectal swabs were included in this study. Patients with rejection of the general informed consent were excluded.
Outcomes	The outcomes of this study are: (1) estimates of the diversity of ESBL-producing E. coli and/or K. pneumoniae within the same host at a given time point and over time, (2) estimates of the potential duration of colonization with the same strain and the same plasmids and ESBL genes, (3) estimates of the potential for plasmid and gene transmission events between different strains/species recovered from the same patient, (4) insights into the number of single-nucleotide polymorphisms (SNPs) accumulated over time in isolates belonging to the same cluster within the same host. The outcomes were assessed by applying the specific approaches outlined in the methods sections. We added the following statement to the manuscript: "The outcomes were assessed by applying the specific

approaches outlined in the methods sections assessment of genetic relatedness and diversity, assessment of plasmid diversity and persistence and statistical analyses."

Plants

Seed stocks

Report on the source of all seed stocks or other plant material used. If applicable, state the seed stock centre and catalogue number. If plant specimens were collected from the field, describe the collection location, date and sampling procedures.

Novel plant genotypes

Describe the methods by which all novel plant genotypes were produced. This includes those generated by transgenic approaches, gene editing, chemical/radiation-based mutagenesis and hybridization. For transgenic lines, describe the transformation method, the number of independent lines analyzed and the generation upon which experiments were performed. For gene-edited lines, describe the editor used, the endogenous sequence targeted for editing, the targeting guide RNA sequence (if applicable) and how the editor was applied.

Authentication

Describe any authentication procedures for each seed stock used or novel genotype generated. Describe any experiments used to assess the effect of a mutation and, where applicable, how potential secondary effects (e.g. second site T-DNA insertions, mosiacism, off-target gene editing) were examined.
