# Supplementary figures and images for: Within-host genetic diversity of extended-spectrum beta-lactamase-producing Enterobacterales in long-term colonized patients
Source: Nat Commun. 2023 Dec 21;14:8495. doi: 10.1038/s41467-023-44285-w (PMC10739949; doi:10.1038/s41467-023-44285-w)

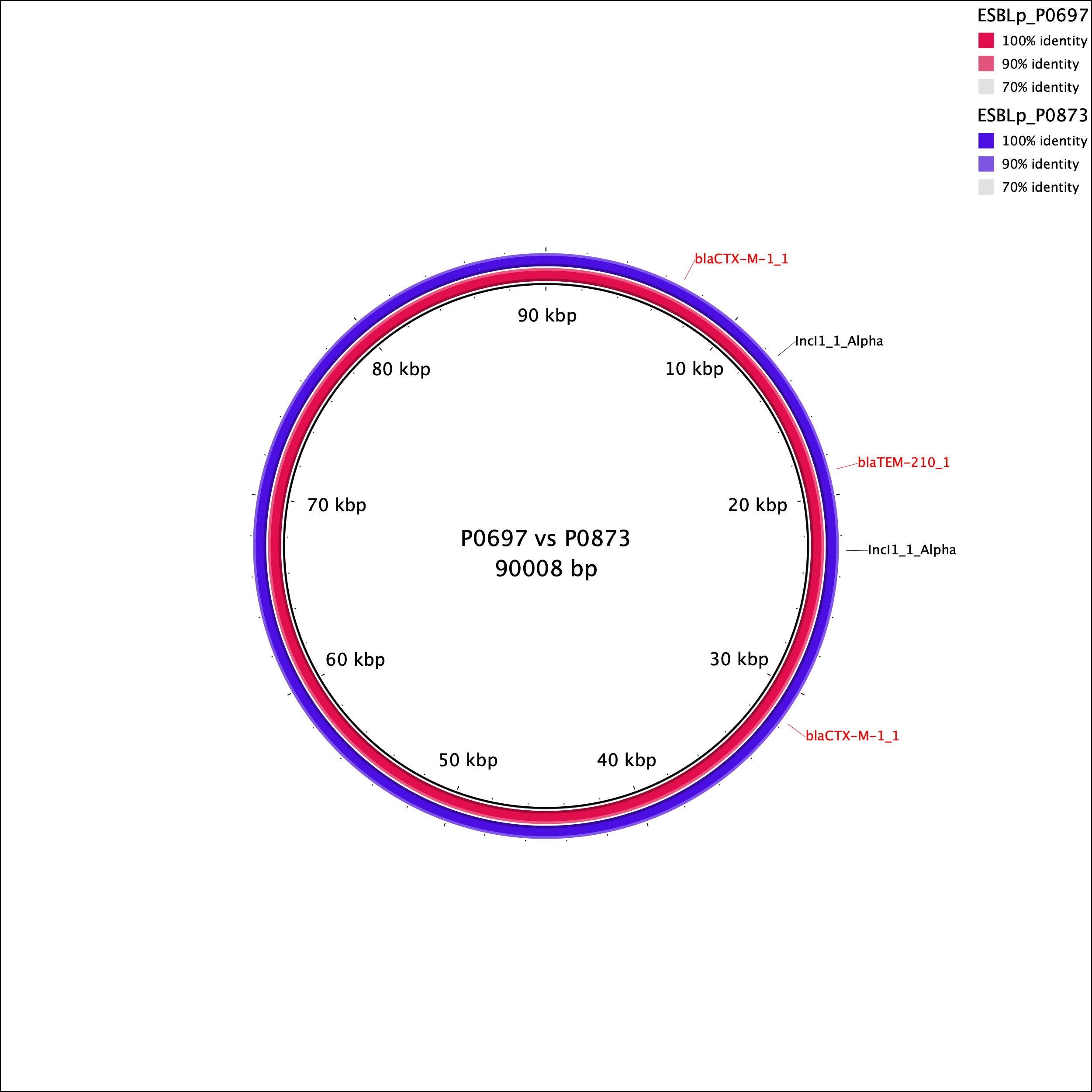

Supplement: Supplementary file 11 — Source Data [file 41467_2023_44285_MOESM11_ESM.zip › SourceDataFile/ESBLp_figures/bothSpp_ESBLp_BRIG_figures_allPacBio/P0697_ESBLp.fasta.jpg]

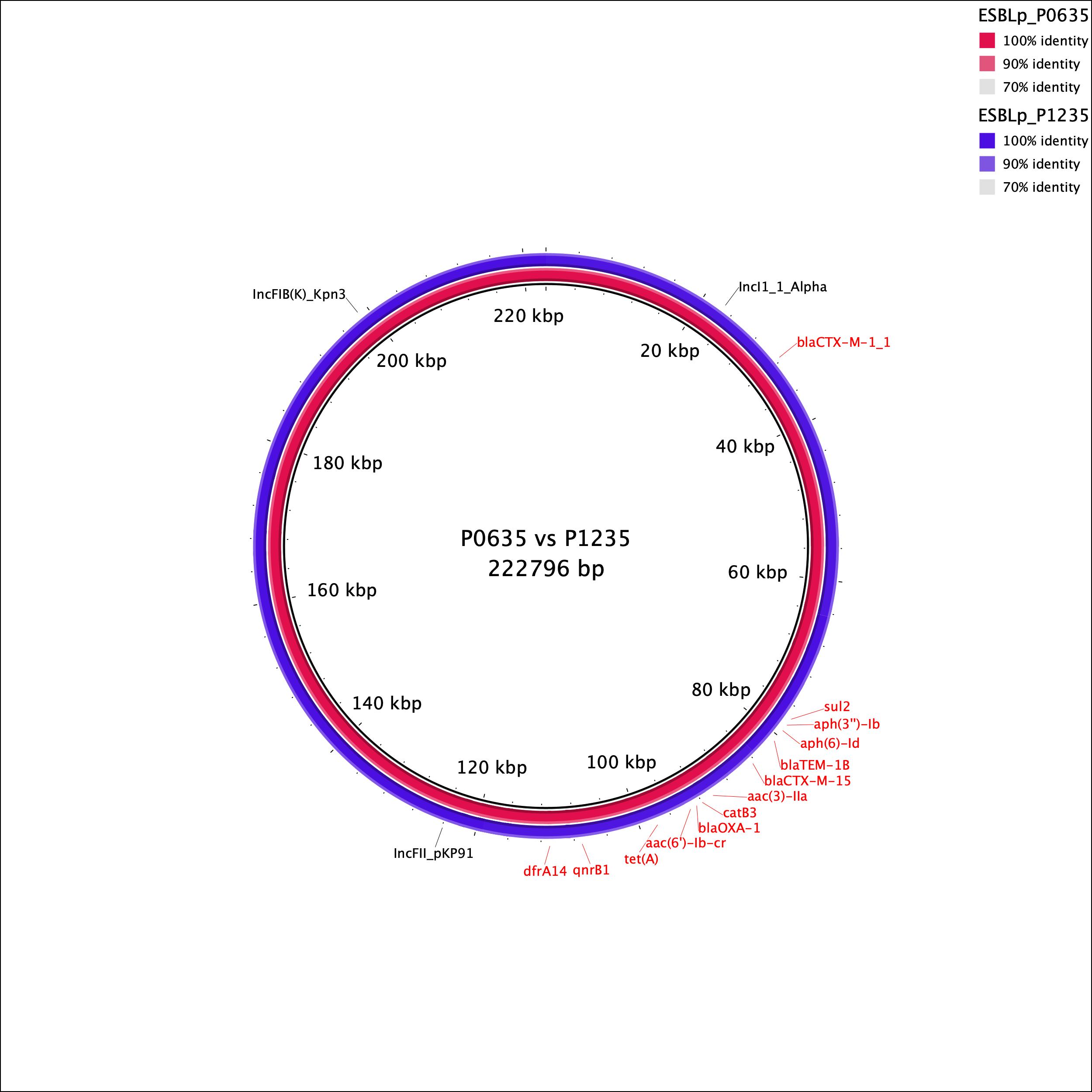

Supplement: Supplementary file 11 — Source Data [file 41467_2023_44285_MOESM11_ESM.zip › SourceDataFile/ESBLp_figures/bothSpp_ESBLp_BRIG_figures_allPacBio/P0635_ESBLp.fasta.jpg]

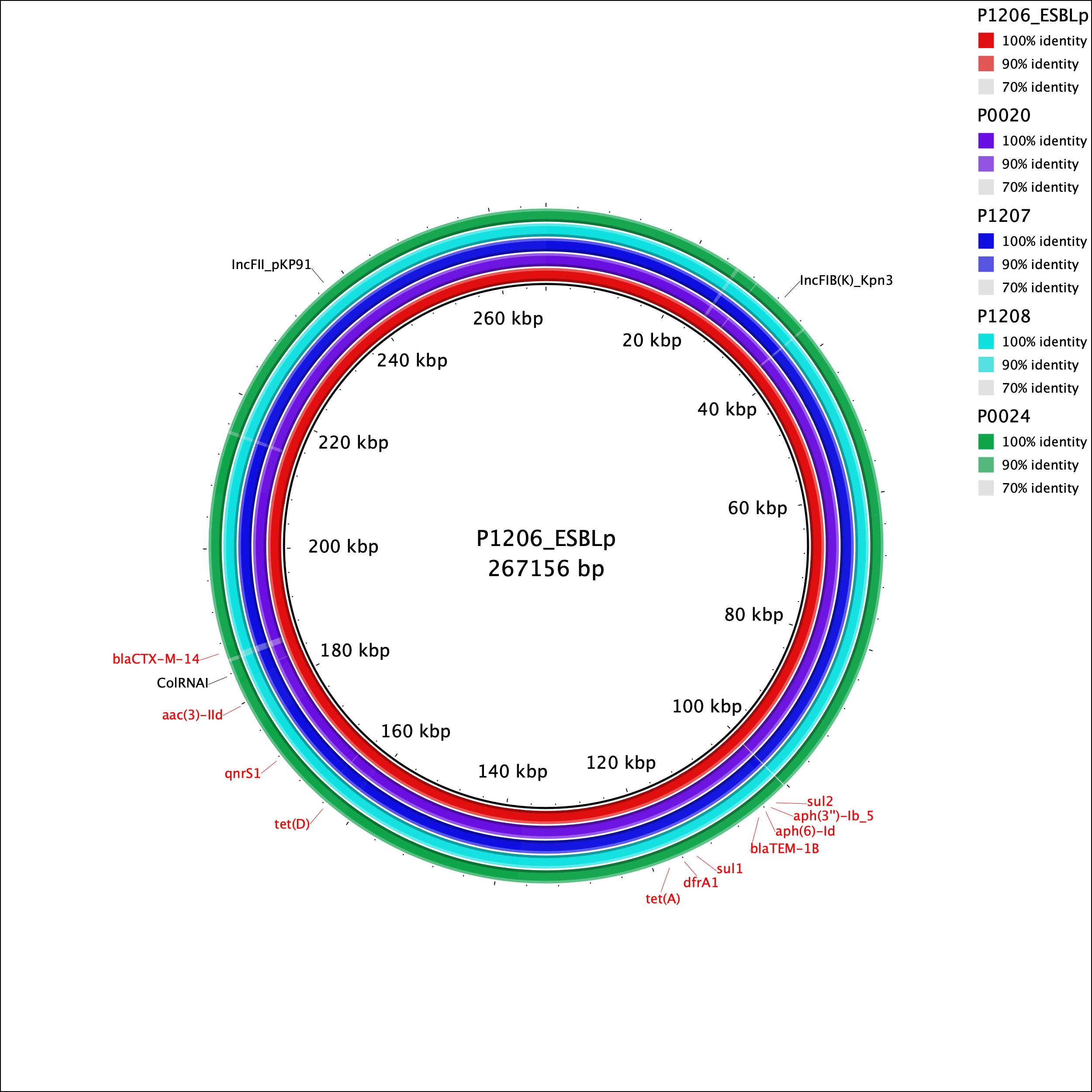

Supplement: Supplementary file 11 — Source Data [file 41467_2023_44285_MOESM11_ESM.zip › SourceDataFile/ESBLp_figures/Kpneumoniae_BRIG_figures_refPacBio_othersIllumina/P1206_ESBLp.jpg]

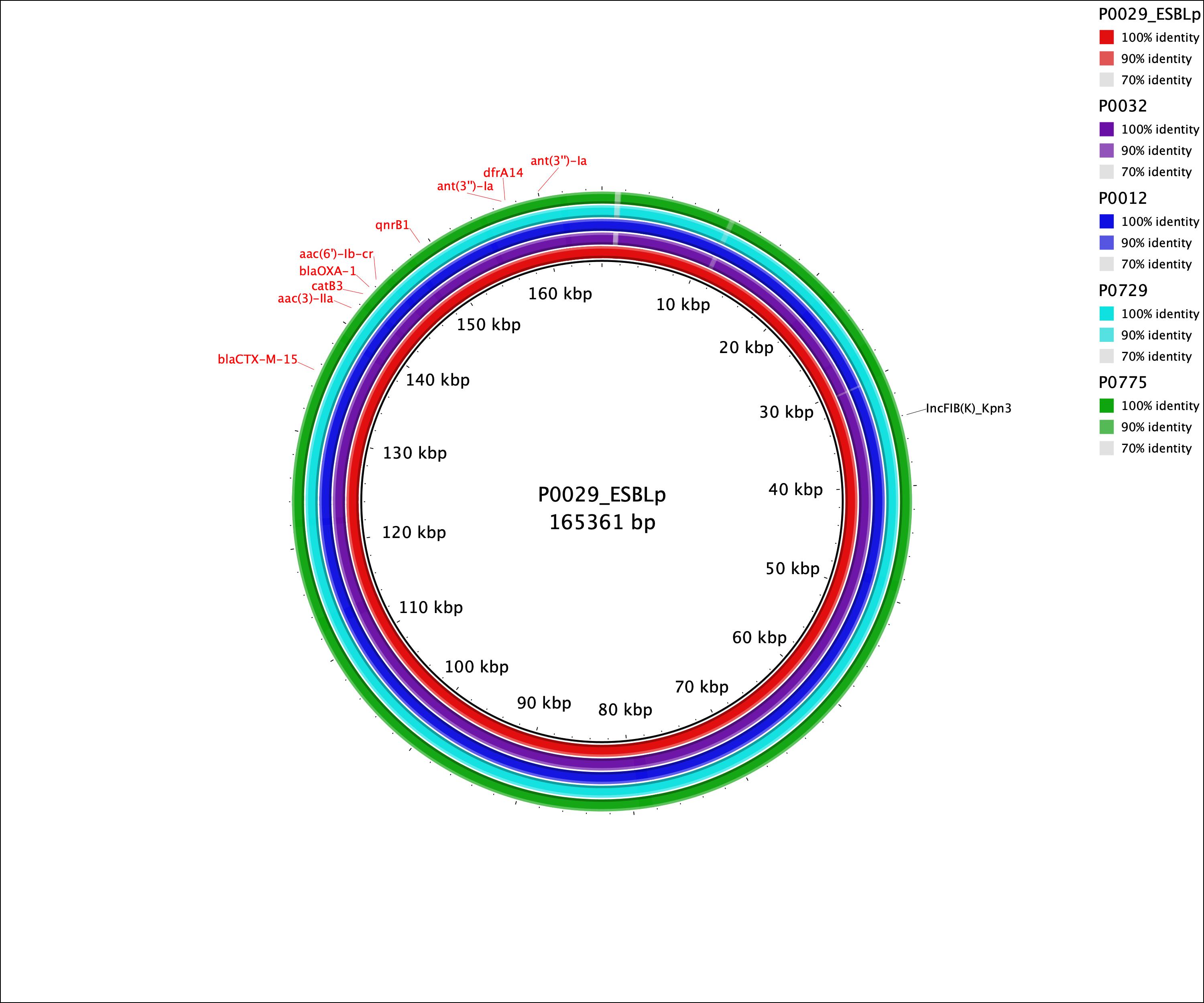

Supplement: Supplementary file 11 — Source Data [file 41467_2023_44285_MOESM11_ESM.zip › SourceDataFile/ESBLp_figures/Kpneumoniae_BRIG_figures_refPacBio_othersIllumina/P0029_ESBLp.fasta.jpg]

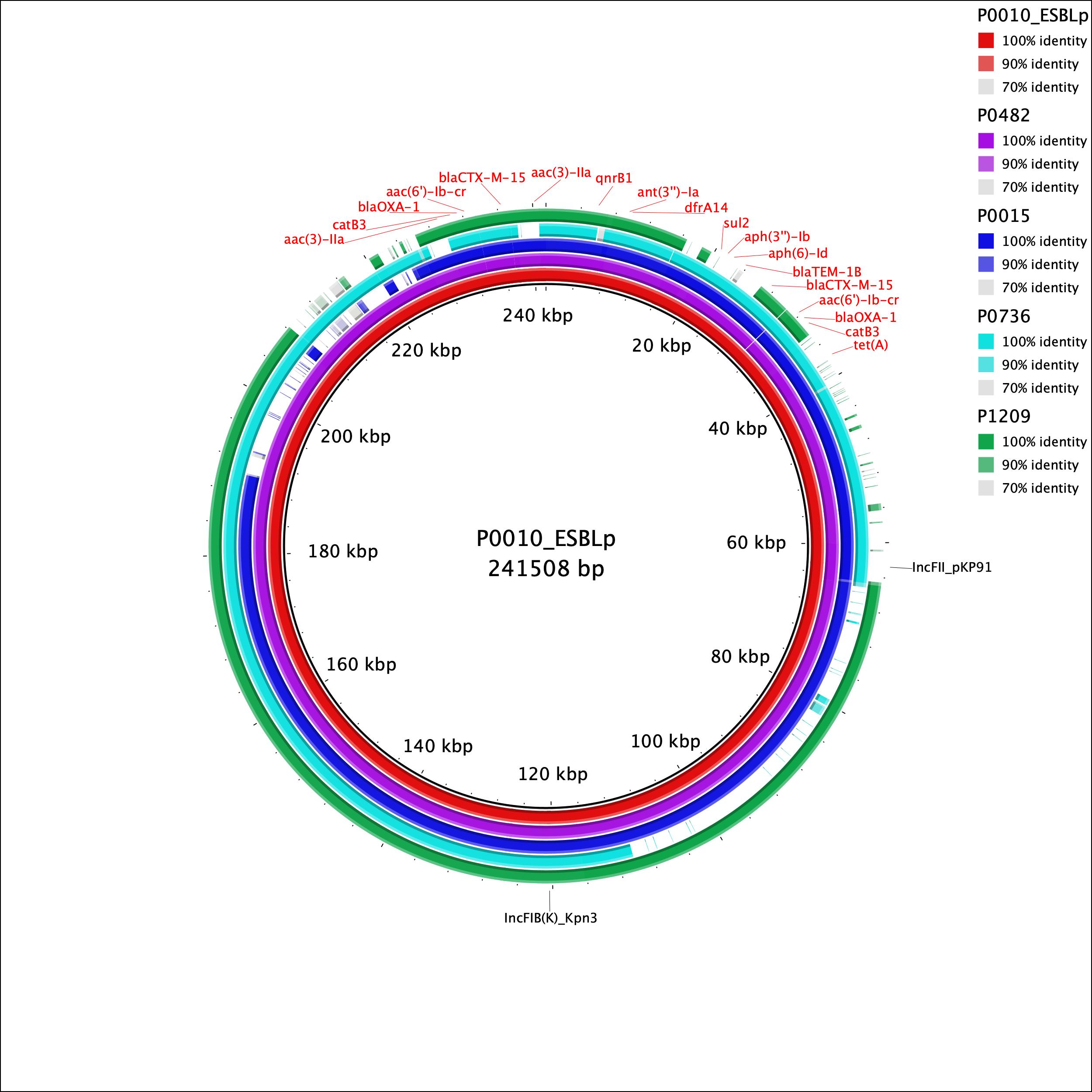

Supplement: Supplementary file 11 — Source Data [file 41467_2023_44285_MOESM11_ESM.zip › SourceDataFile/ESBLp_figures/Kpneumoniae_BRIG_figures_refPacBio_othersIllumina/P0010_ESBLp.jpg]

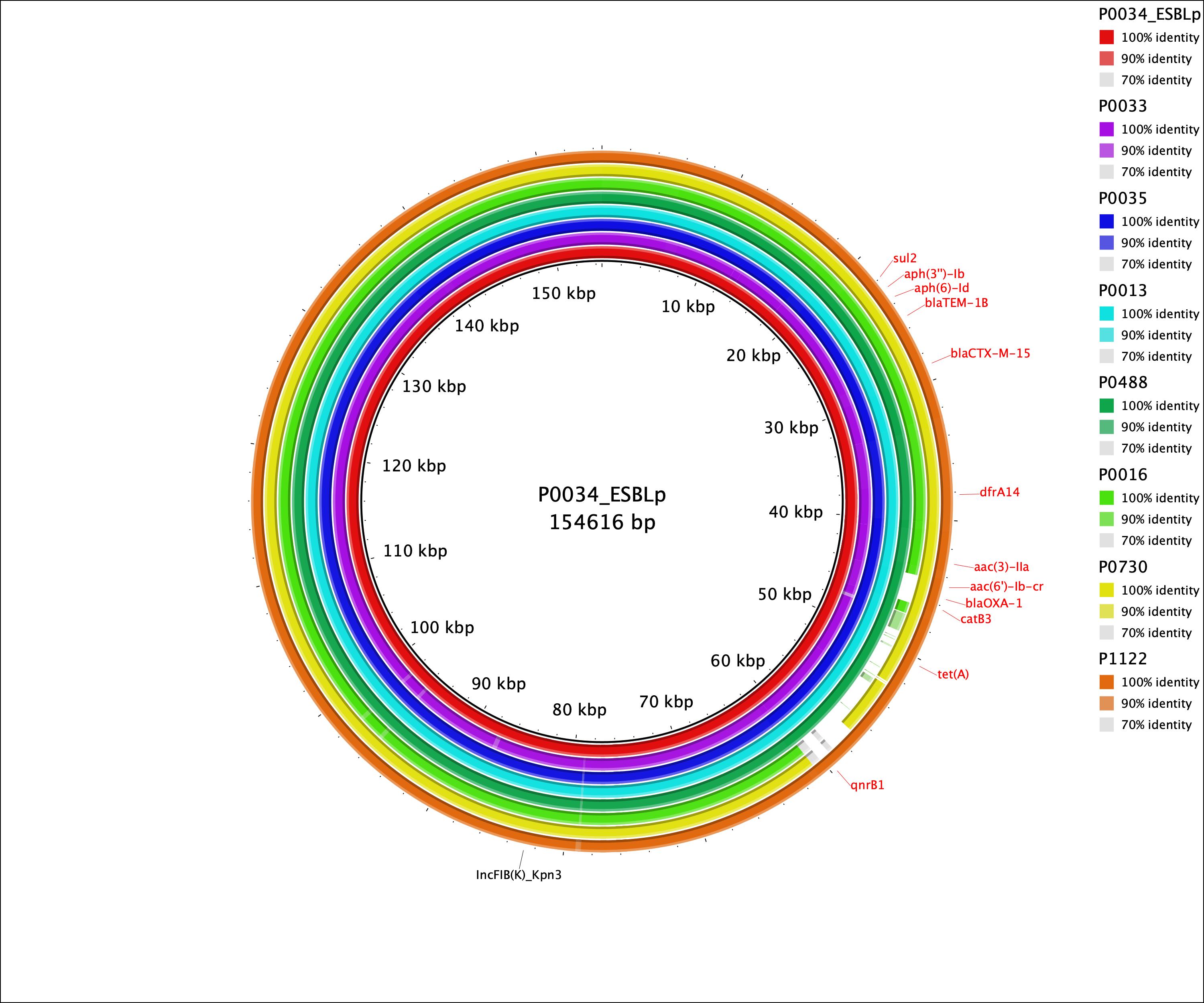

Supplement: Supplementary file 11 — Source Data [file 41467_2023_44285_MOESM11_ESM.zip › SourceDataFile/ESBLp_figures/Kpneumoniae_BRIG_figures_refPacBio_othersIllumina/P0034_ESBLp.jpg]

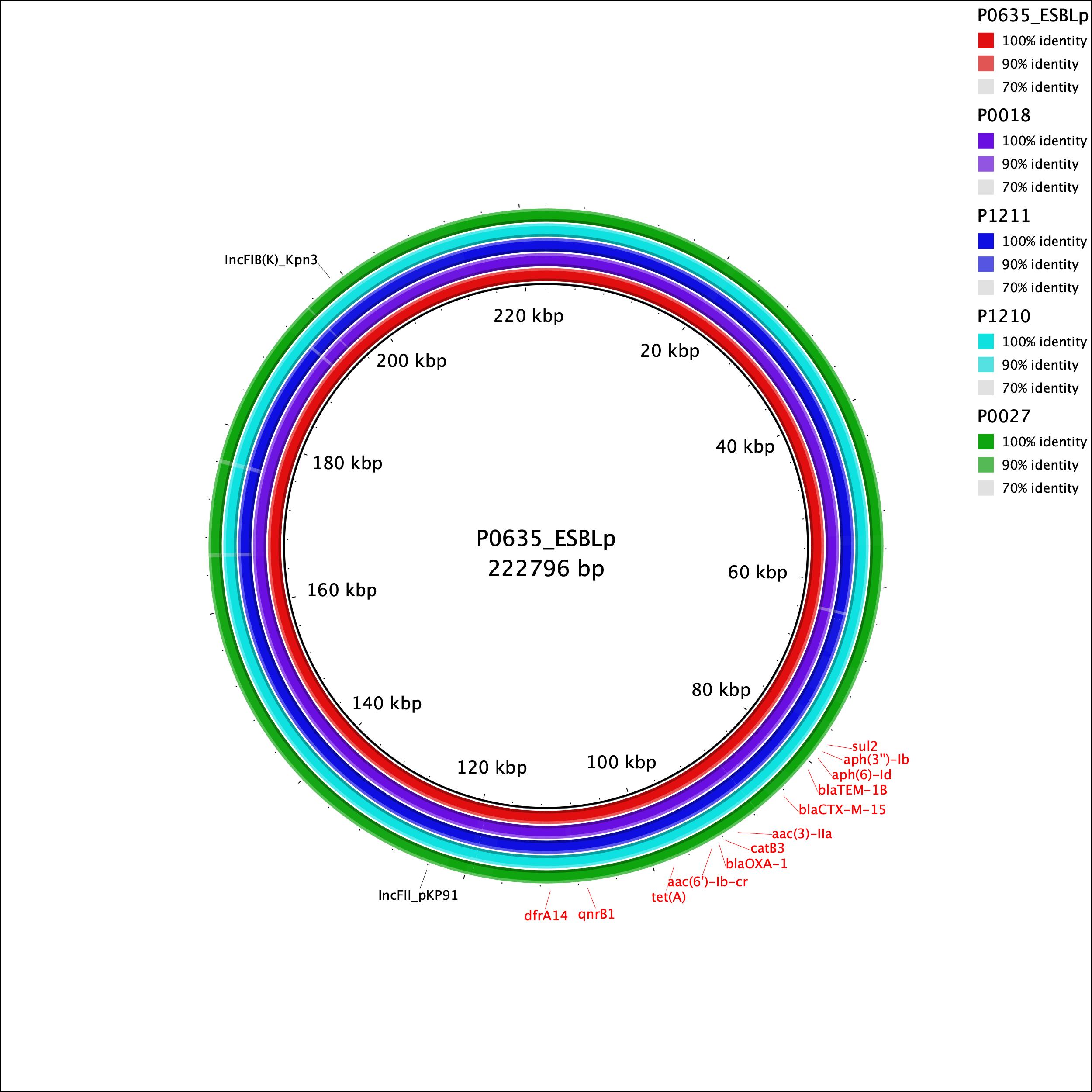

Supplement: Supplementary file 11 — Source Data [file 41467_2023_44285_MOESM11_ESM.zip › SourceDataFile/ESBLp_figures/Kpneumoniae_BRIG_figures_refPacBio_othersIllumina/P0635_ESBLp.jpg]

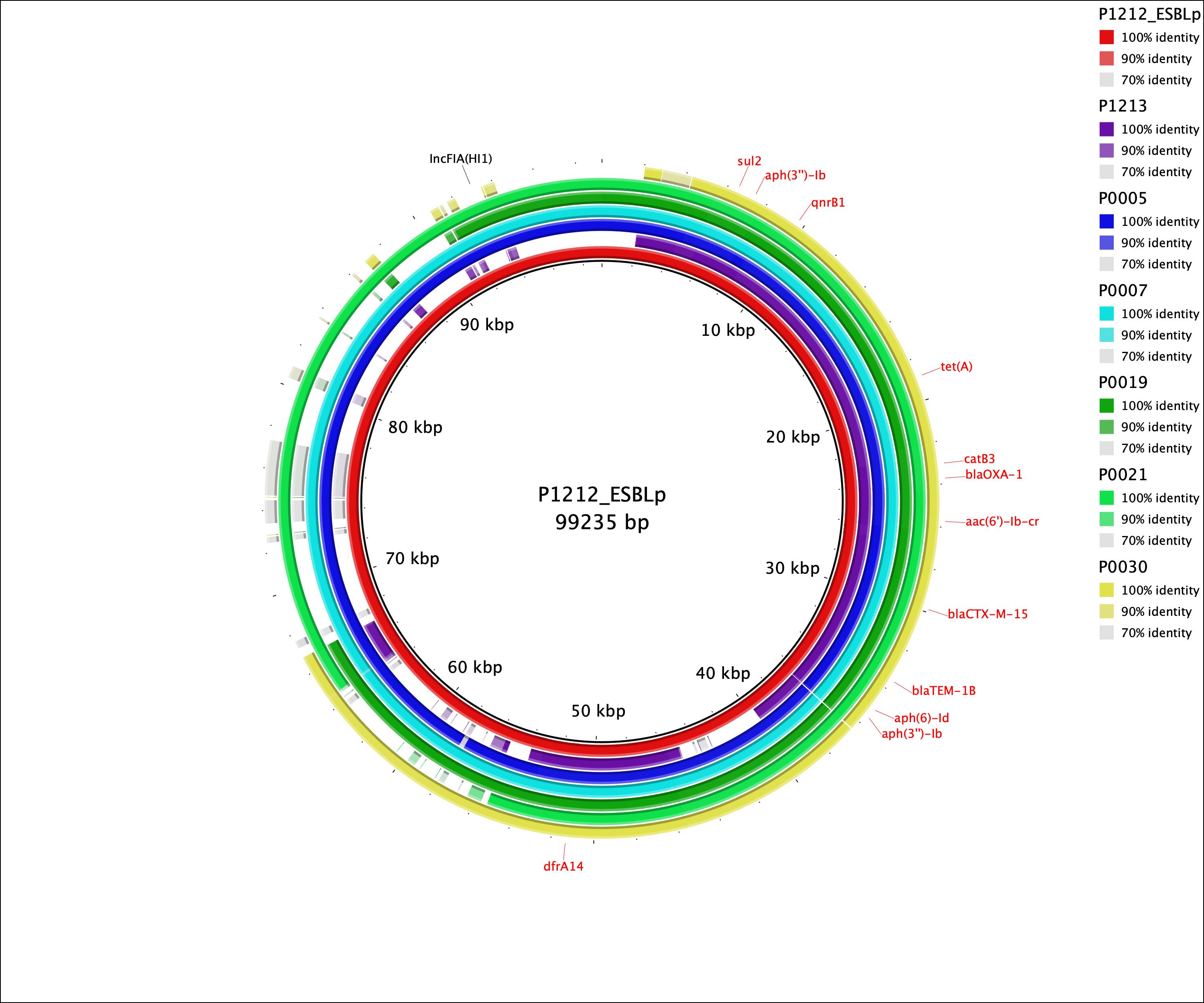

Supplement: Supplementary file 11 — Source Data [file 41467_2023_44285_MOESM11_ESM.zip › SourceDataFile/ESBLp_figures/Kpneumoniae_BRIG_figures_refPacBio_othersIllumina/P1212_ESBLp.fasta.jpg]

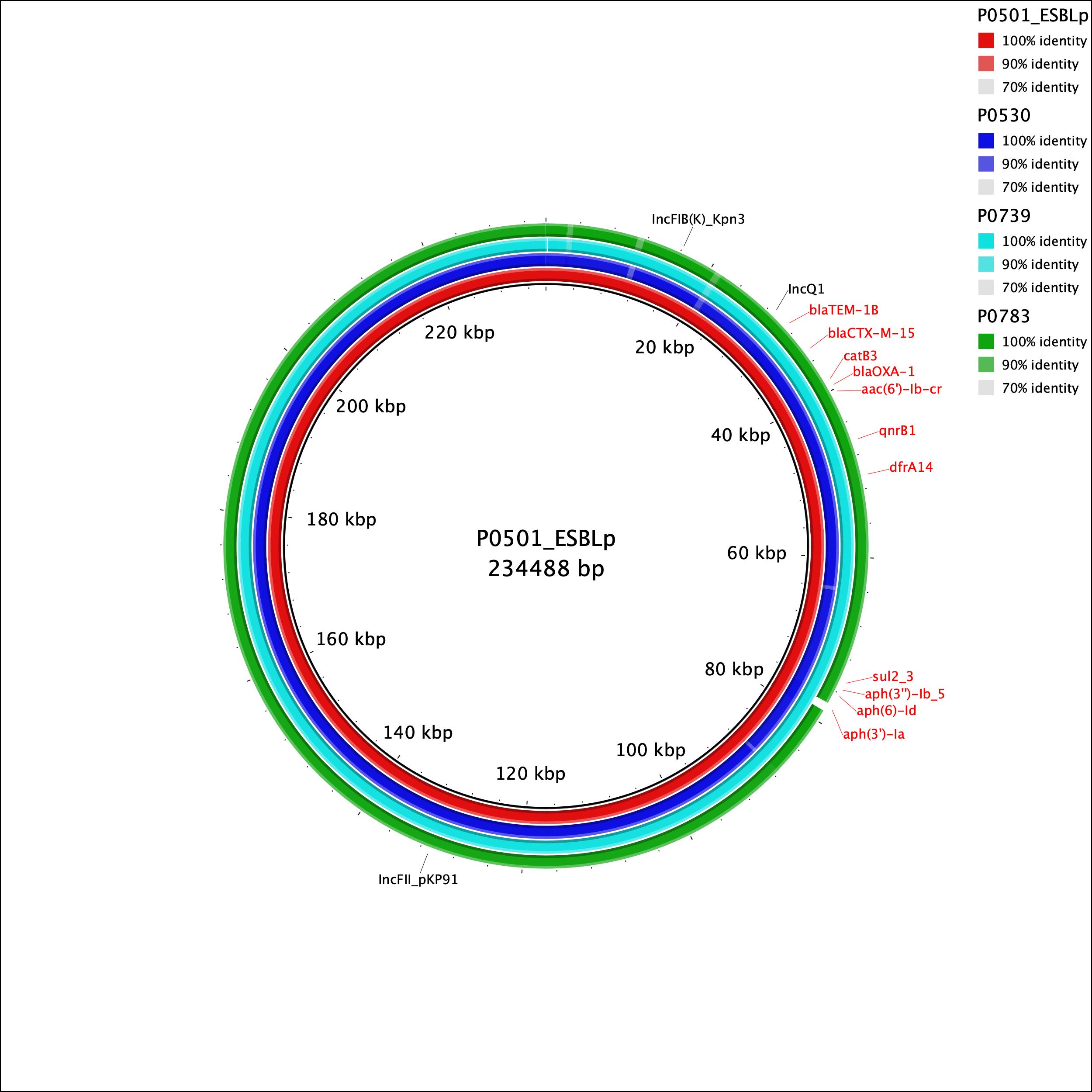

Supplement: Supplementary file 11 — Source Data [file 41467_2023_44285_MOESM11_ESM.zip › SourceDataFile/ESBLp_figures/Kpneumoniae_BRIG_figures_refPacBio_othersIllumina/P0501_ESBLp.jpg]

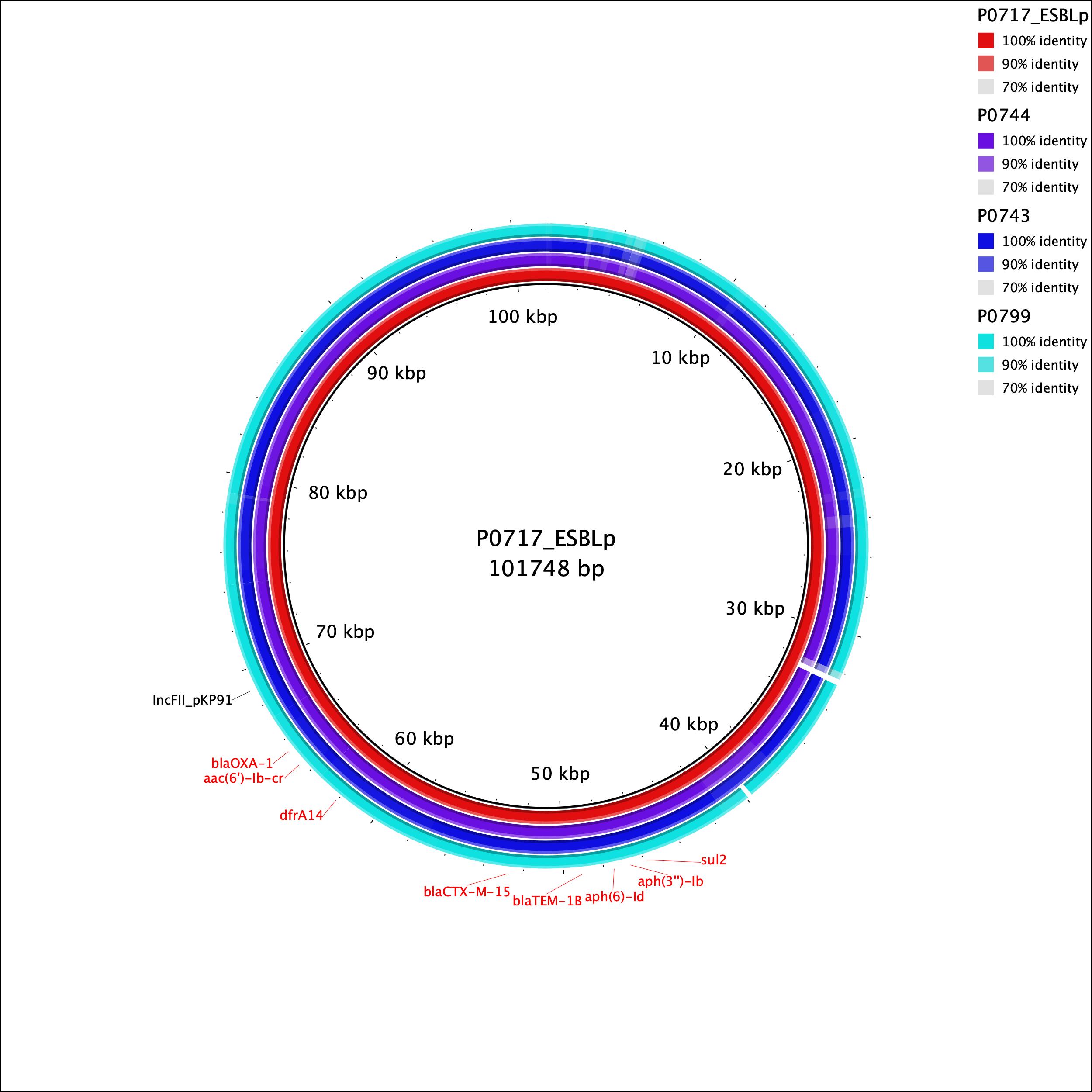

Supplement: Supplementary file 11 — Source Data [file 41467_2023_44285_MOESM11_ESM.zip › SourceDataFile/ESBLp_figures/Kpneumoniae_BRIG_figures_refPacBio_othersIllumina/P0717_ESBLp.jpg]

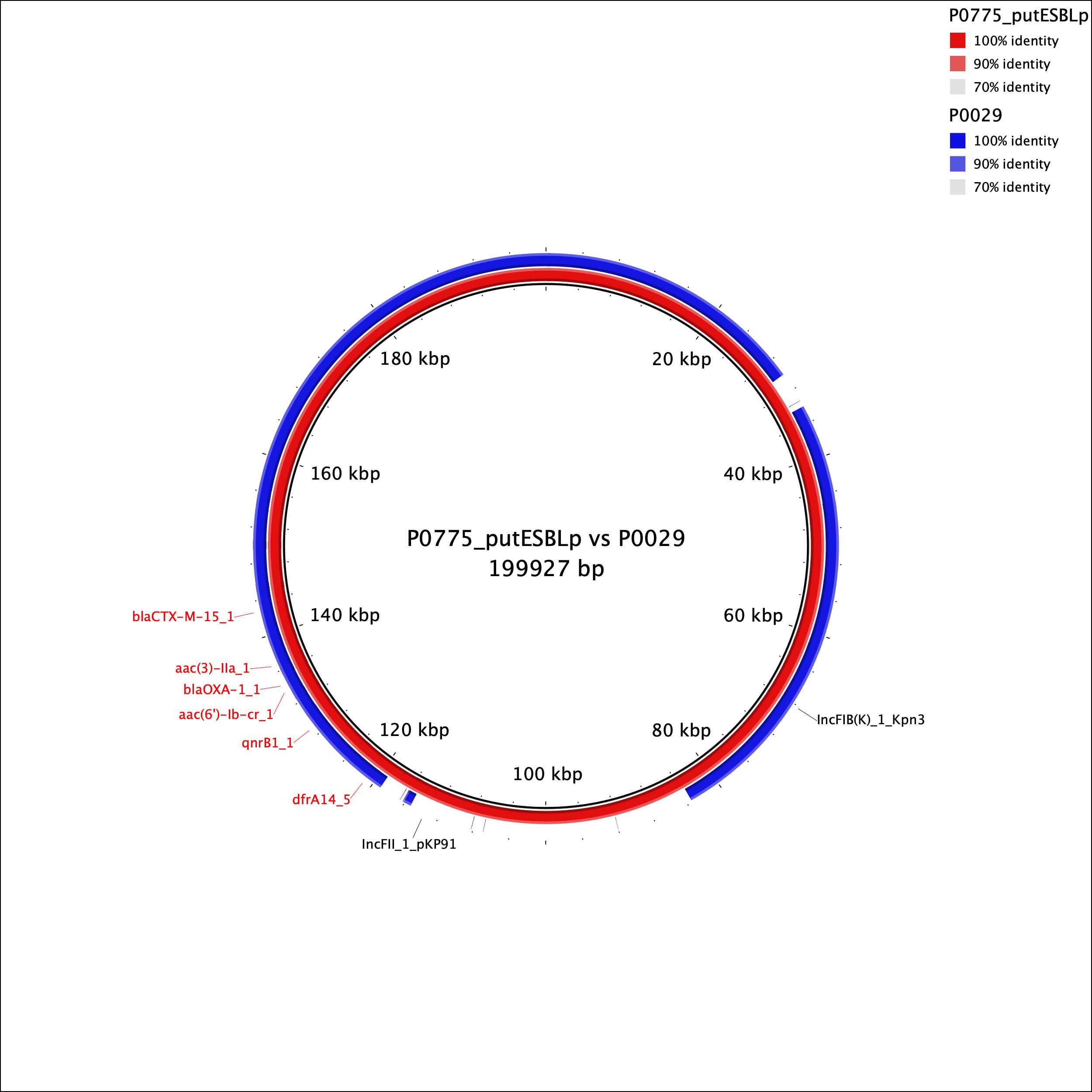

Supplement: Supplementary file 11 — Source Data [file 41467_2023_44285_MOESM11_ESM.zip › SourceDataFile/ESBLp_figures/Kpneumoniae_ESBLp_BRIG_figures_allPacBio/P0775_putESBLp.fasta.jpg]

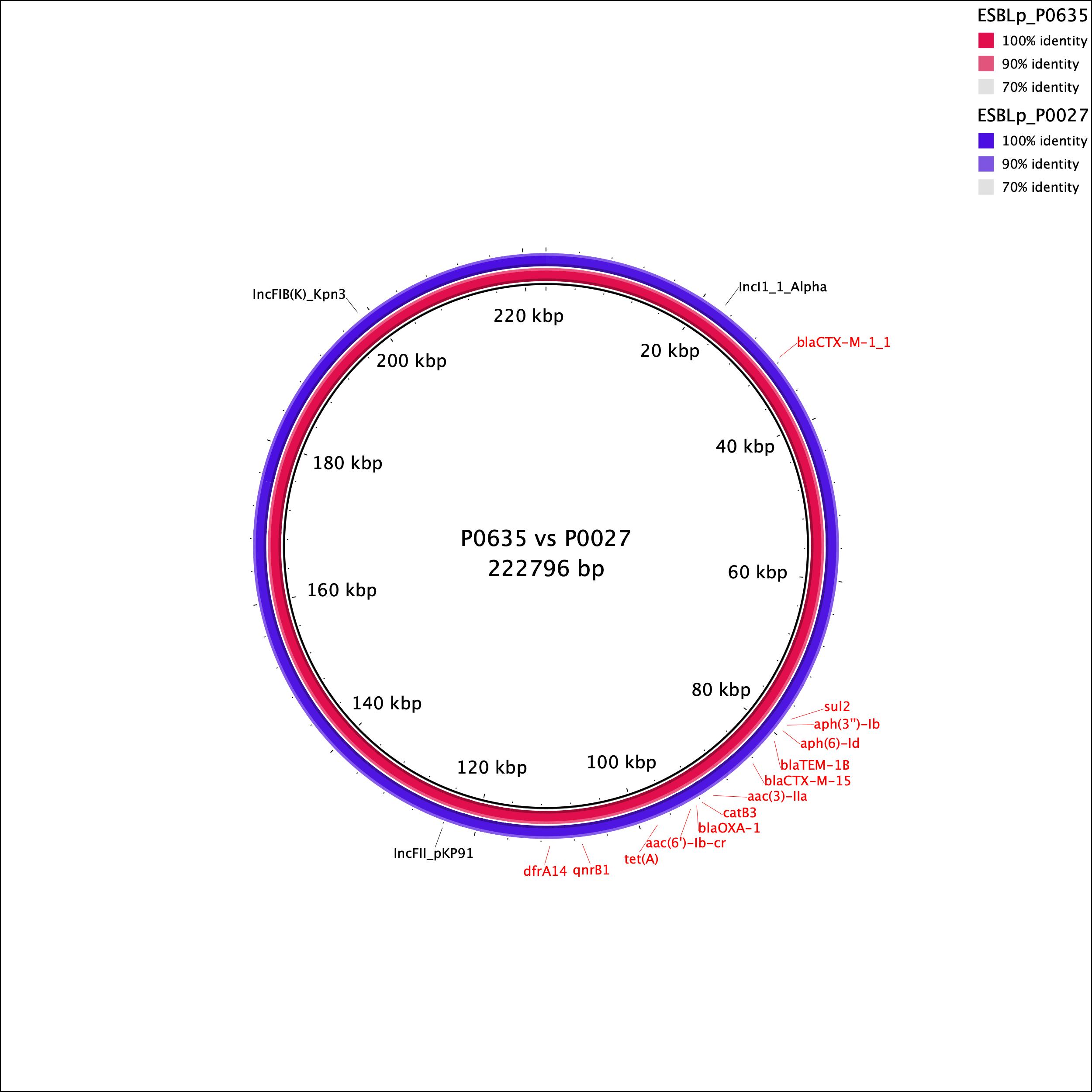

Supplement: Supplementary file 11 — Source Data [file 41467_2023_44285_MOESM11_ESM.zip › SourceDataFile/ESBLp_figures/Kpneumoniae_ESBLp_BRIG_figures_allPacBio/P0635_ESBLp.fasta_comparison1.jpg]

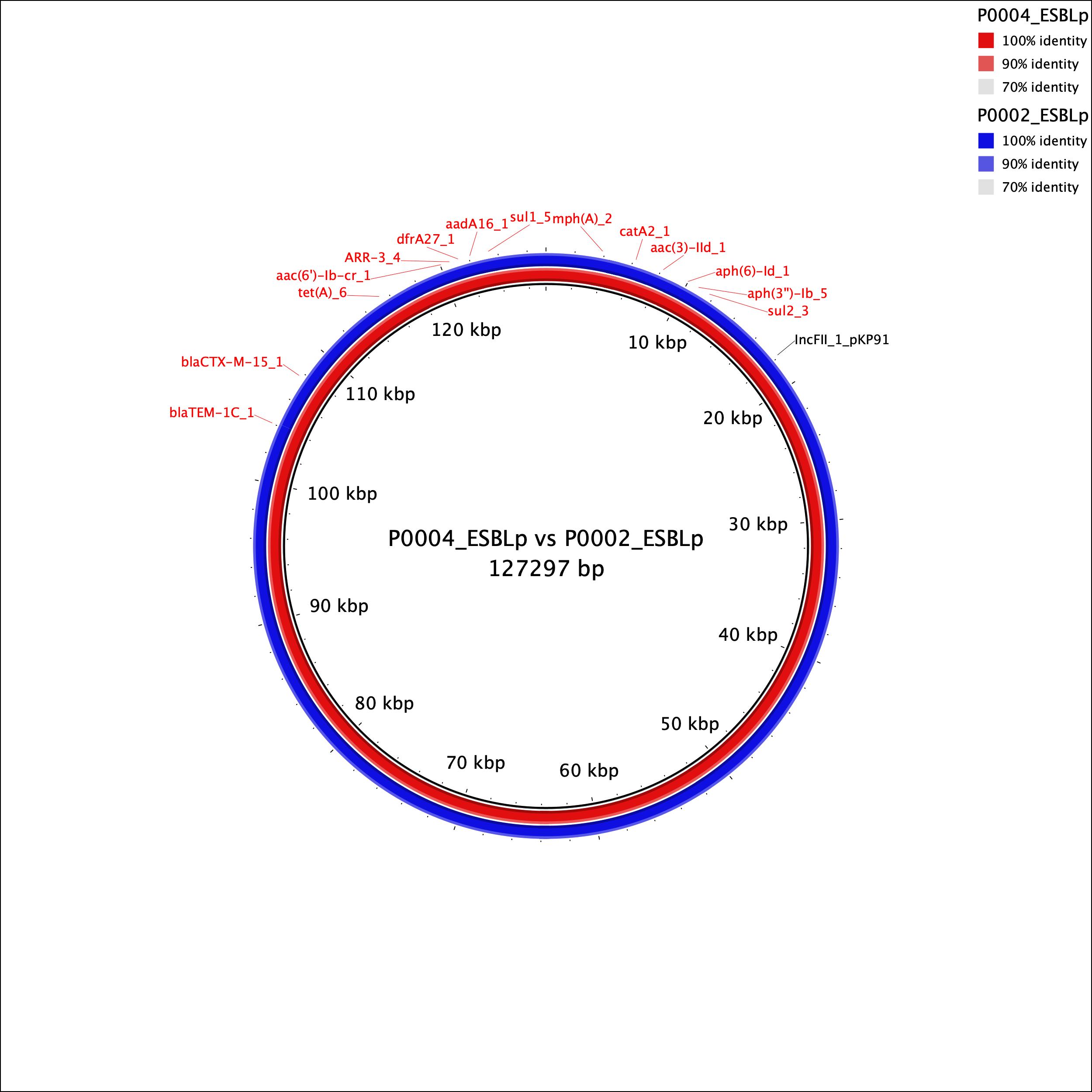

Supplement: Supplementary file 11 — Source Data [file 41467_2023_44285_MOESM11_ESM.zip › SourceDataFile/ESBLp_figures/Kpneumoniae_ESBLp_BRIG_figures_allPacBio/P0004_ESBLp.fasta_comparison2.jpg]

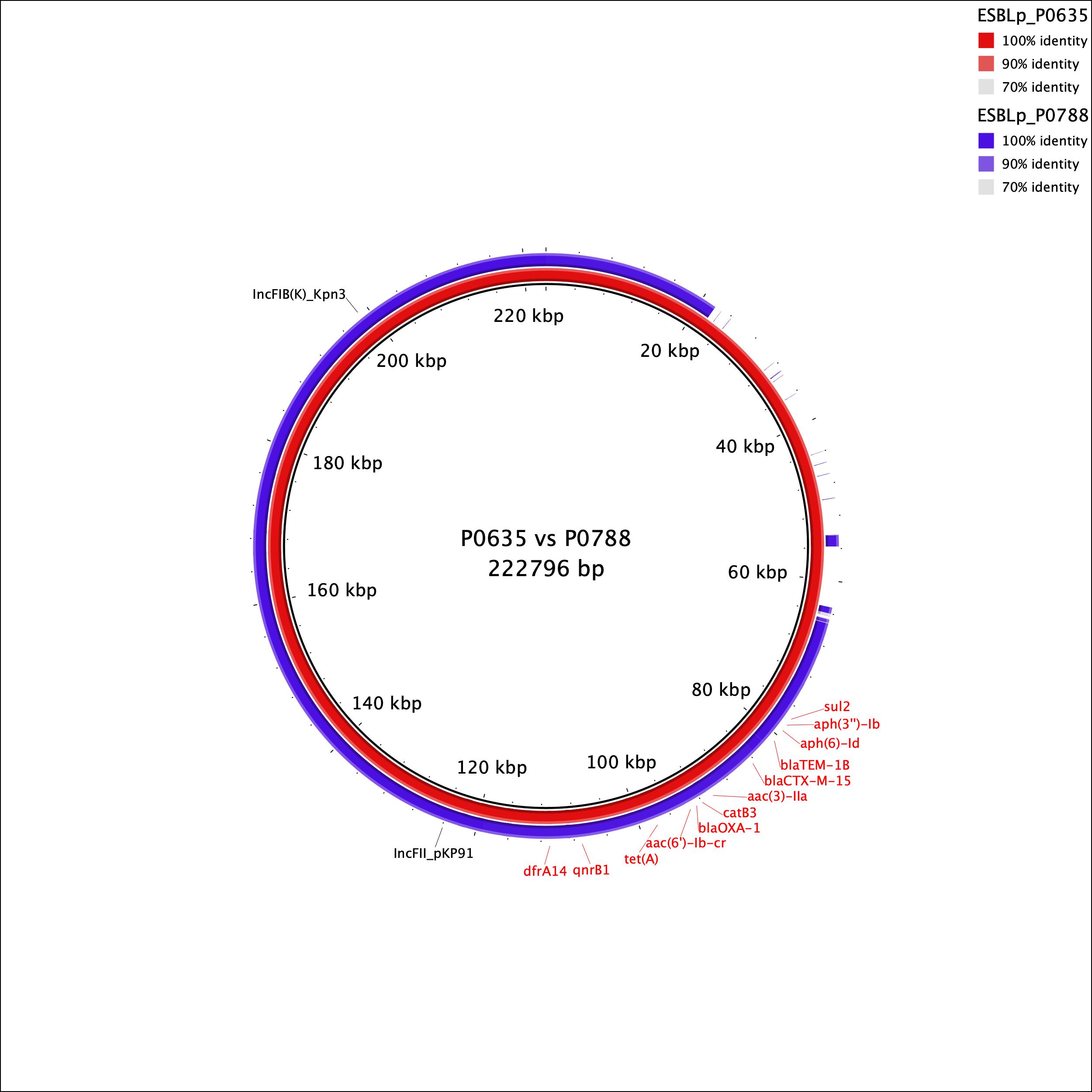

Supplement: Supplementary file 11 — Source Data [file 41467_2023_44285_MOESM11_ESM.zip › SourceDataFile/ESBLp_figures/Kpneumoniae_ESBLp_BRIG_figures_allPacBio/P0635_ESBLp.fasta_comparison2.jpg]

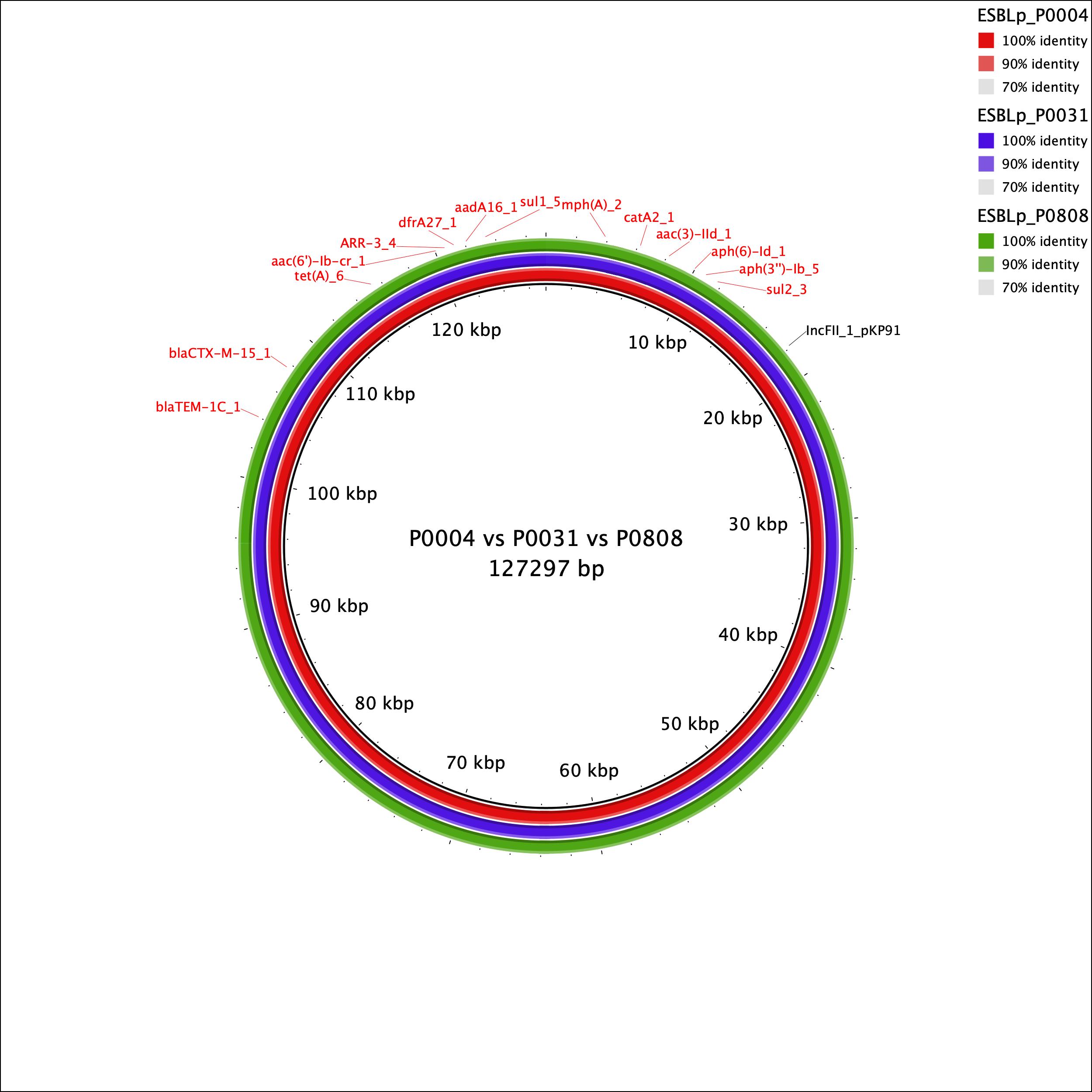

Supplement: Supplementary file 11 — Source Data [file 41467_2023_44285_MOESM11_ESM.zip › SourceDataFile/ESBLp_figures/Kpneumoniae_ESBLp_BRIG_figures_allPacBio/P0004_ESBLp.fasta_comparison1.jpg]

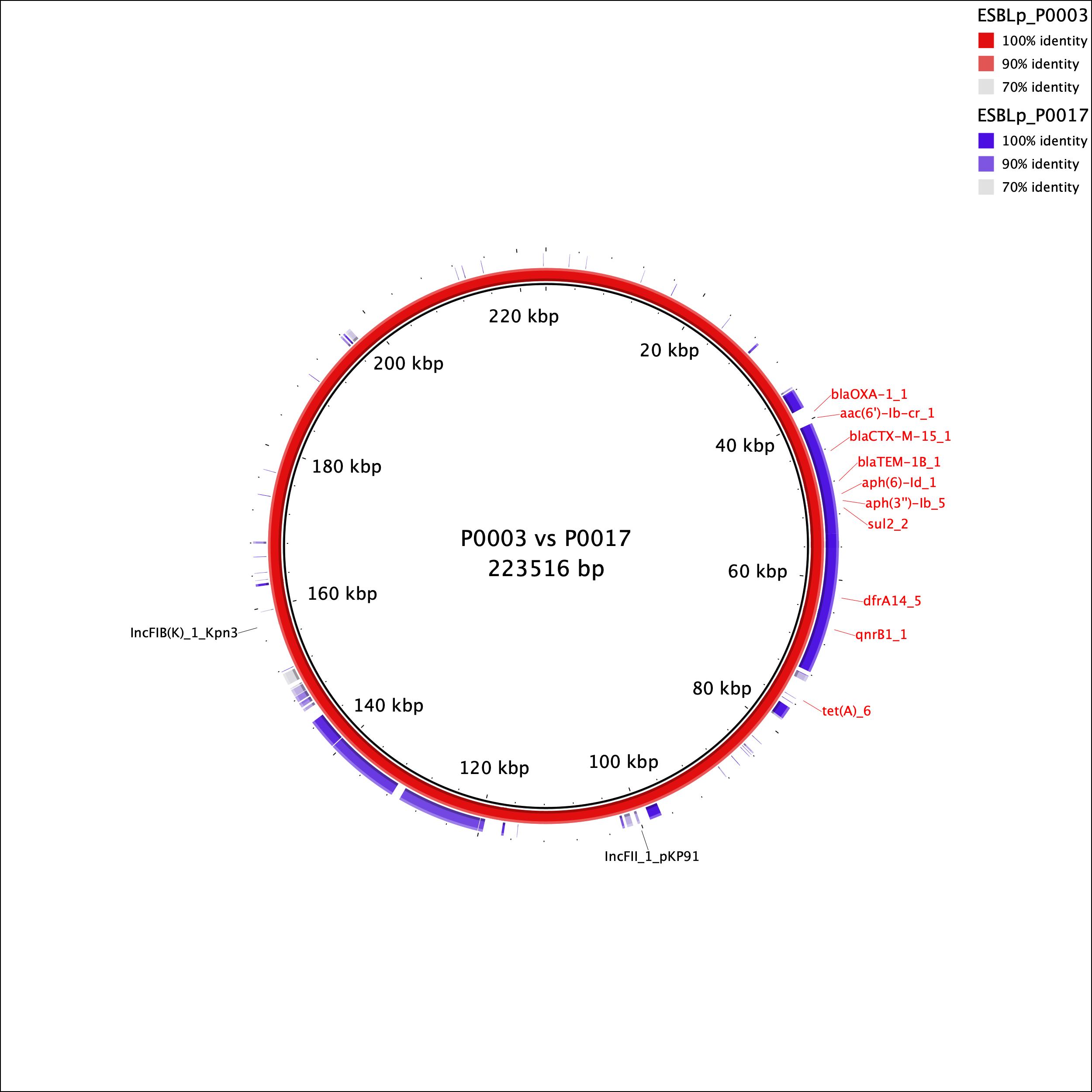

Supplement: Supplementary file 11 — Source Data [file 41467_2023_44285_MOESM11_ESM.zip › SourceDataFile/ESBLp_figures/Kpneumoniae_ESBLp_BRIG_figures_allPacBio/P0003_ESBLp.fasta.jpg]

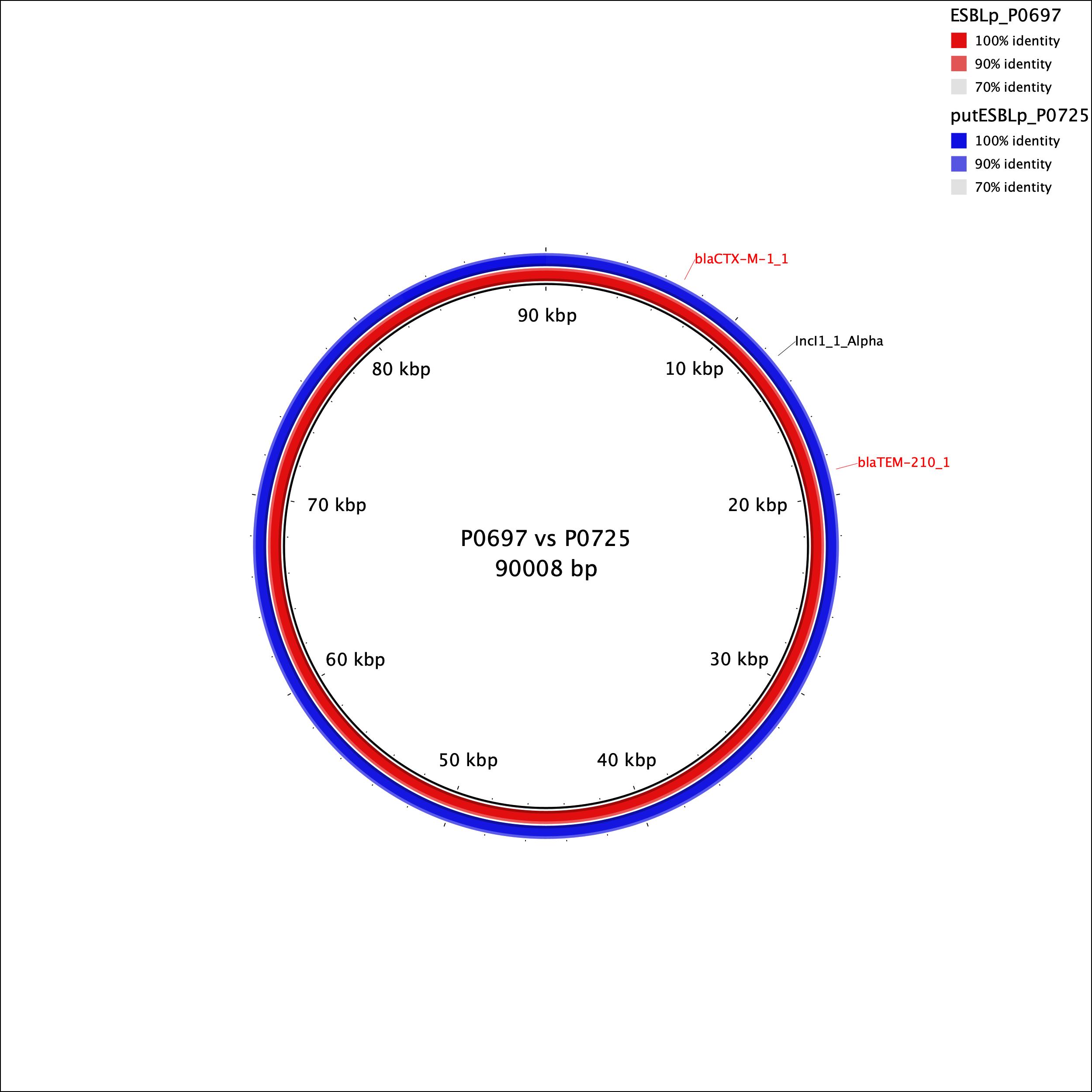

Supplement: Supplementary file 11 — Source Data [file 41467_2023_44285_MOESM11_ESM.zip › SourceDataFile/ESBLp_figures/Kpneumoniae_ESBLp_BRIG_figures_allPacBio/P0697_ESBLp.fasta.jpg]

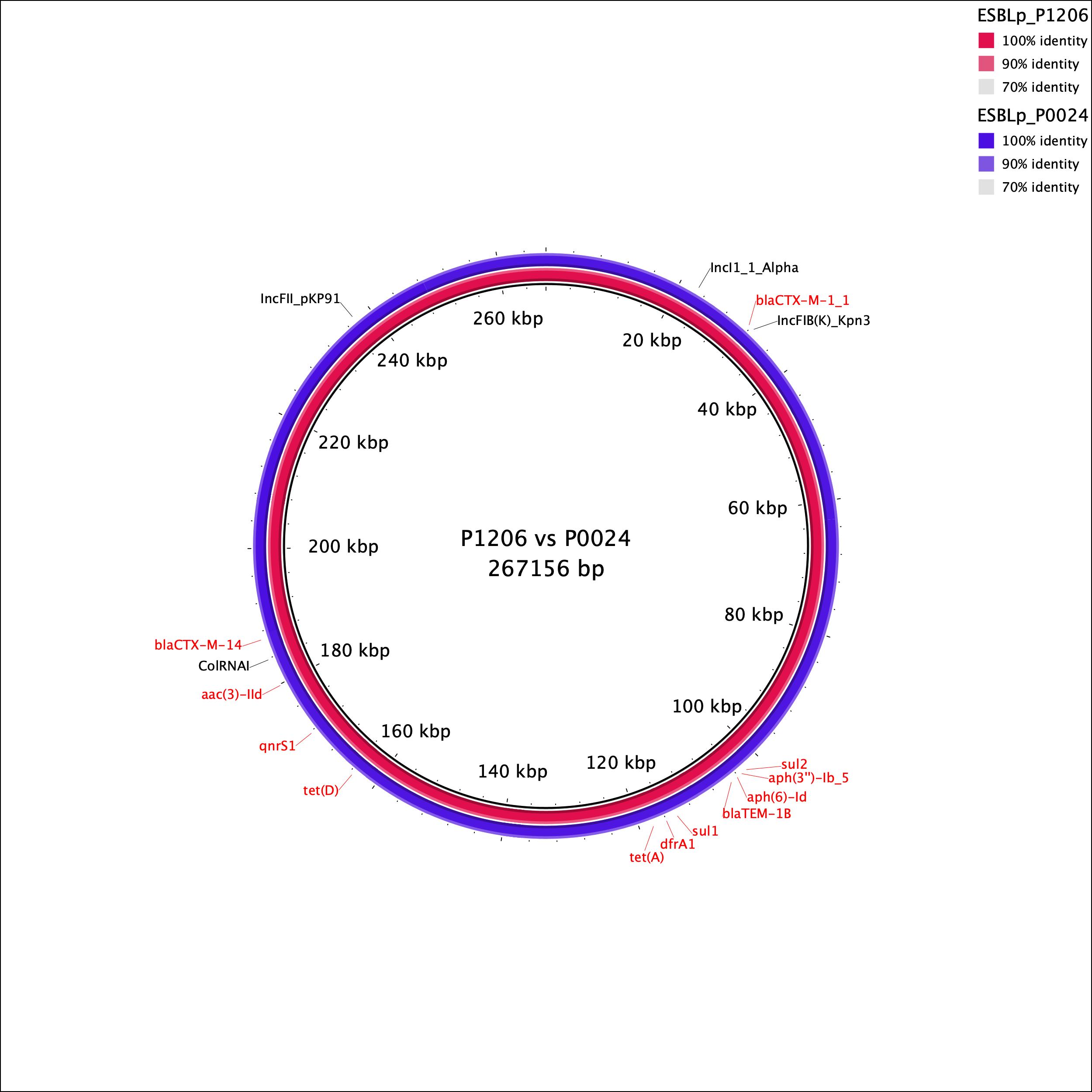

Supplement: Supplementary file 11 — Source Data [file 41467_2023_44285_MOESM11_ESM.zip › SourceDataFile/ESBLp_figures/Kpneumoniae_ESBLp_BRIG_figures_allPacBio/P1206_ESBLp.fasta.jpg]

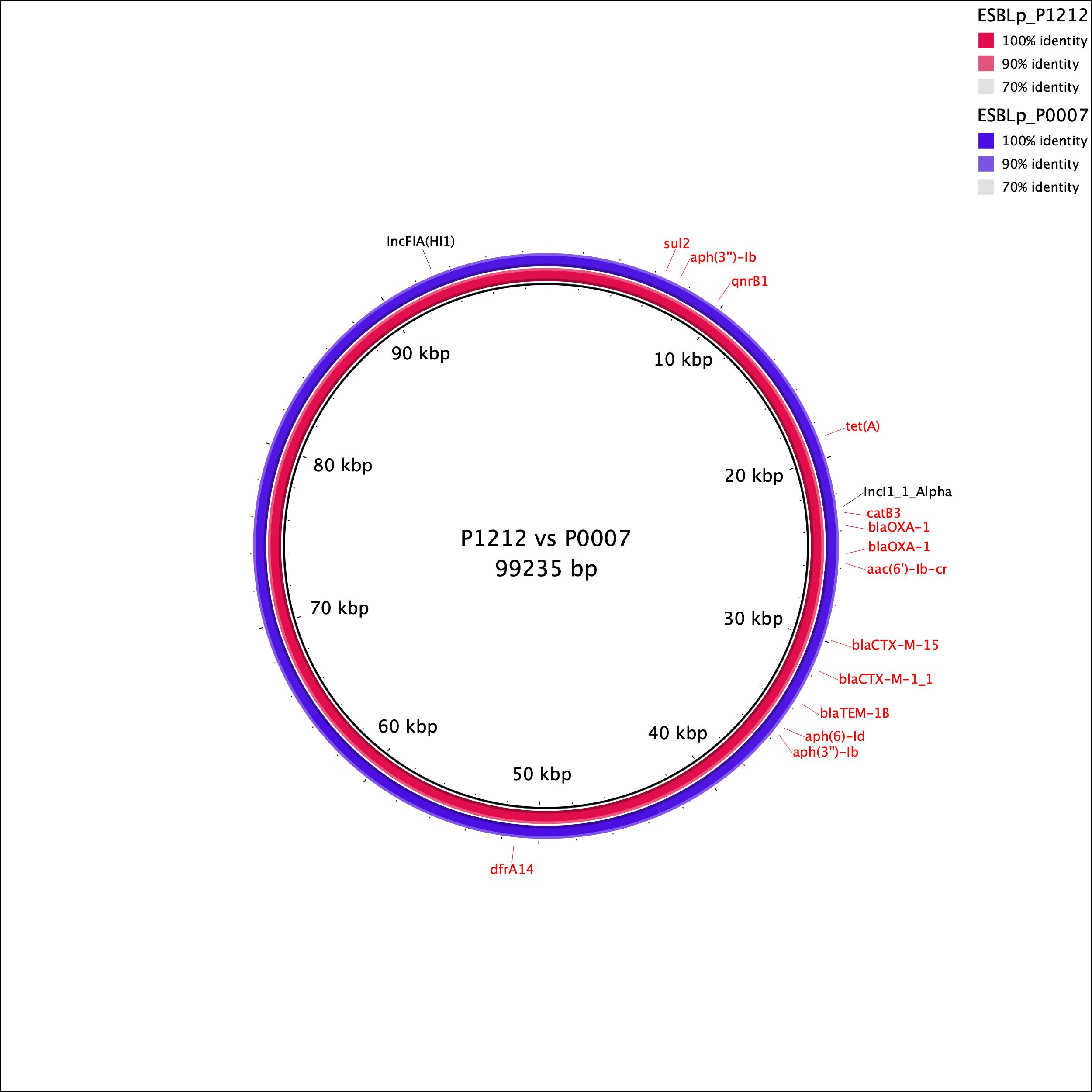

Supplement: Supplementary file 11 — Source Data [file 41467_2023_44285_MOESM11_ESM.zip › SourceDataFile/ESBLp_figures/Kpneumoniae_ESBLp_BRIG_figures_allPacBio/P1212_ESBLp.fasta.jpg]

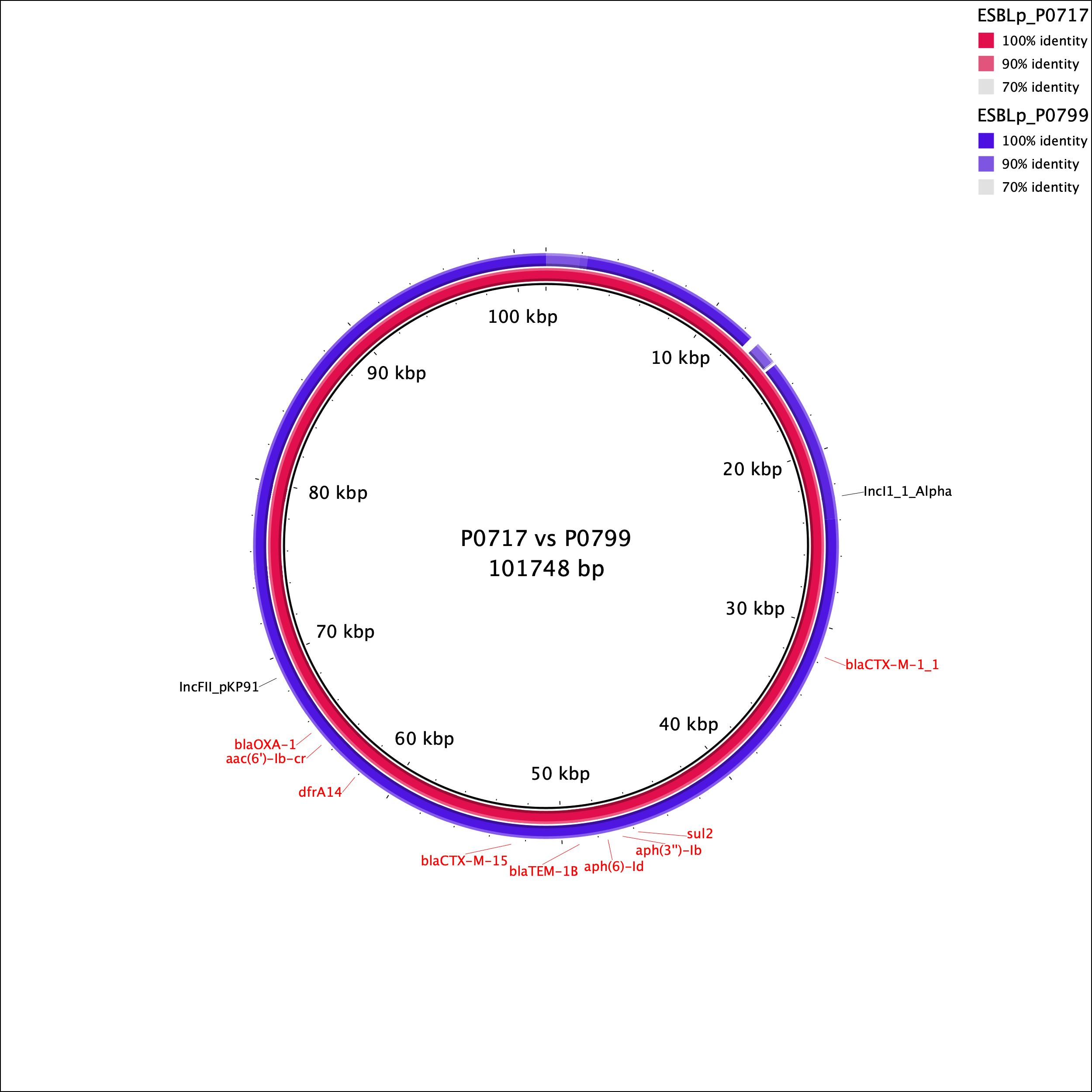

Supplement: Supplementary file 11 — Source Data [file 41467_2023_44285_MOESM11_ESM.zip › SourceDataFile/ESBLp_figures/Kpneumoniae_ESBLp_BRIG_figures_allPacBio/P0717_ESBLp.fasta.jpg]

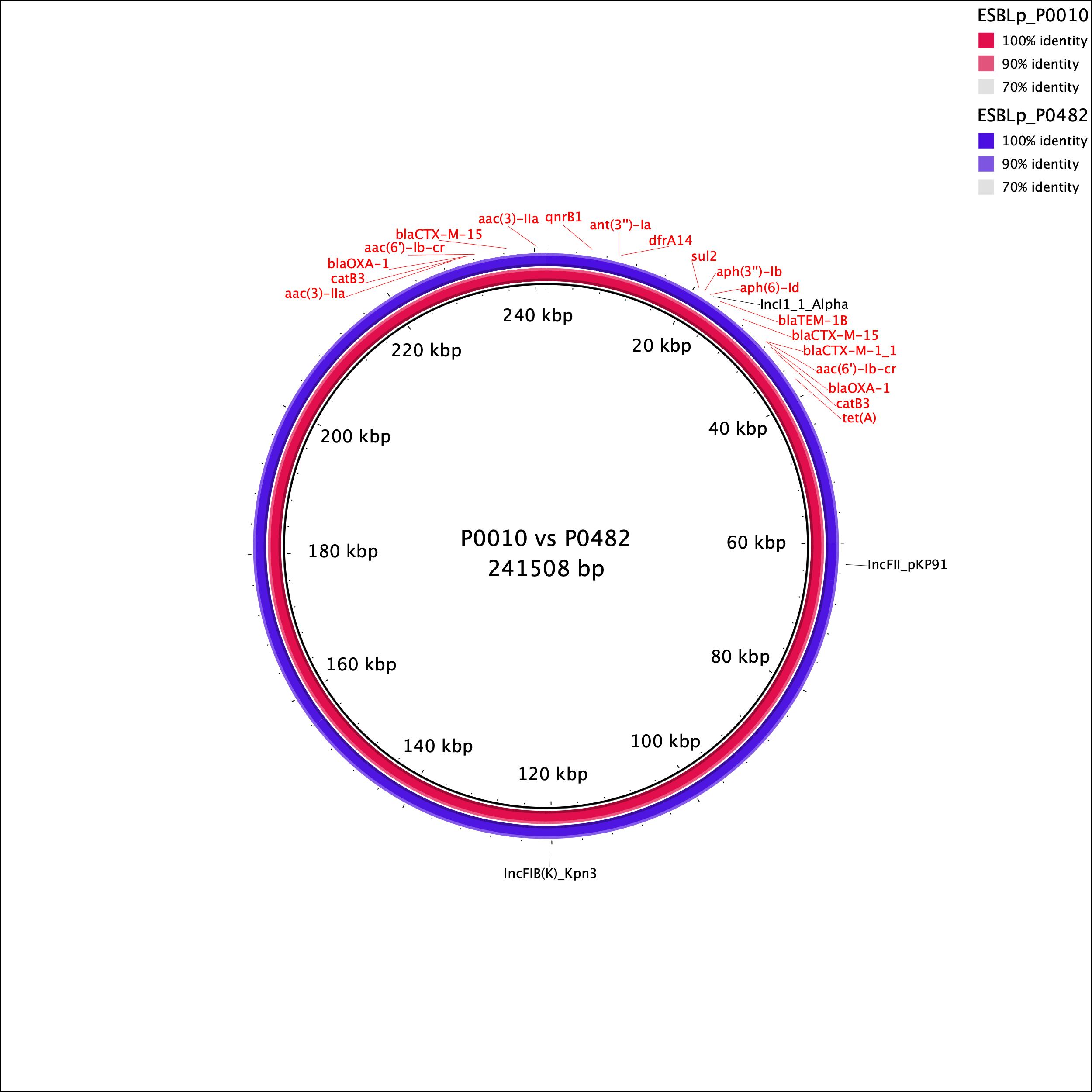

Supplement: Supplementary file 11 — Source Data [file 41467_2023_44285_MOESM11_ESM.zip › SourceDataFile/ESBLp_figures/Kpneumoniae_ESBLp_BRIG_figures_allPacBio/P0010_ESBLp.fasta.jpg]

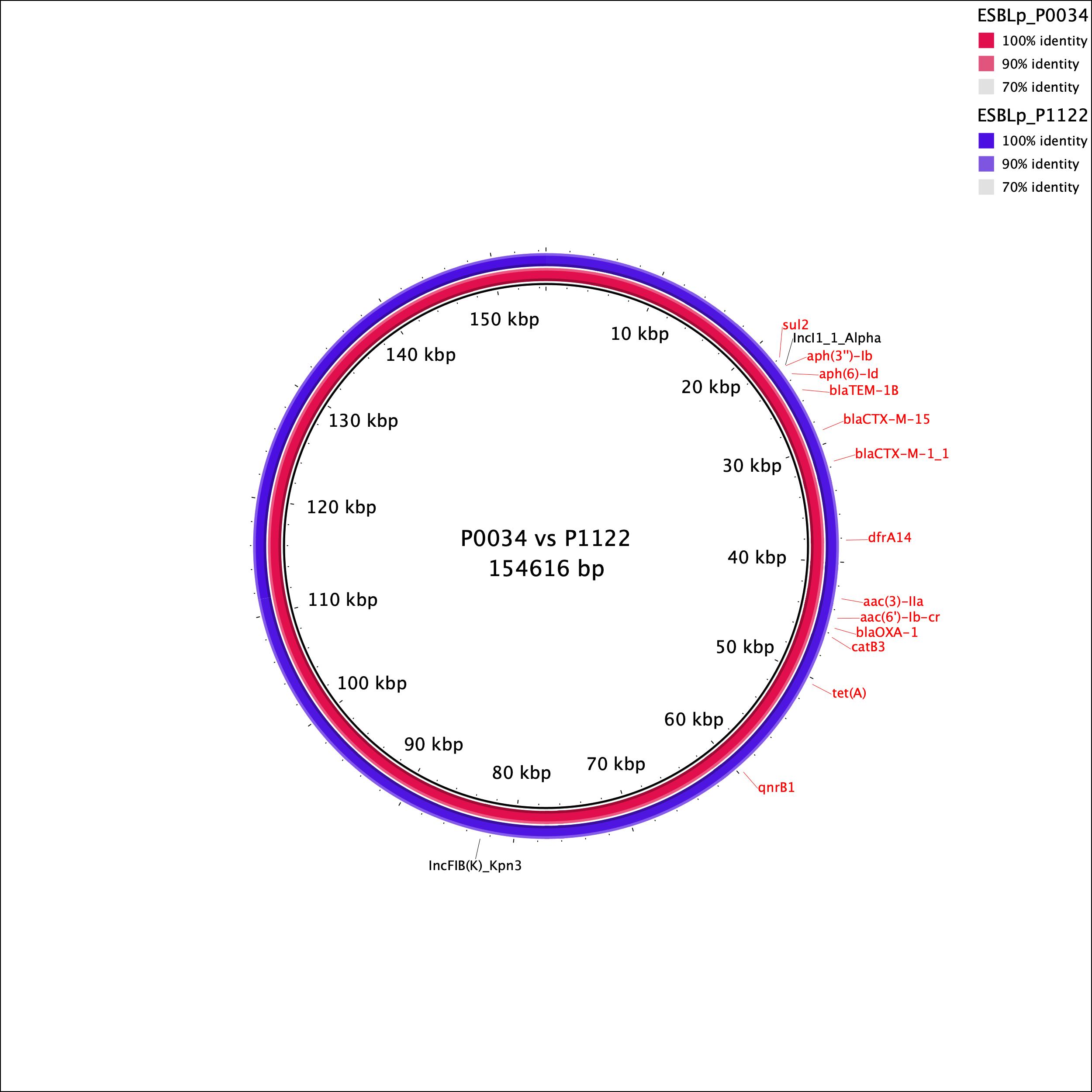

Supplement: Supplementary file 11 — Source Data [file 41467_2023_44285_MOESM11_ESM.zip › SourceDataFile/ESBLp_figures/Kpneumoniae_ESBLp_BRIG_figures_allPacBio/P0034_ESBLp.fasta.jpg]

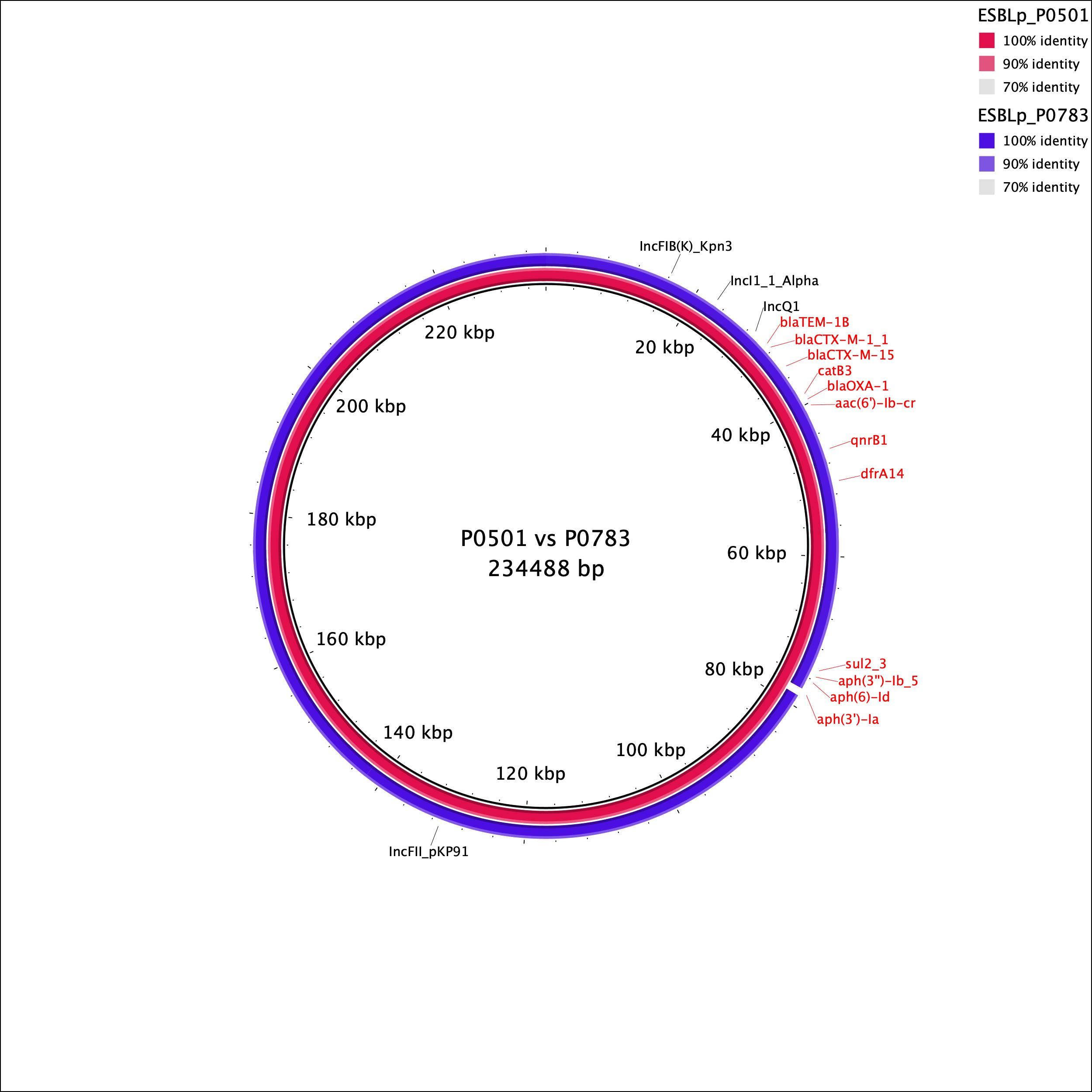

Supplement: Supplementary file 11 — Source Data [file 41467_2023_44285_MOESM11_ESM.zip › SourceDataFile/ESBLp_figures/Kpneumoniae_ESBLp_BRIG_figures_allPacBio/P0501_ESBLp.fasta.jpg]

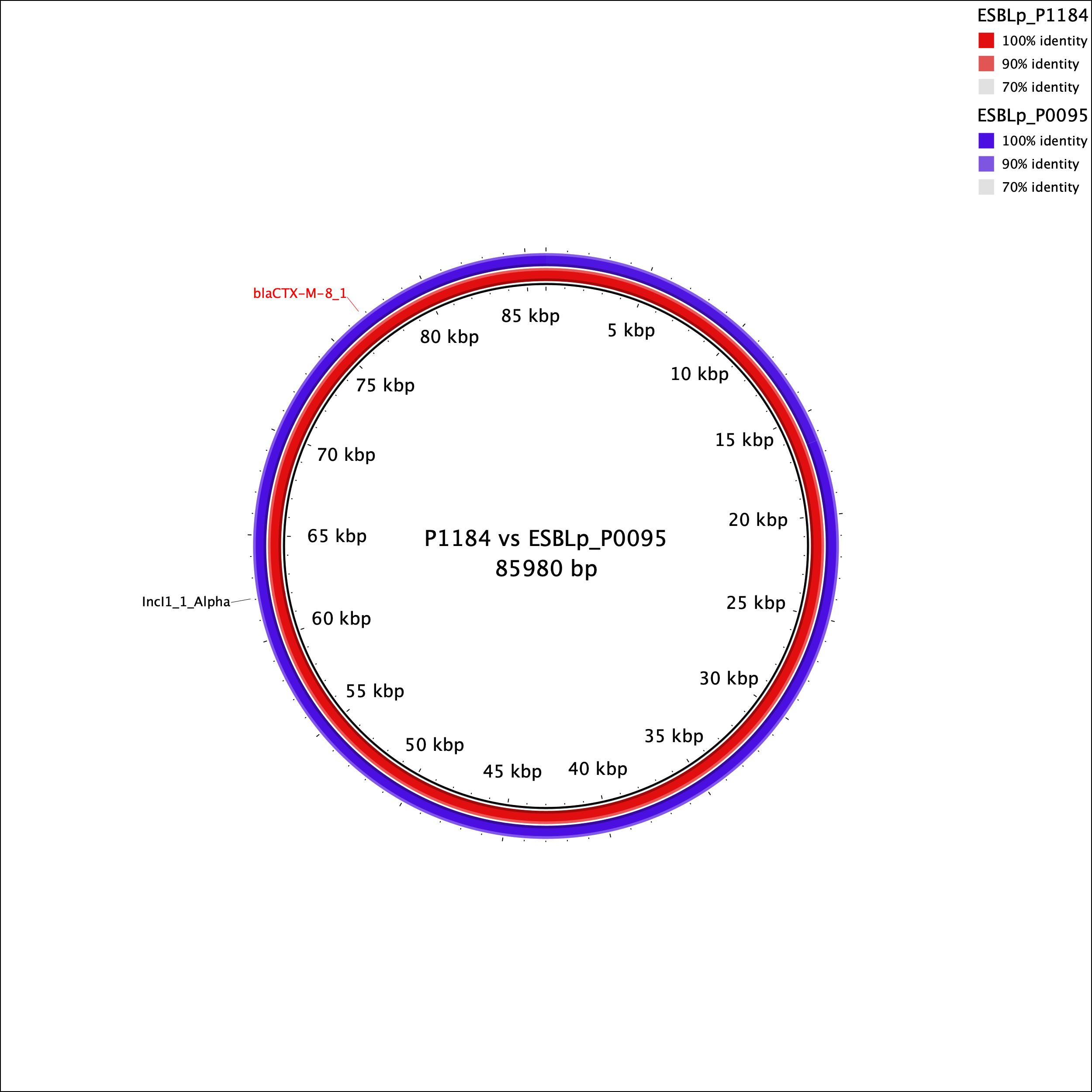

Supplement: Supplementary file 11 — Source Data [file 41467_2023_44285_MOESM11_ESM.zip › SourceDataFile/ESBLp_figures/Ecoli_ESBLp_BRIG_figures_allPacBio/P1184_ESBLp.fasta.jpg]

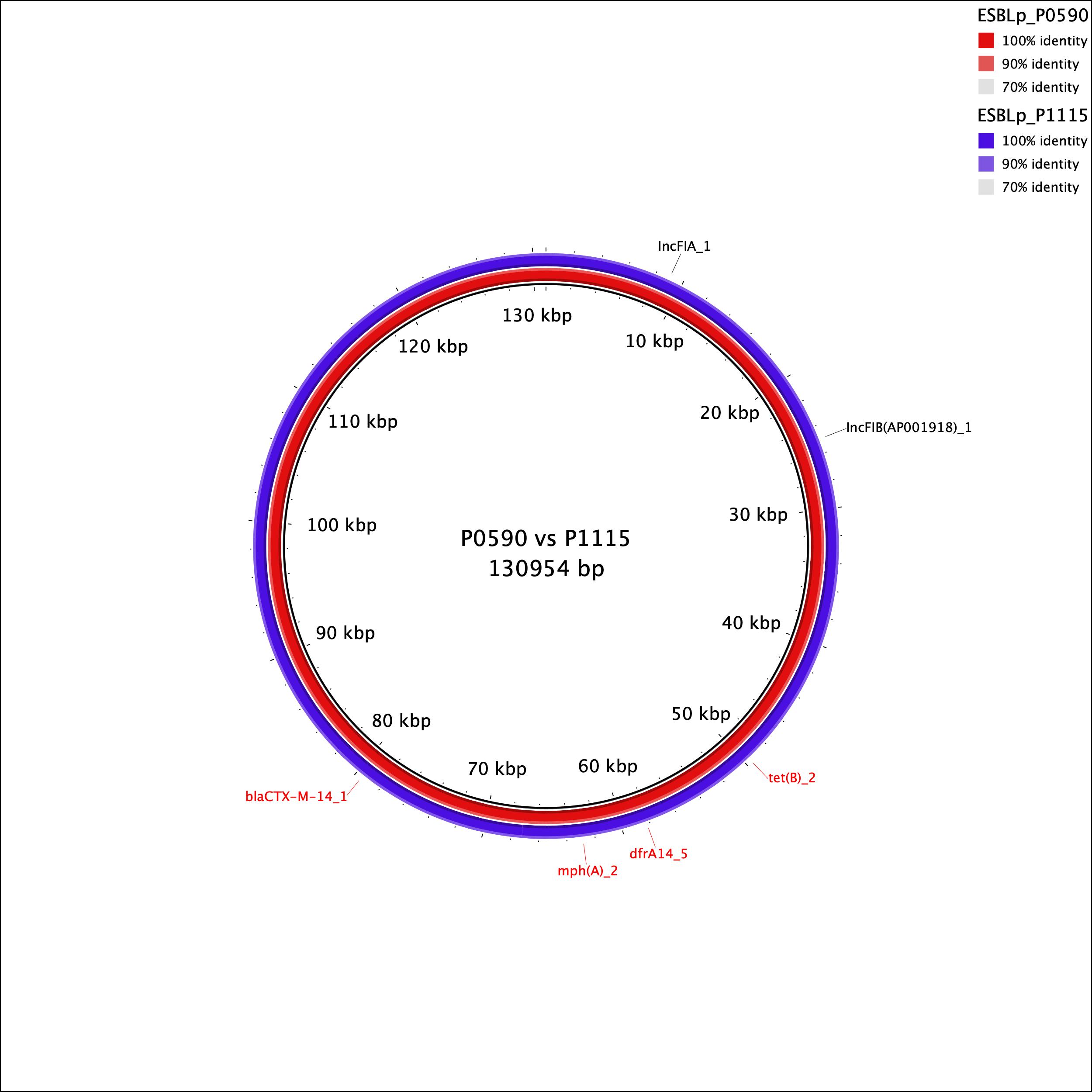

Supplement: Supplementary file 11 — Source Data [file 41467_2023_44285_MOESM11_ESM.zip › SourceDataFile/ESBLp_figures/Ecoli_ESBLp_BRIG_figures_allPacBio/P0590_ESBLp.fasta.jpg]

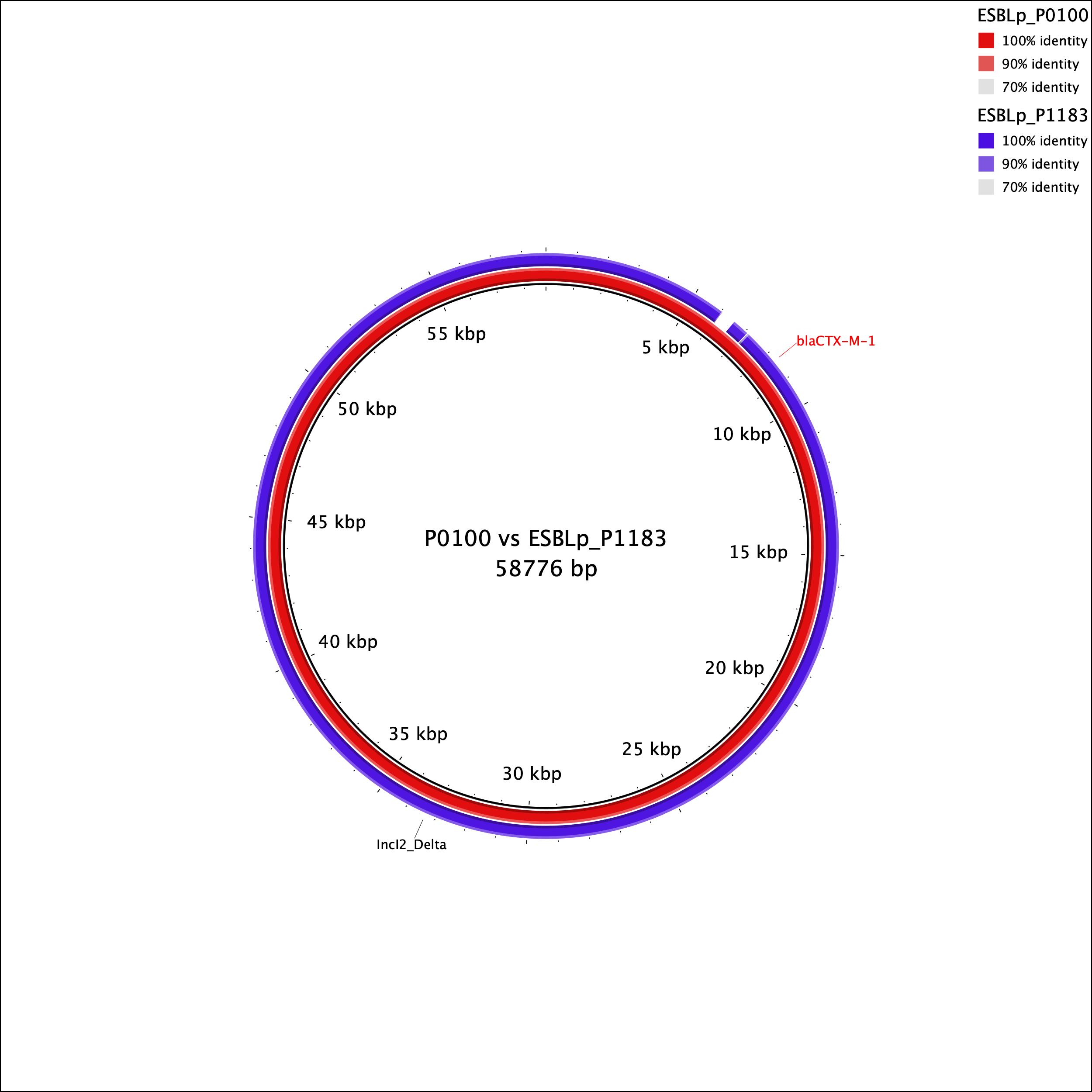

Supplement: Supplementary file 11 — Source Data [file 41467_2023_44285_MOESM11_ESM.zip › SourceDataFile/ESBLp_figures/Ecoli_ESBLp_BRIG_figures_allPacBio/P0100_ESBLp.fasta_comparison1.jpg]

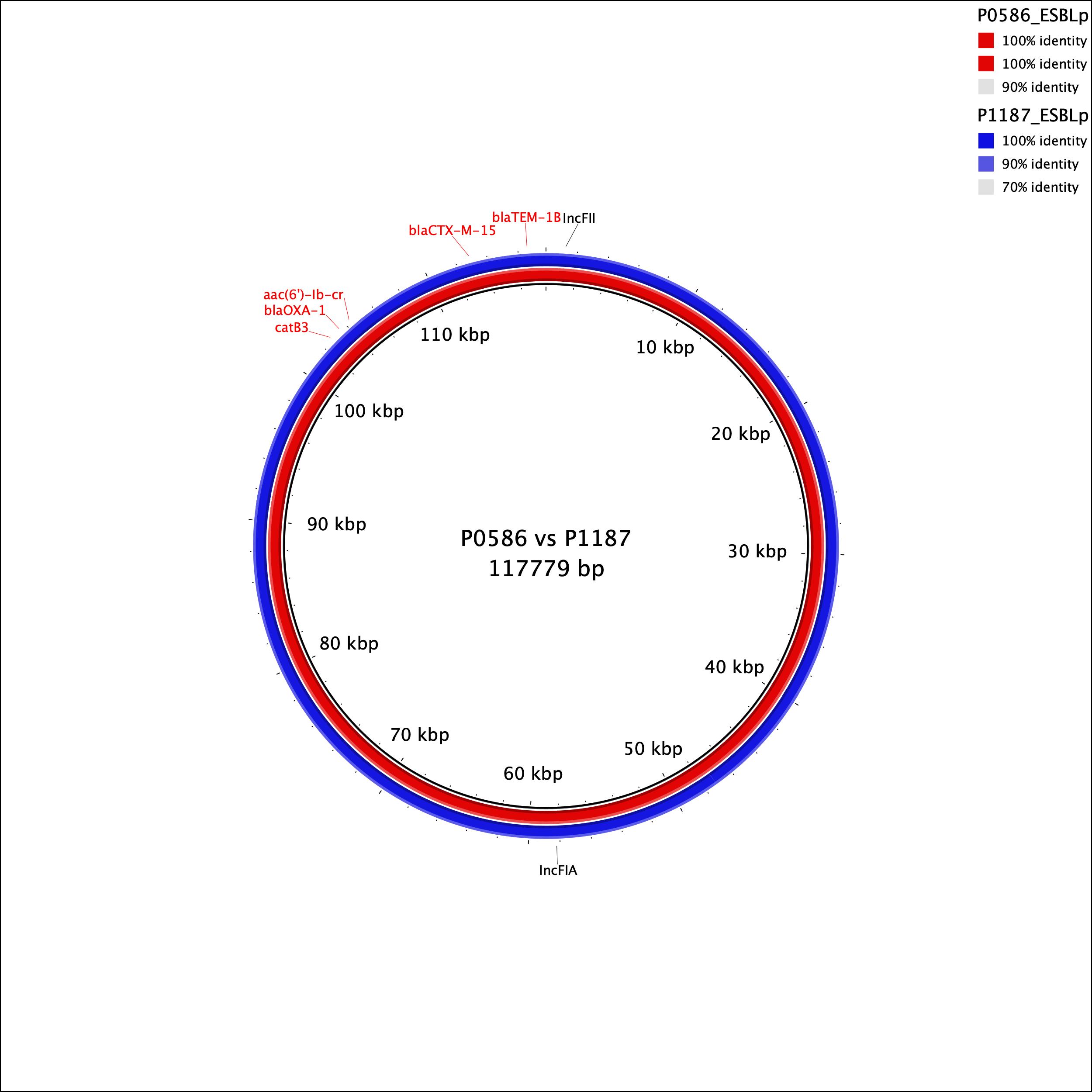

Supplement: Supplementary file 11 — Source Data [file 41467_2023_44285_MOESM11_ESM.zip › SourceDataFile/ESBLp_figures/Ecoli_ESBLp_BRIG_figures_allPacBio/P0586_ESBLp.fasta.jpg]

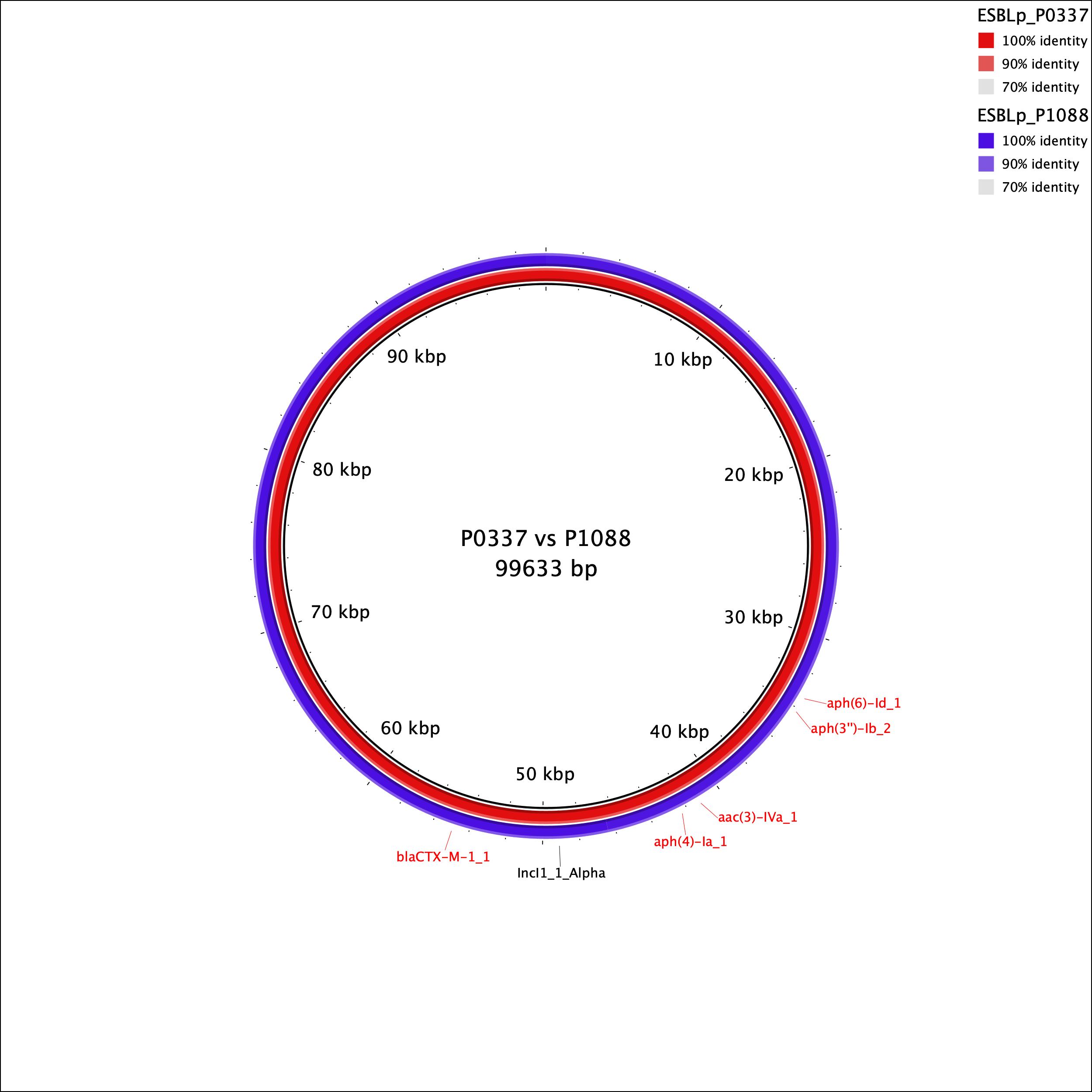

Supplement: Supplementary file 11 — Source Data [file 41467_2023_44285_MOESM11_ESM.zip › SourceDataFile/ESBLp_figures/Ecoli_ESBLp_BRIG_figures_allPacBio/P0337_ESBLp.fasta.jpg]

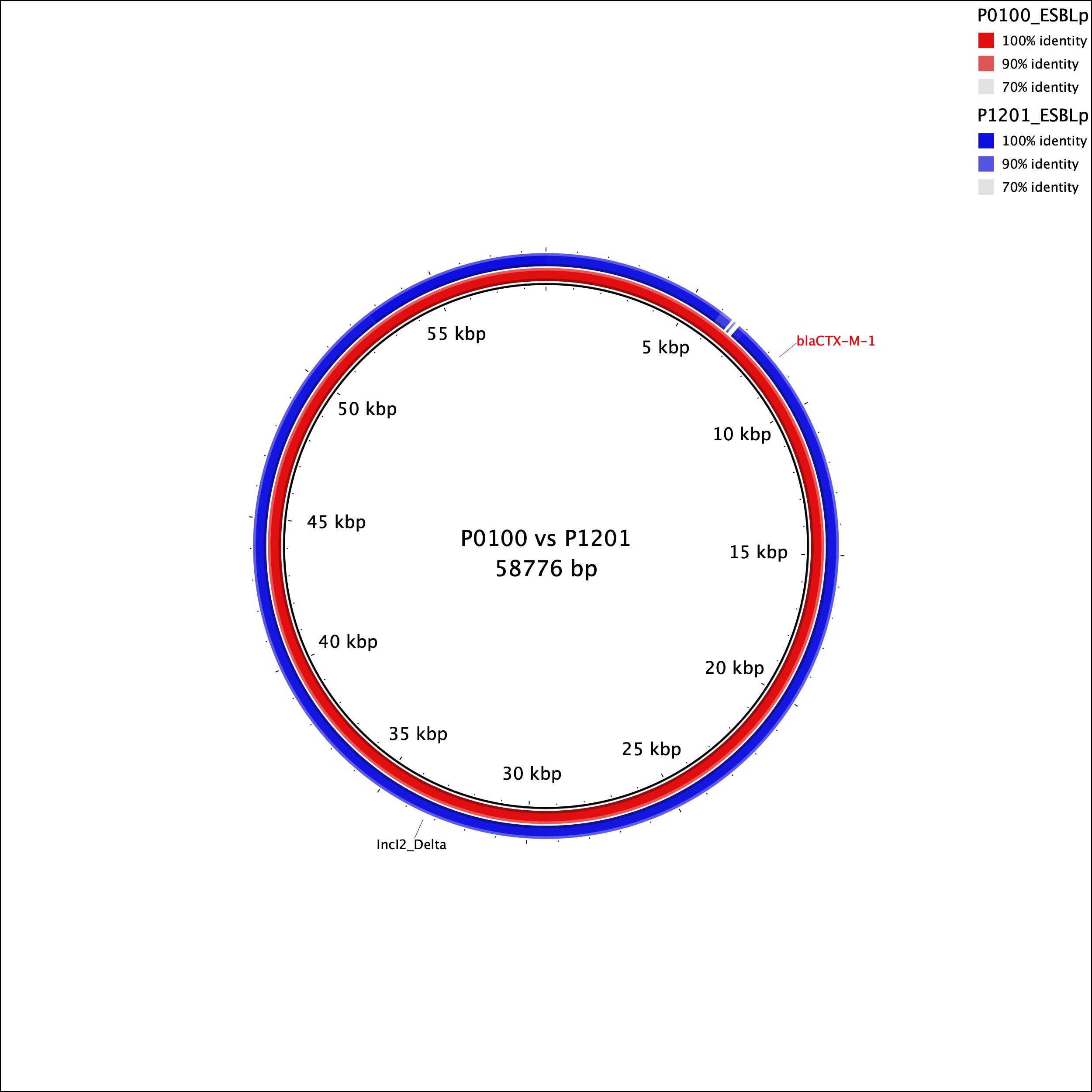

Supplement: Supplementary file 11 — Source Data [file 41467_2023_44285_MOESM11_ESM.zip › SourceDataFile/ESBLp_figures/Ecoli_ESBLp_BRIG_figures_allPacBio/P0100_ESBLp.fasta_comparison2.jpg]

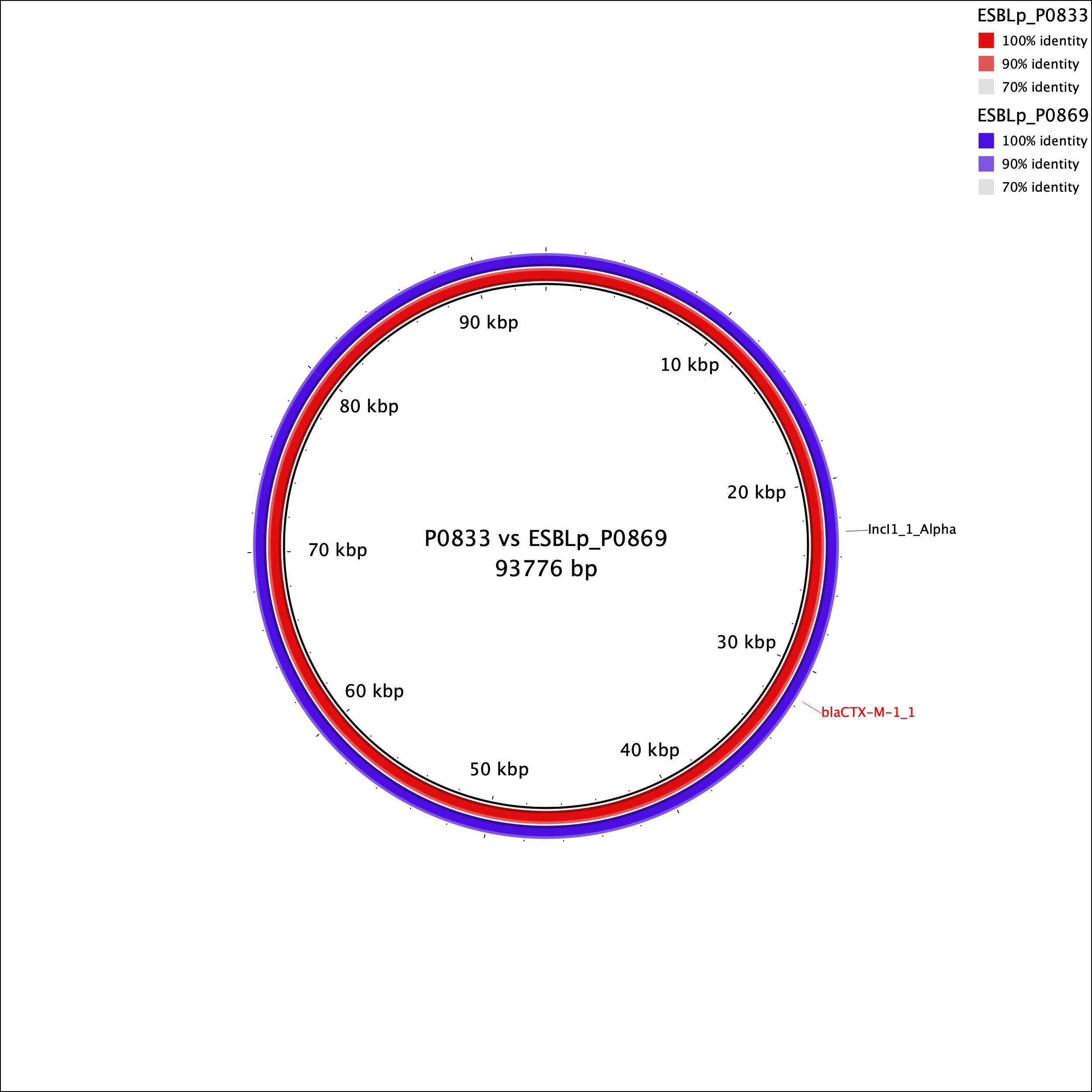

Supplement: Supplementary file 11 — Source Data [file 41467_2023_44285_MOESM11_ESM.zip › SourceDataFile/ESBLp_figures/Ecoli_ESBLp_BRIG_figures_allPacBio/P0833_ESBLp.fasta.jpg]

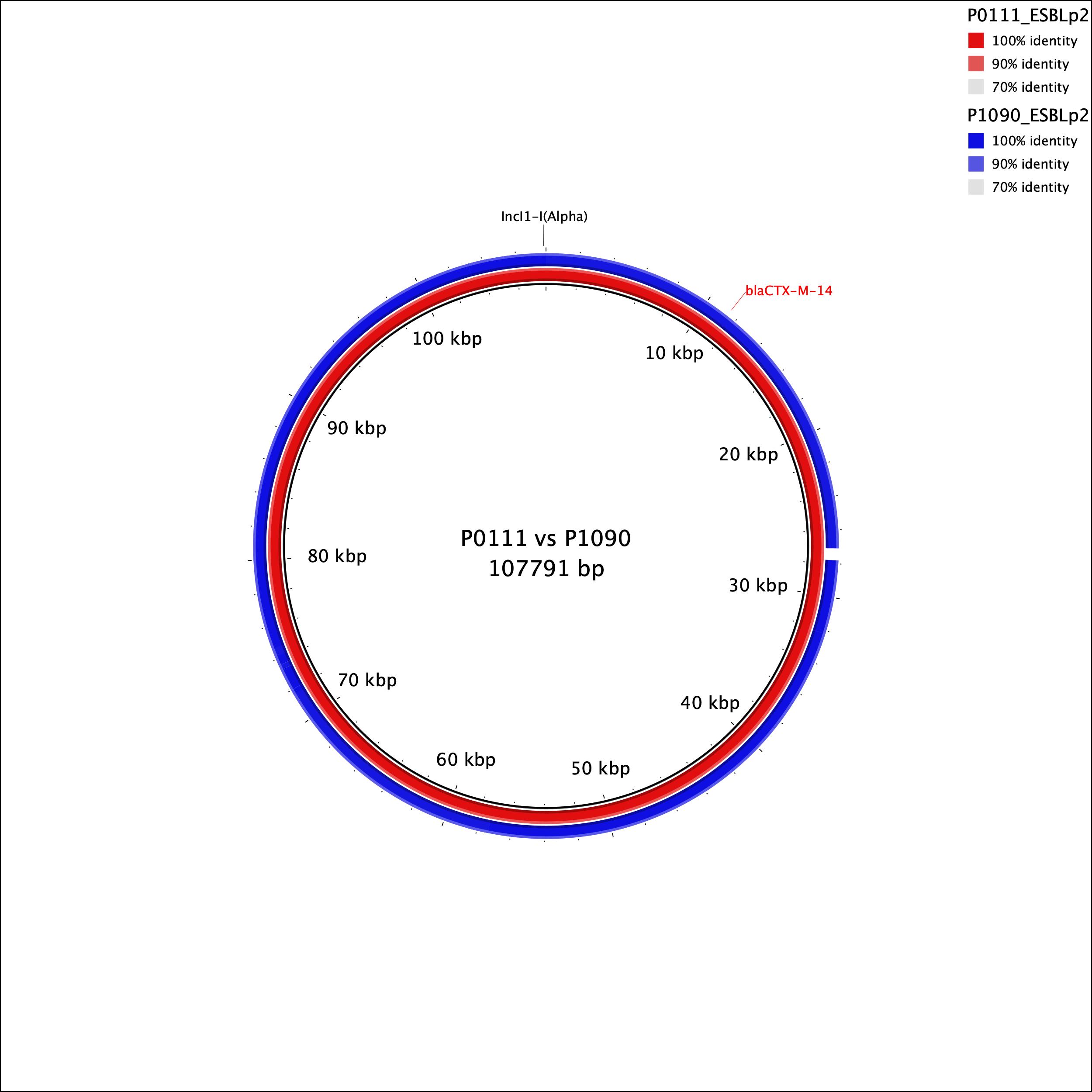

Supplement: Supplementary file 11 — Source Data [file 41467_2023_44285_MOESM11_ESM.zip › SourceDataFile/ESBLp_figures/Ecoli_ESBLp_BRIG_figures_allPacBio/P0111_ESBLp2.fasta.jpg]

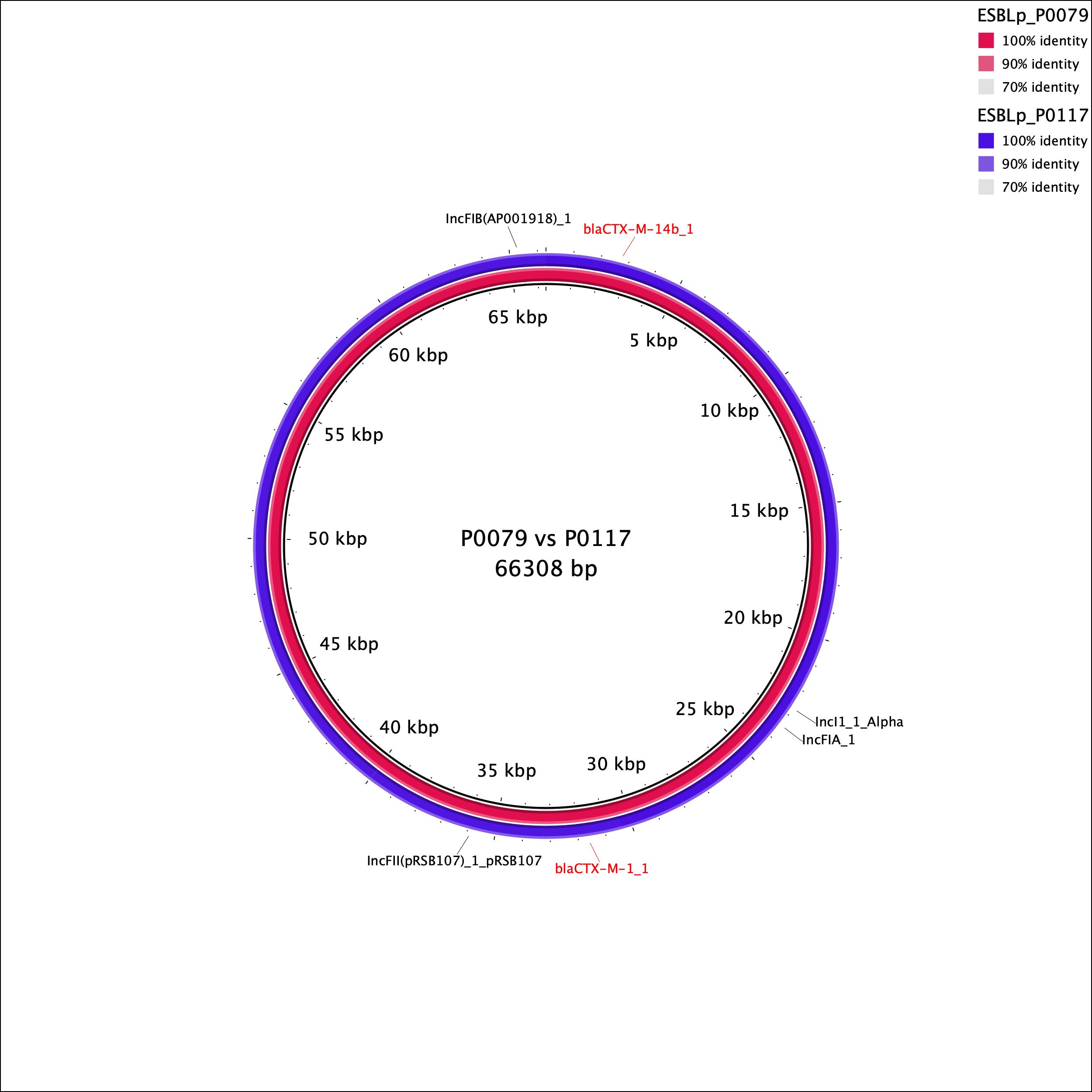

Supplement: Supplementary file 11 — Source Data [file 41467_2023_44285_MOESM11_ESM.zip › SourceDataFile/ESBLp_figures/Ecoli_ESBLp_BRIG_figures_allPacBio/P0079_ESBLp.fasta.jpg]

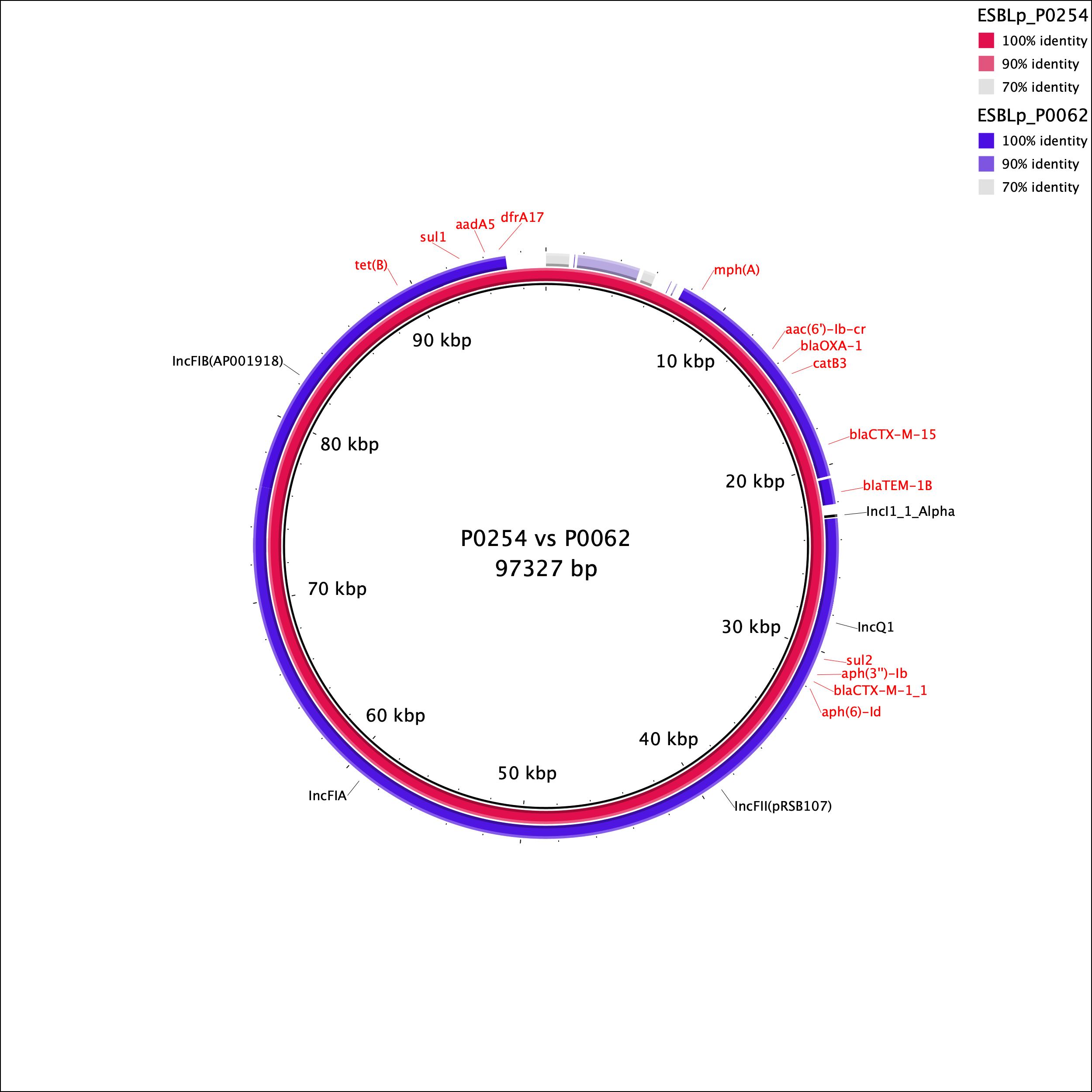

Supplement: Supplementary file 11 — Source Data [file 41467_2023_44285_MOESM11_ESM.zip › SourceDataFile/ESBLp_figures/Ecoli_ESBLp_BRIG_figures_allPacBio/P0254_ESBLp.fasta.jpg]

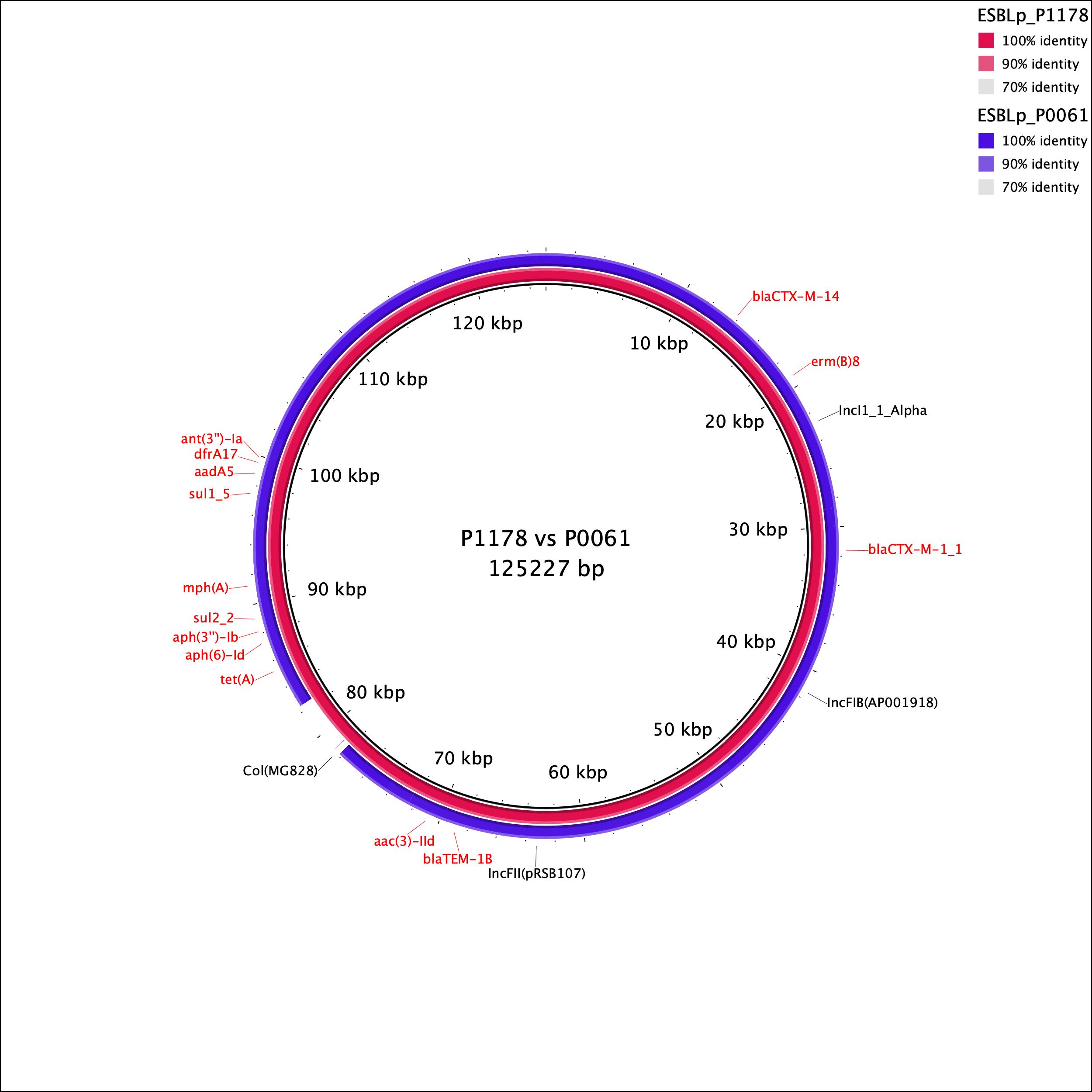

Supplement: Supplementary file 11 — Source Data [file 41467_2023_44285_MOESM11_ESM.zip › SourceDataFile/ESBLp_figures/Ecoli_ESBLp_BRIG_figures_allPacBio/P1178_ESBLp.fasta_comparison1.jpg]

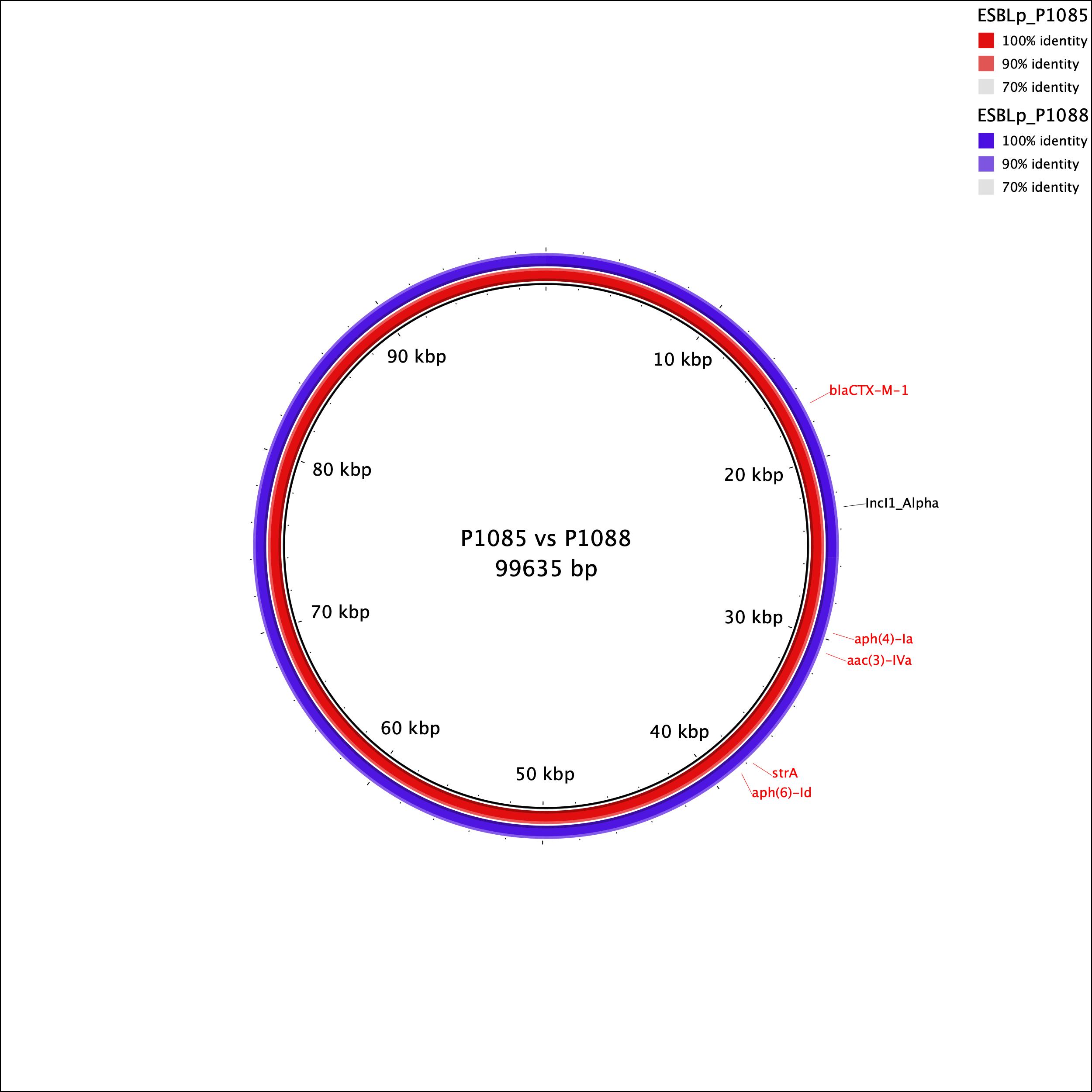

Supplement: Supplementary file 11 — Source Data [file 41467_2023_44285_MOESM11_ESM.zip › SourceDataFile/ESBLp_figures/Ecoli_ESBLp_BRIG_figures_allPacBio/P1085_ESBLp.fasta.jpg]

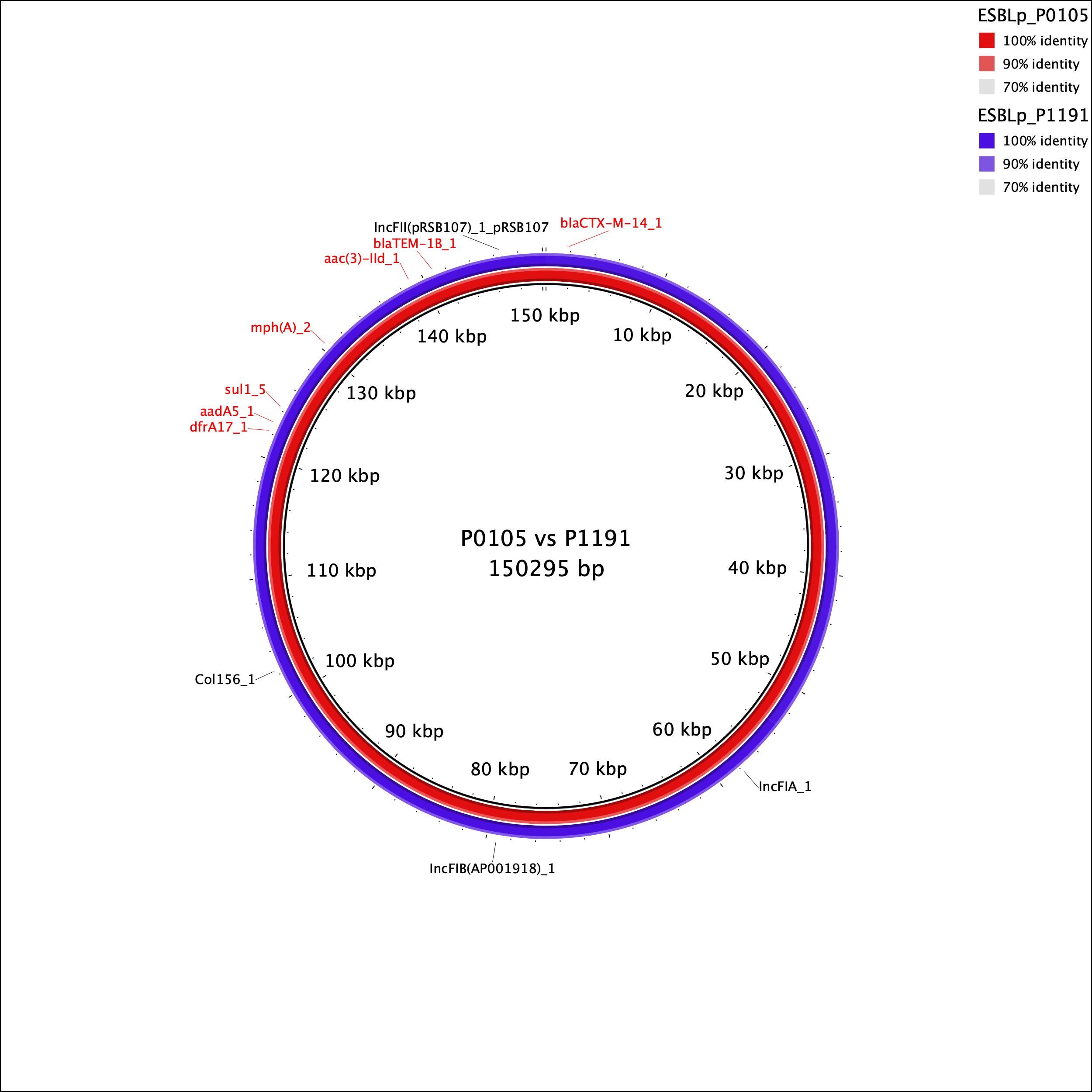

Supplement: Supplementary file 11 — Source Data [file 41467_2023_44285_MOESM11_ESM.zip › SourceDataFile/ESBLp_figures/Ecoli_ESBLp_BRIG_figures_allPacBio/P0105_ESBLp.fasta.jpg]

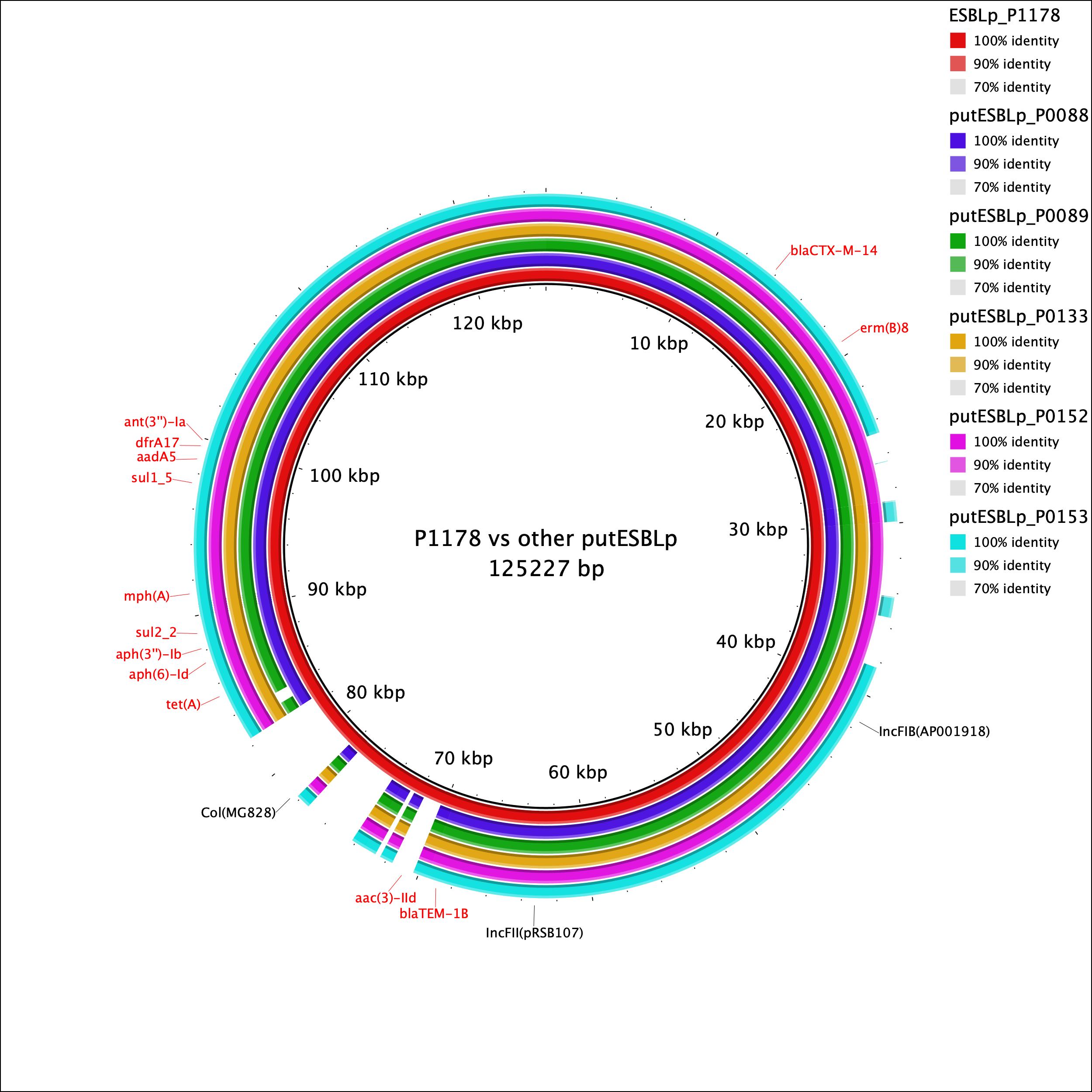

Supplement: Supplementary file 11 — Source Data [file 41467_2023_44285_MOESM11_ESM.zip › SourceDataFile/ESBLp_figures/Ecoli_ESBLp_BRIG_figures_allPacBio/P1178_ESBLp.fasta_comparison2.jpg]

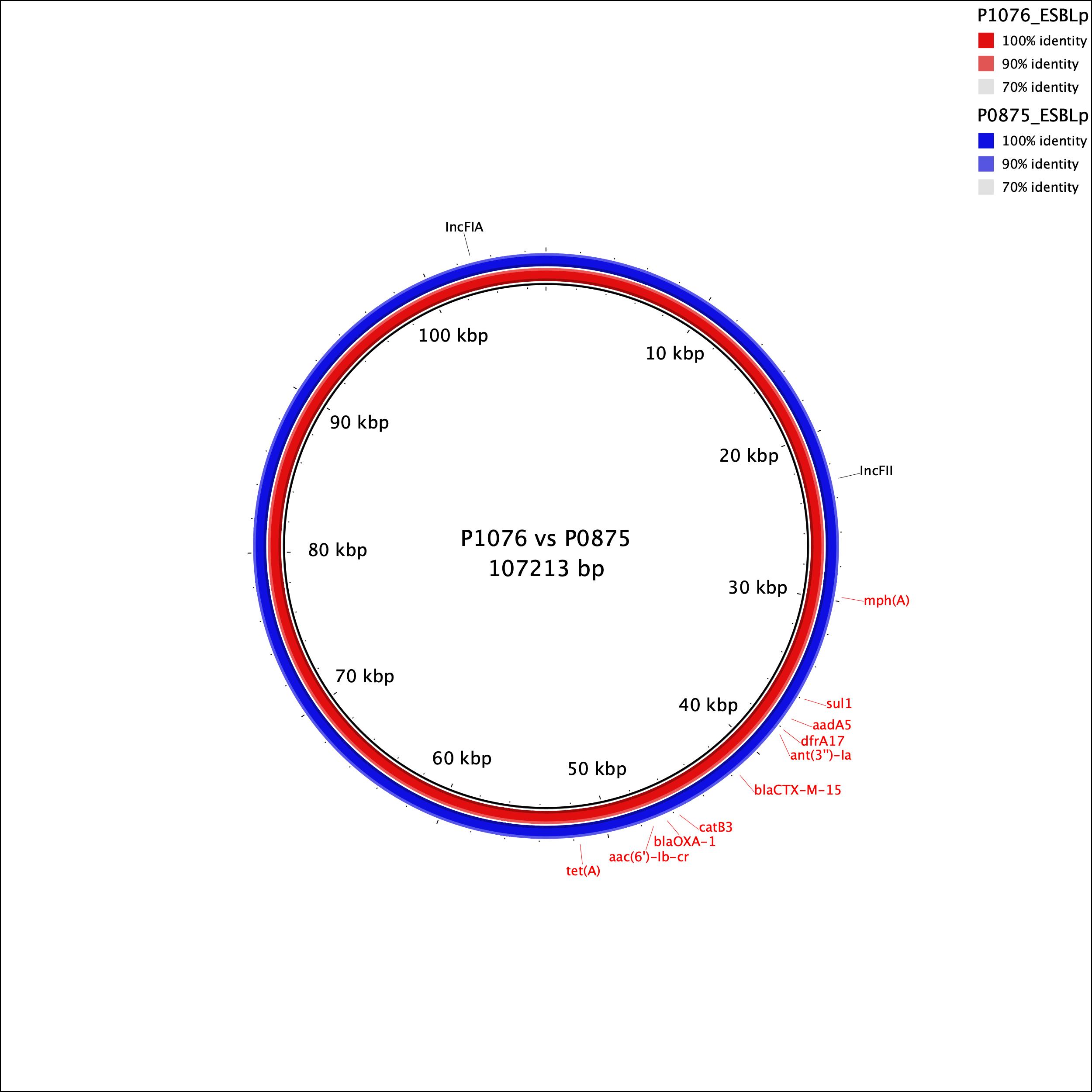

Supplement: Supplementary file 11 — Source Data [file 41467_2023_44285_MOESM11_ESM.zip › SourceDataFile/ESBLp_figures/Ecoli_ESBLp_BRIG_figures_allPacBio/P1076_ESBLp.fasta_comparison2.jpg]

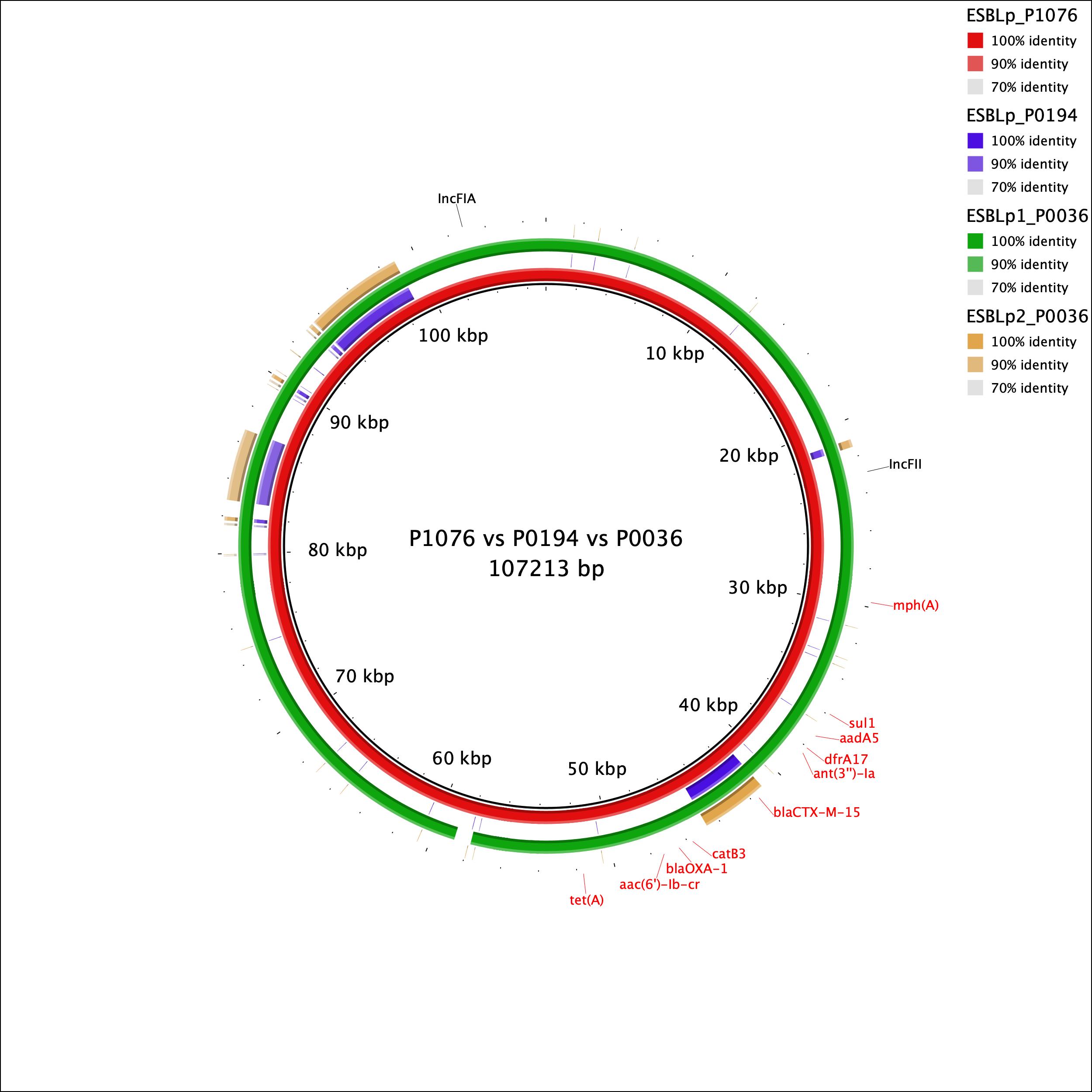

Supplement: Supplementary file 11 — Source Data [file 41467_2023_44285_MOESM11_ESM.zip › SourceDataFile/ESBLp_figures/Ecoli_ESBLp_BRIG_figures_allPacBio/P1076_ESBLp.fasta_comparison1.jpg]

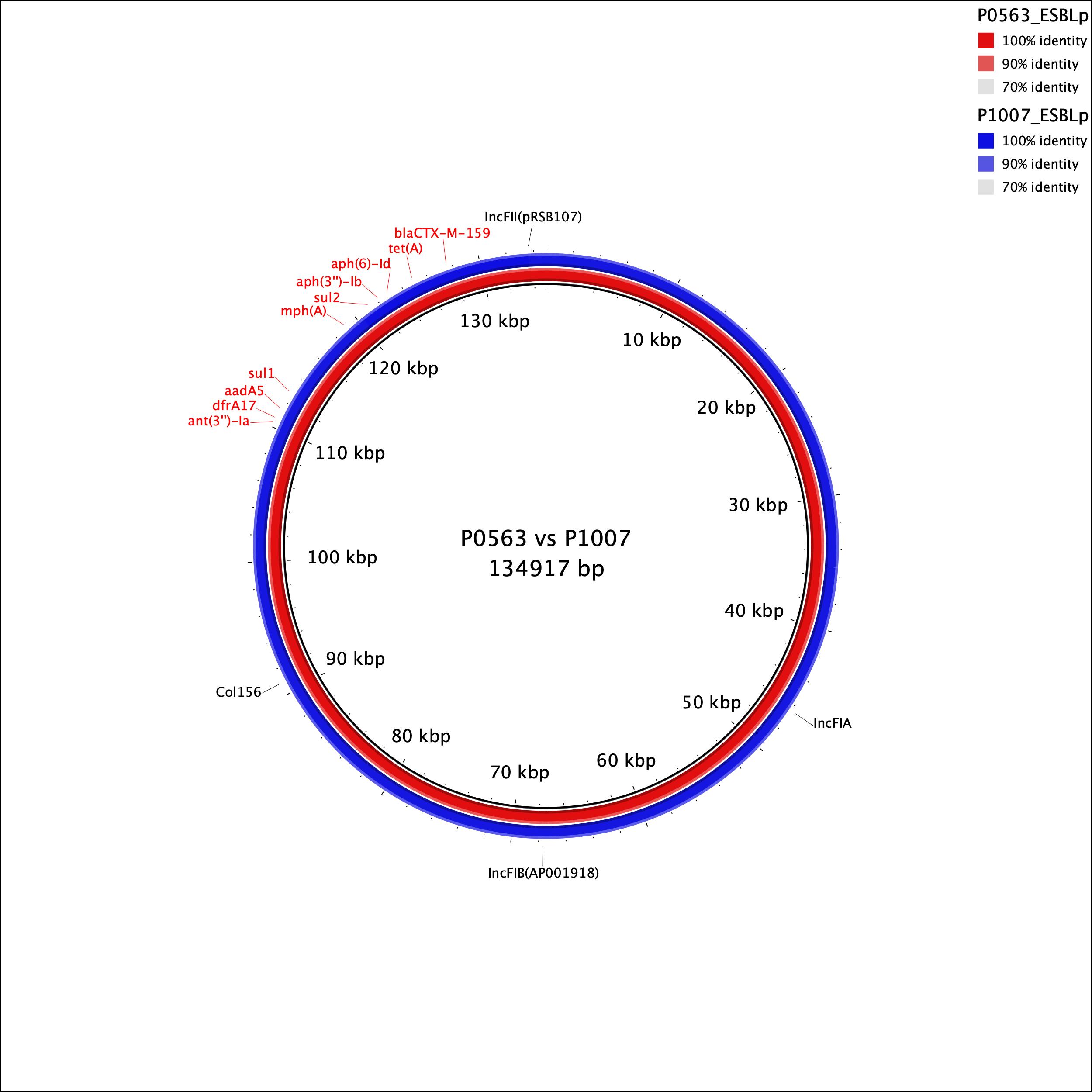

Supplement: Supplementary file 11 — Source Data [file 41467_2023_44285_MOESM11_ESM.zip › SourceDataFile/ESBLp_figures/Ecoli_ESBLp_BRIG_figures_allPacBio/P0563_ESBLp.fasta.jpg]

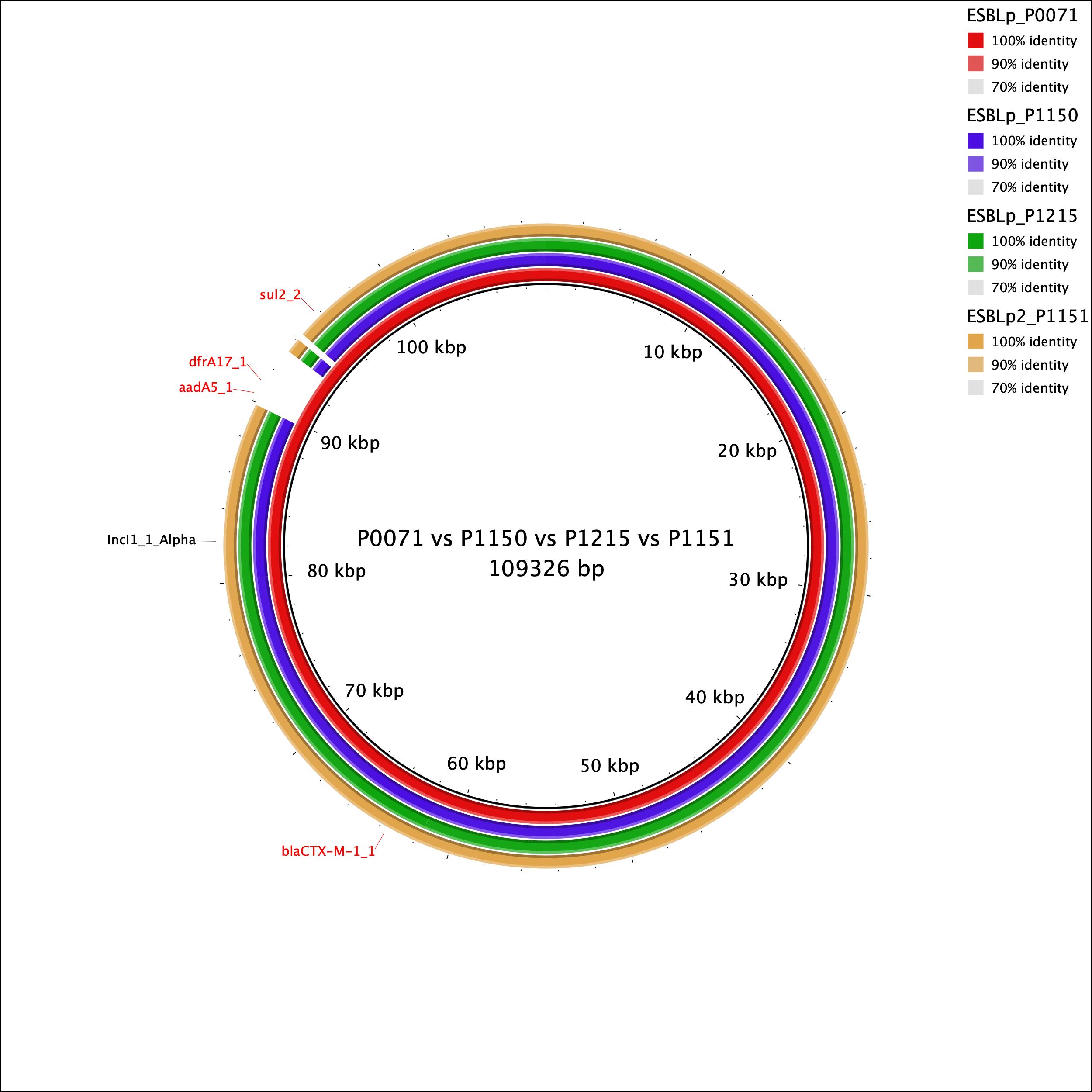

Supplement: Supplementary file 11 — Source Data [file 41467_2023_44285_MOESM11_ESM.zip › SourceDataFile/ESBLp_figures/Ecoli_ESBLp_BRIG_figures_allPacBio/P0071_ESBLp.fasta_comparison1.jpg]

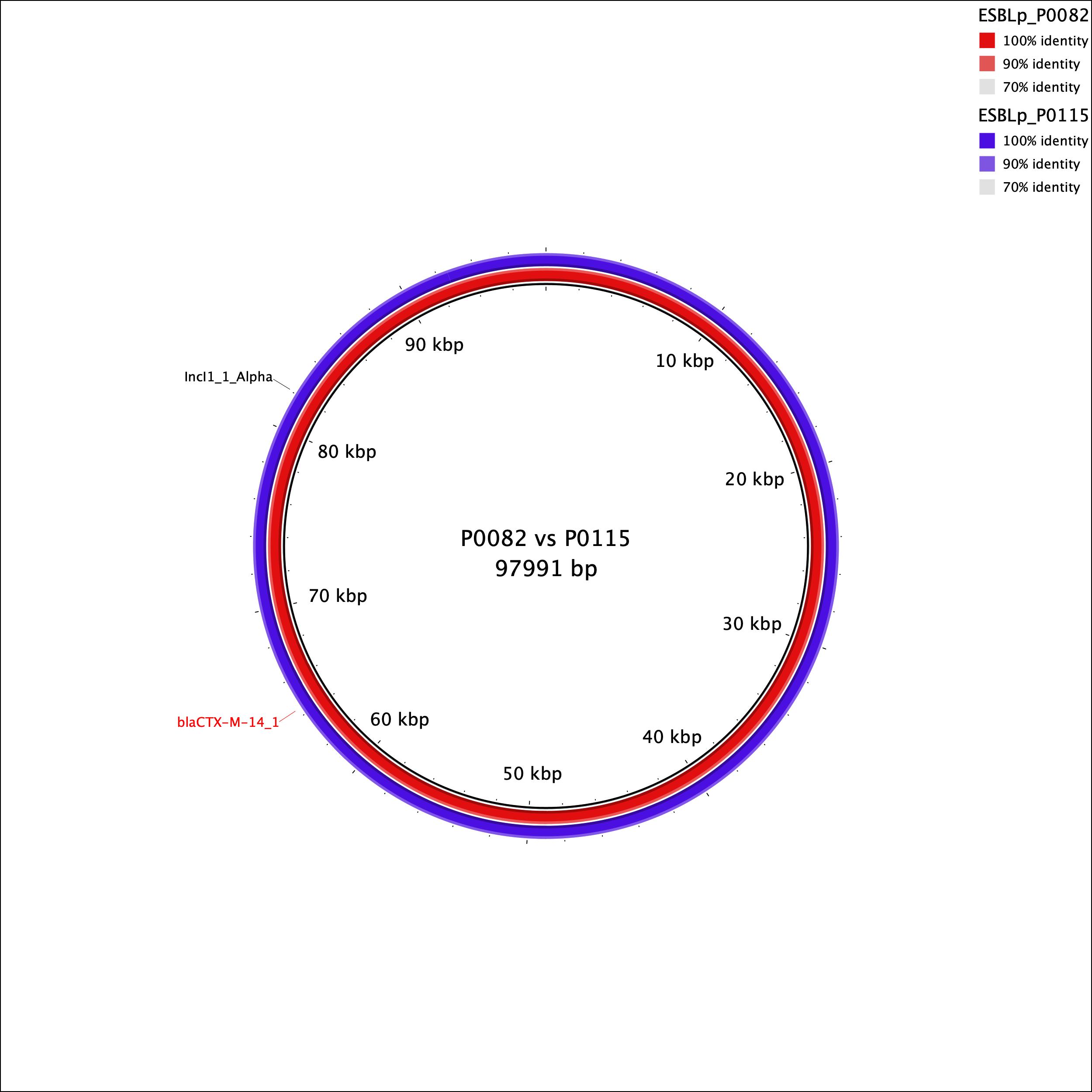

Supplement: Supplementary file 11 — Source Data [file 41467_2023_44285_MOESM11_ESM.zip › SourceDataFile/ESBLp_figures/Ecoli_ESBLp_BRIG_figures_allPacBio/P0082_ESBLp.fasta.jpg]

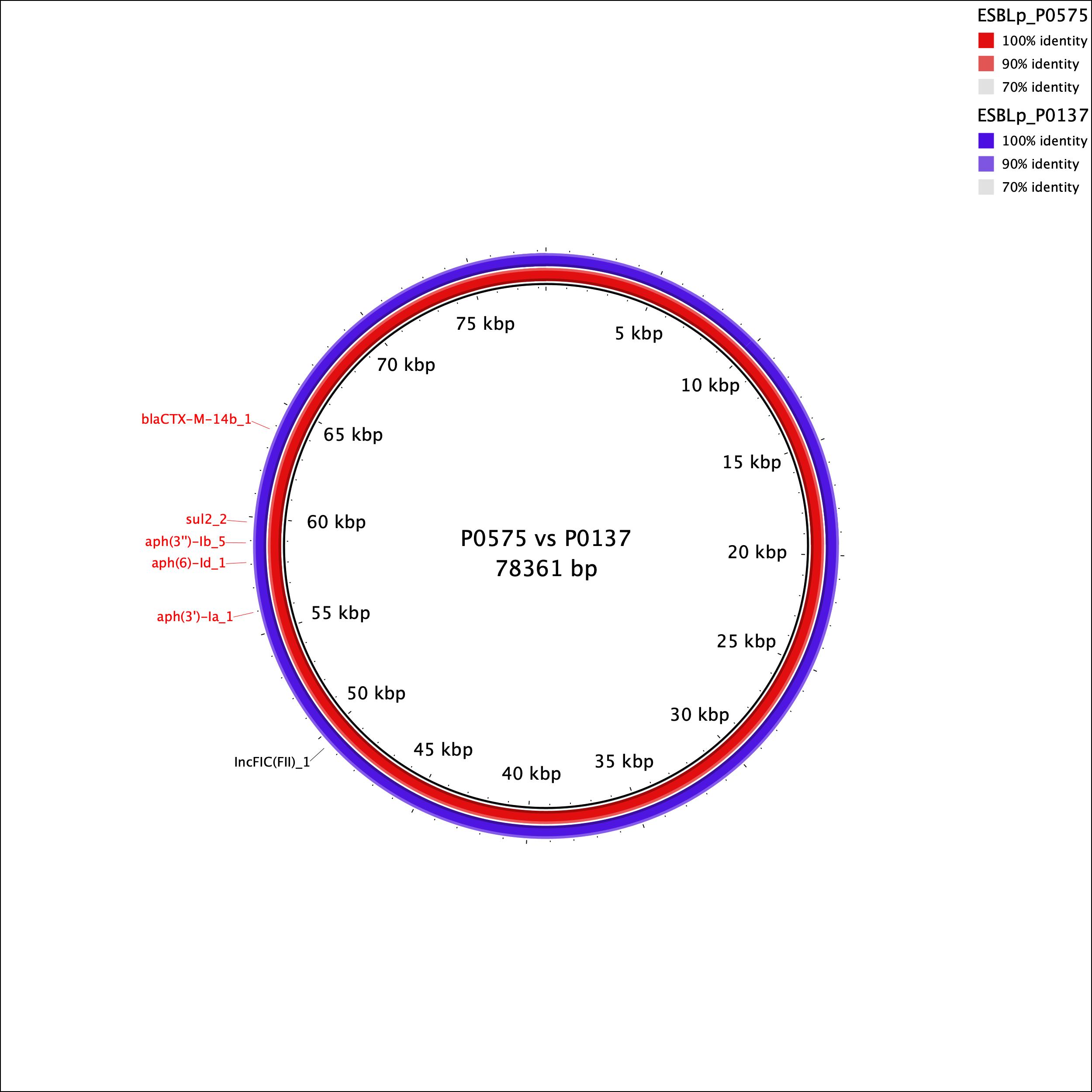

Supplement: Supplementary file 11 — Source Data [file 41467_2023_44285_MOESM11_ESM.zip › SourceDataFile/ESBLp_figures/Ecoli_ESBLp_BRIG_figures_allPacBio/P0575_ESBLp.fasta.jpg]

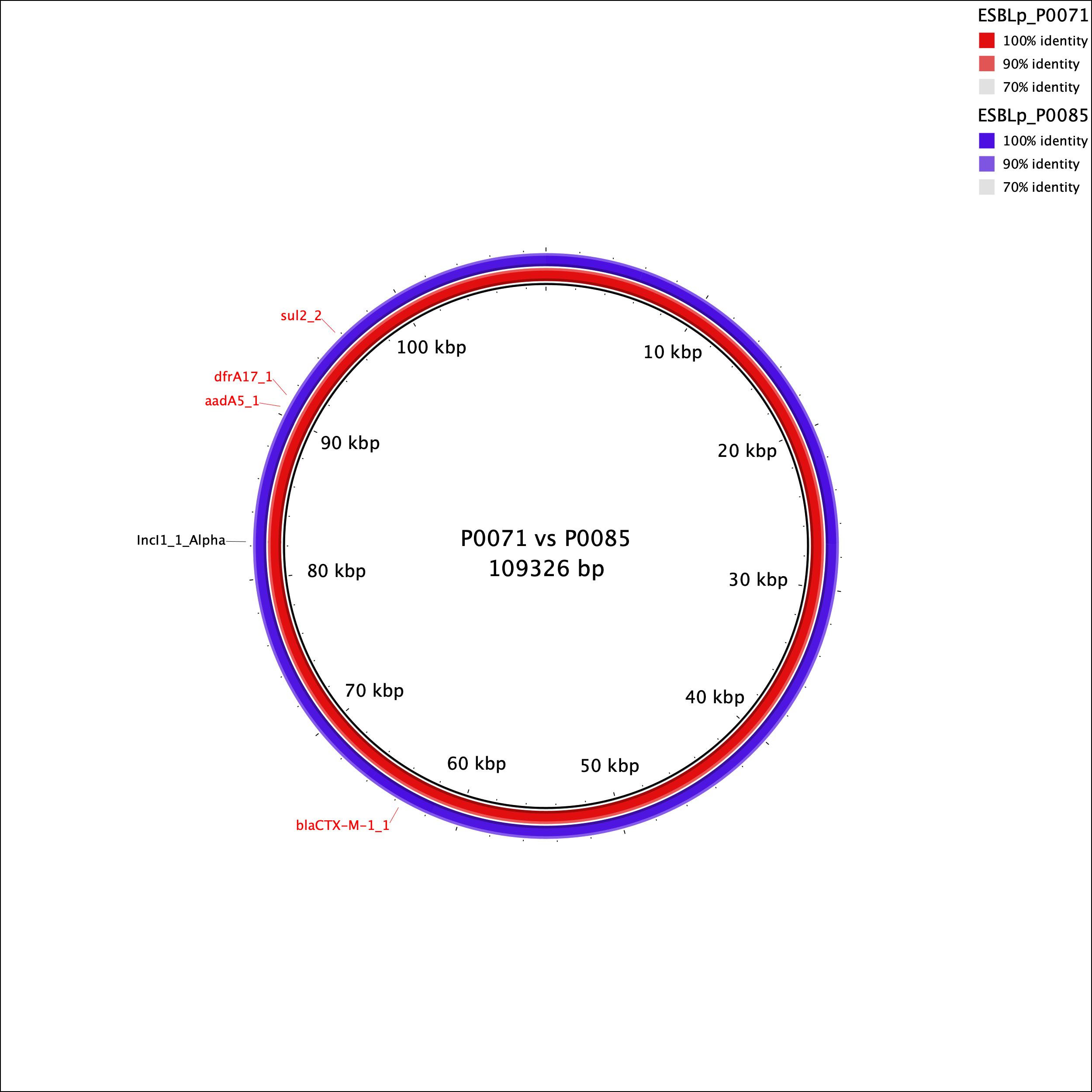

Supplement: Supplementary file 11 — Source Data [file 41467_2023_44285_MOESM11_ESM.zip › SourceDataFile/ESBLp_figures/Ecoli_ESBLp_BRIG_figures_allPacBio/P0071_ESBLp.fasta_comparison2.jpg]

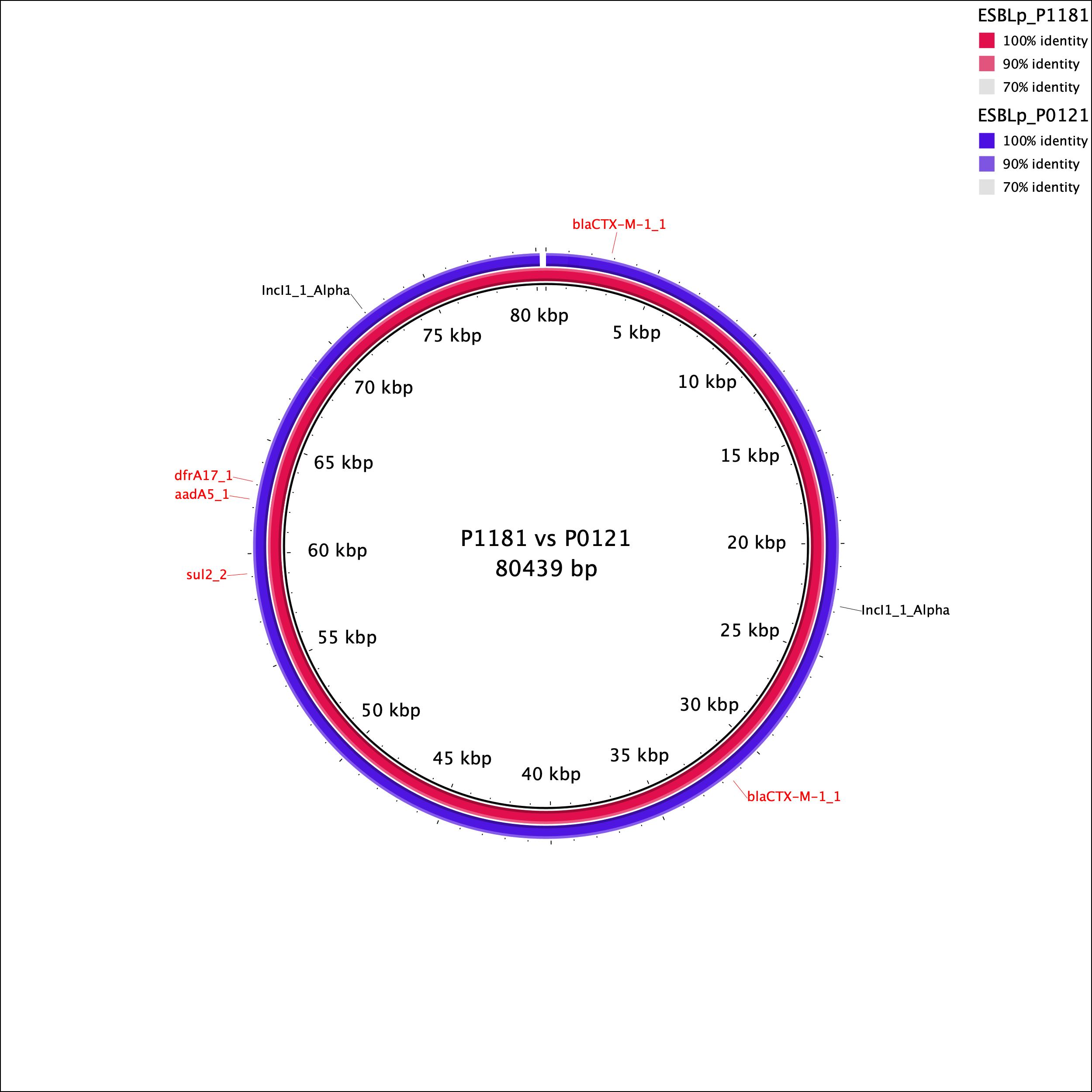

Supplement: Supplementary file 11 — Source Data [file 41467_2023_44285_MOESM11_ESM.zip › SourceDataFile/ESBLp_figures/Ecoli_ESBLp_BRIG_figures_allPacBio/P1181_putESBLp.fasta.jpg]

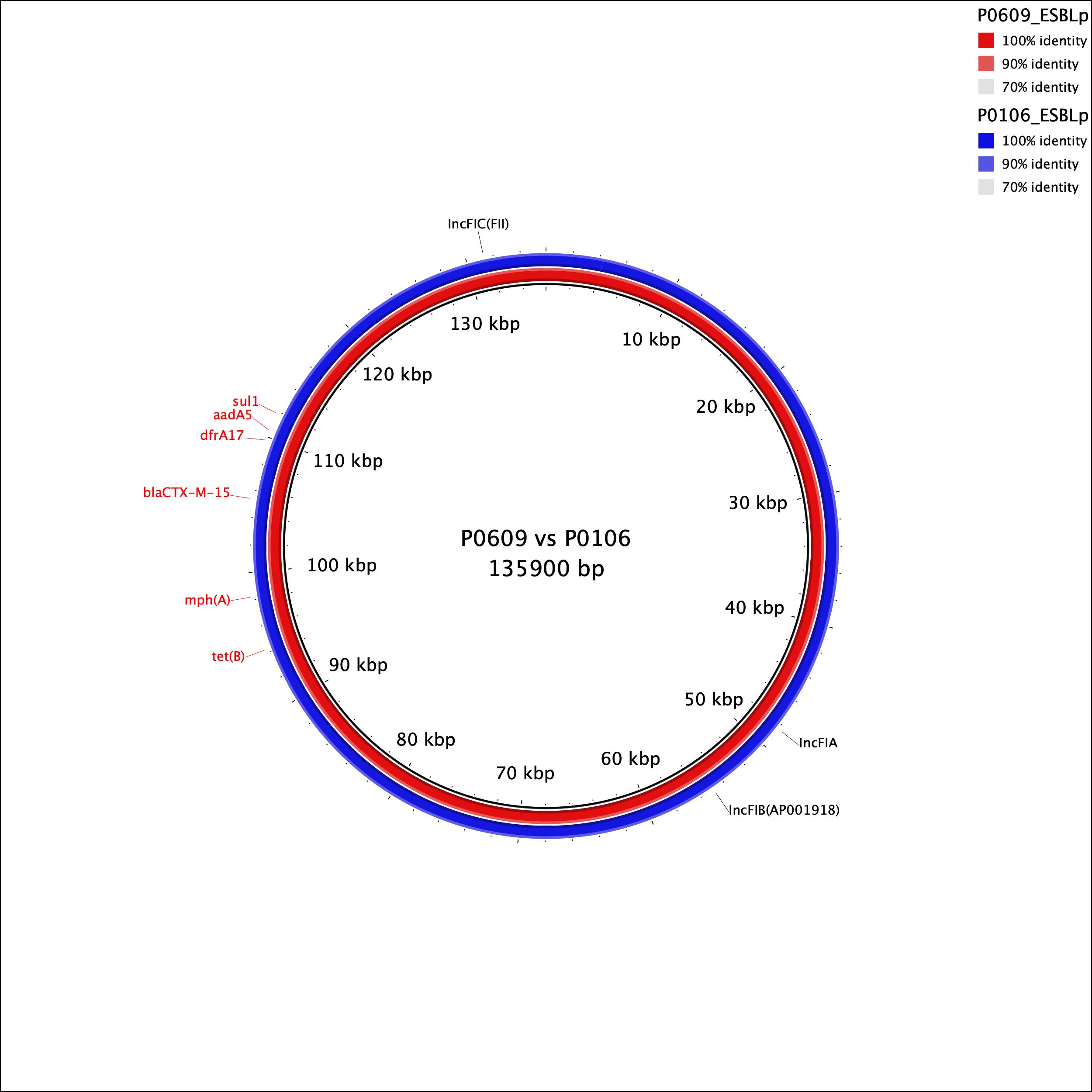

Supplement: Supplementary file 11 — Source Data [file 41467_2023_44285_MOESM11_ESM.zip › SourceDataFile/ESBLp_figures/Ecoli_ESBLp_BRIG_figures_allPacBio/P0609_ESBLp.fasta.jpg]

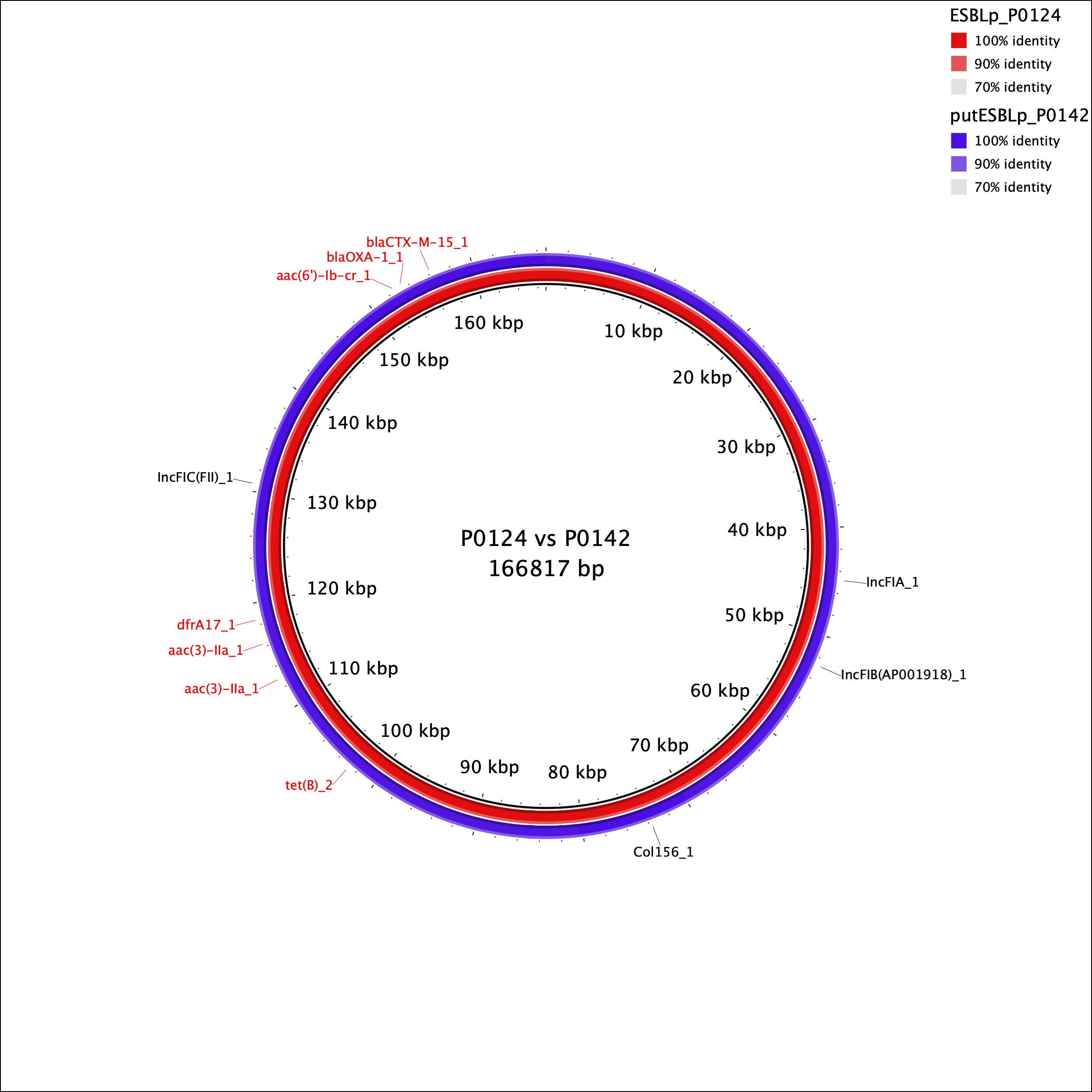

Supplement: Supplementary file 11 — Source Data [file 41467_2023_44285_MOESM11_ESM.zip › SourceDataFile/ESBLp_figures/Ecoli_ESBLp_BRIG_figures_allPacBio/P0124_ESBLp.fasta.jpg]

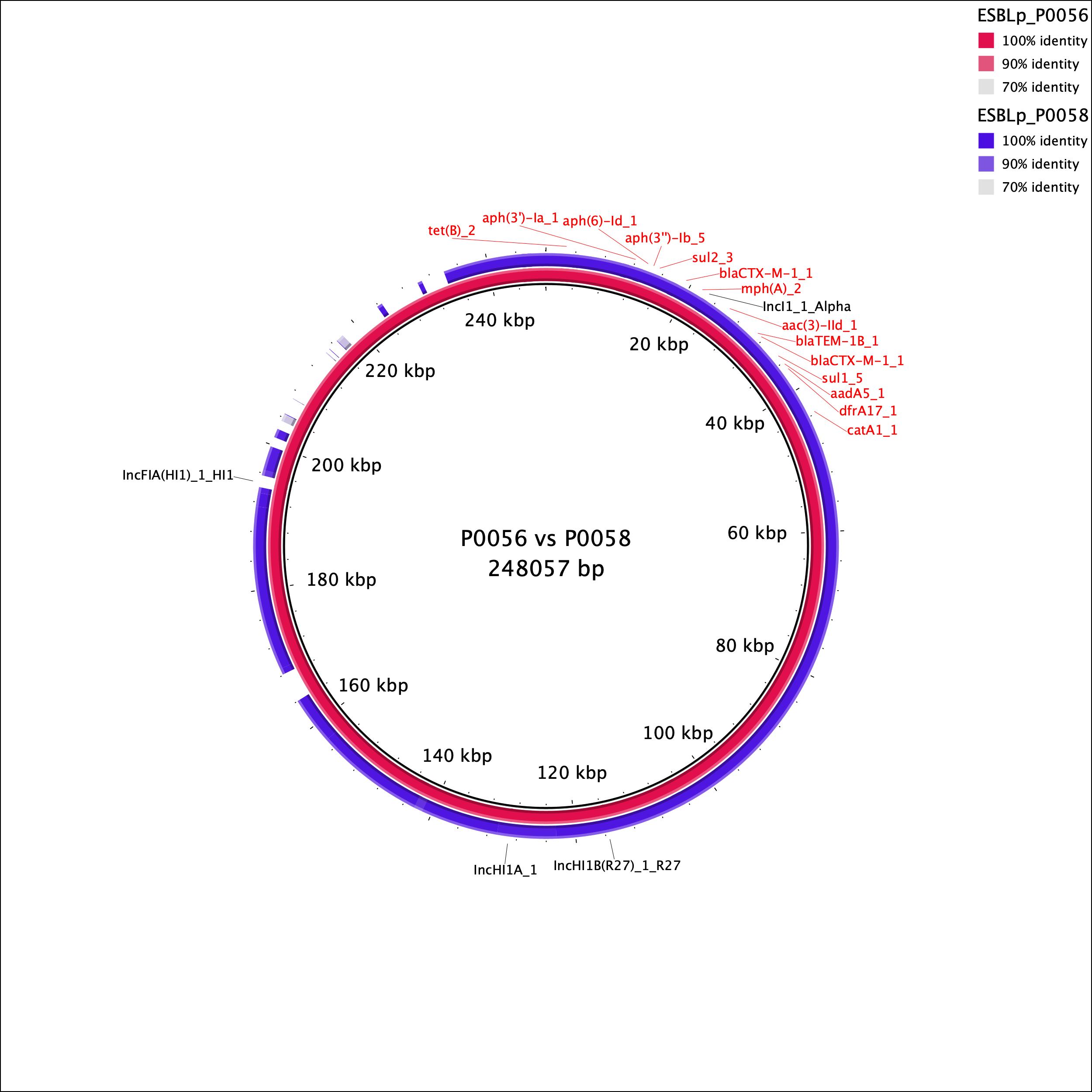

Supplement: Supplementary file 11 — Source Data [file 41467_2023_44285_MOESM11_ESM.zip › SourceDataFile/ESBLp_figures/Ecoli_ESBLp_BRIG_figures_allPacBio/P0056_ESBLp.fasta.jpg]

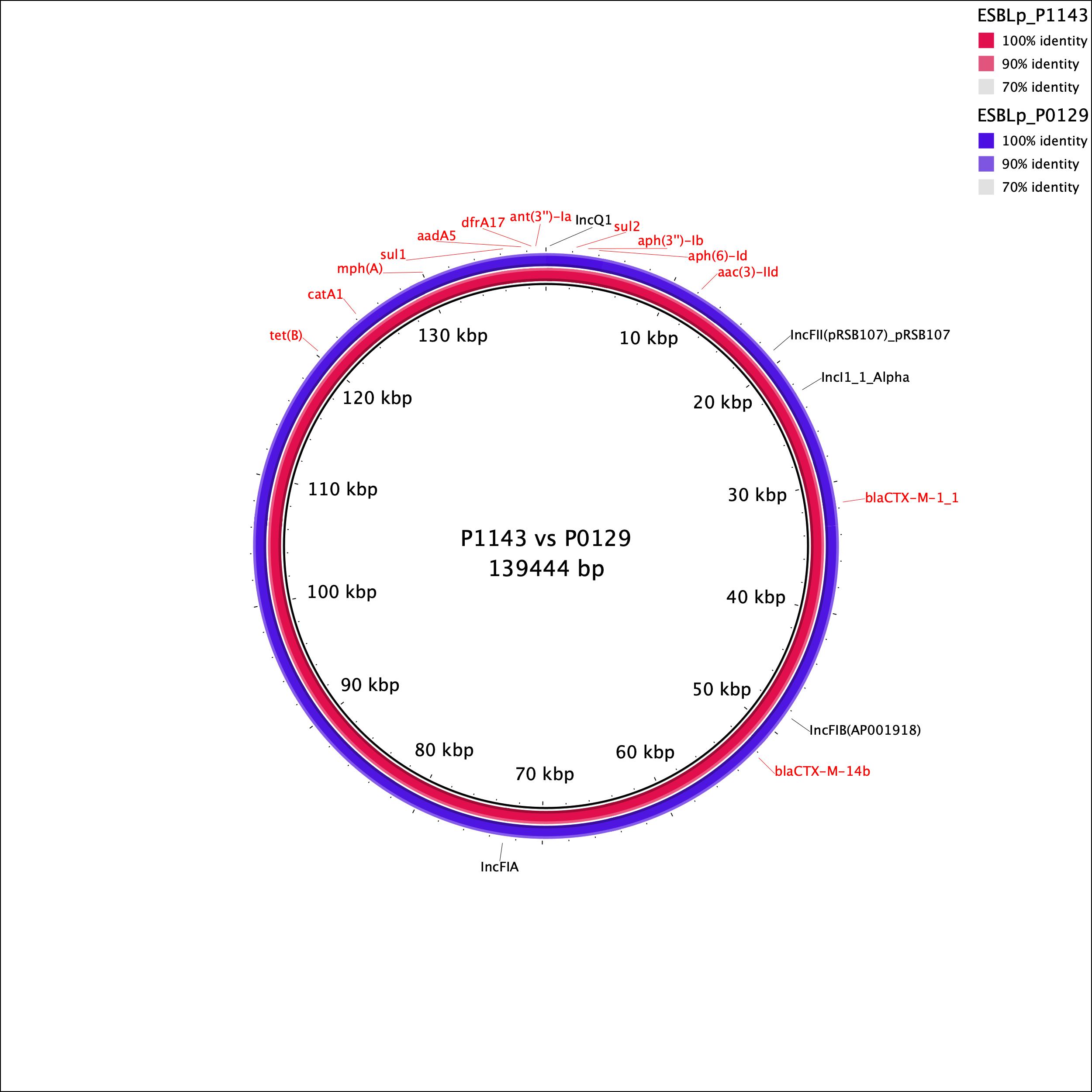

Supplement: Supplementary file 11 — Source Data [file 41467_2023_44285_MOESM11_ESM.zip › SourceDataFile/ESBLp_figures/Ecoli_ESBLp_BRIG_figures_allPacBio/P1143_ESBLp.fasta.jpg]

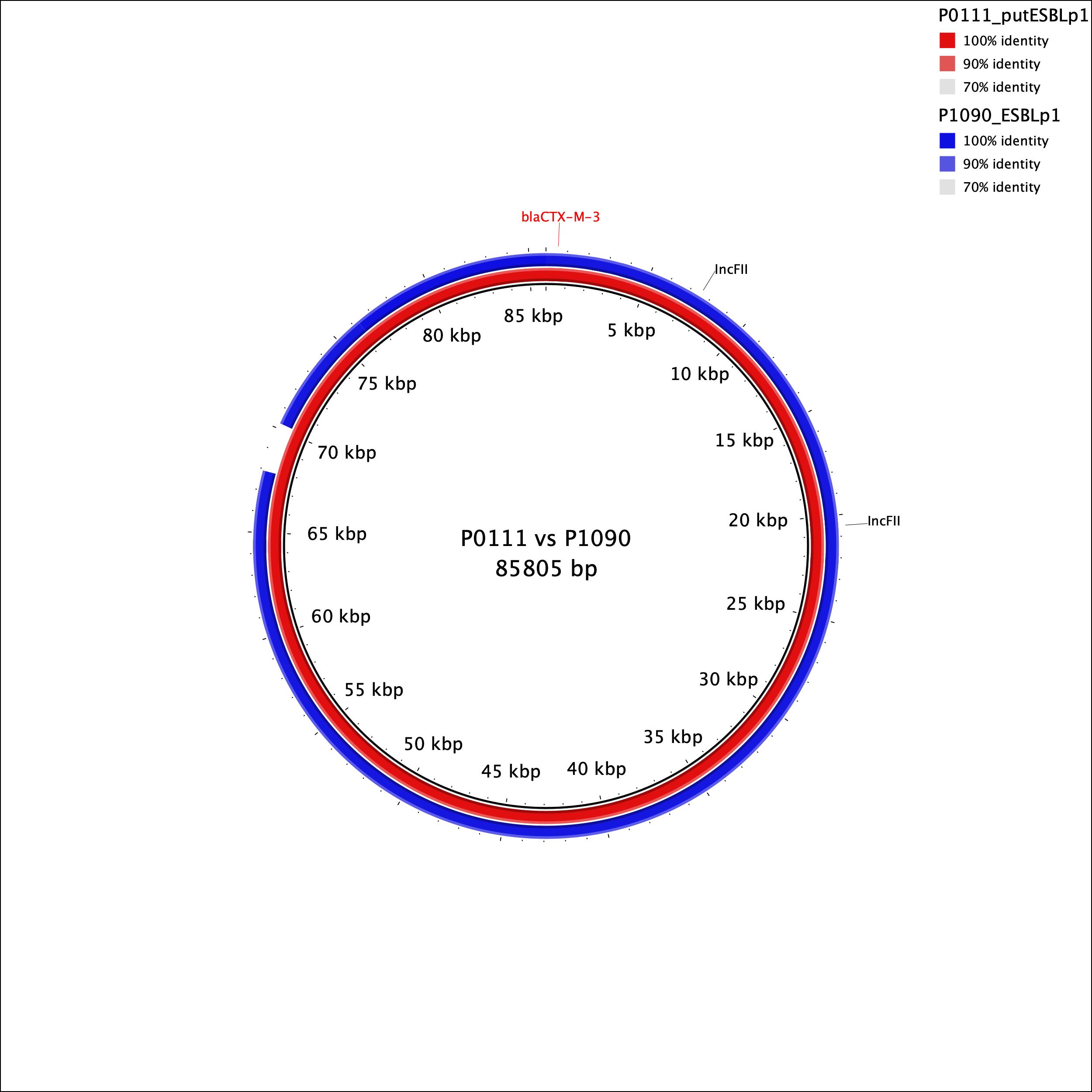

Supplement: Supplementary file 11 — Source Data [file 41467_2023_44285_MOESM11_ESM.zip › SourceDataFile/ESBLp_figures/Ecoli_ESBLp_BRIG_figures_allPacBio/P0111_putESBLp1_unic.fasta.jpg]

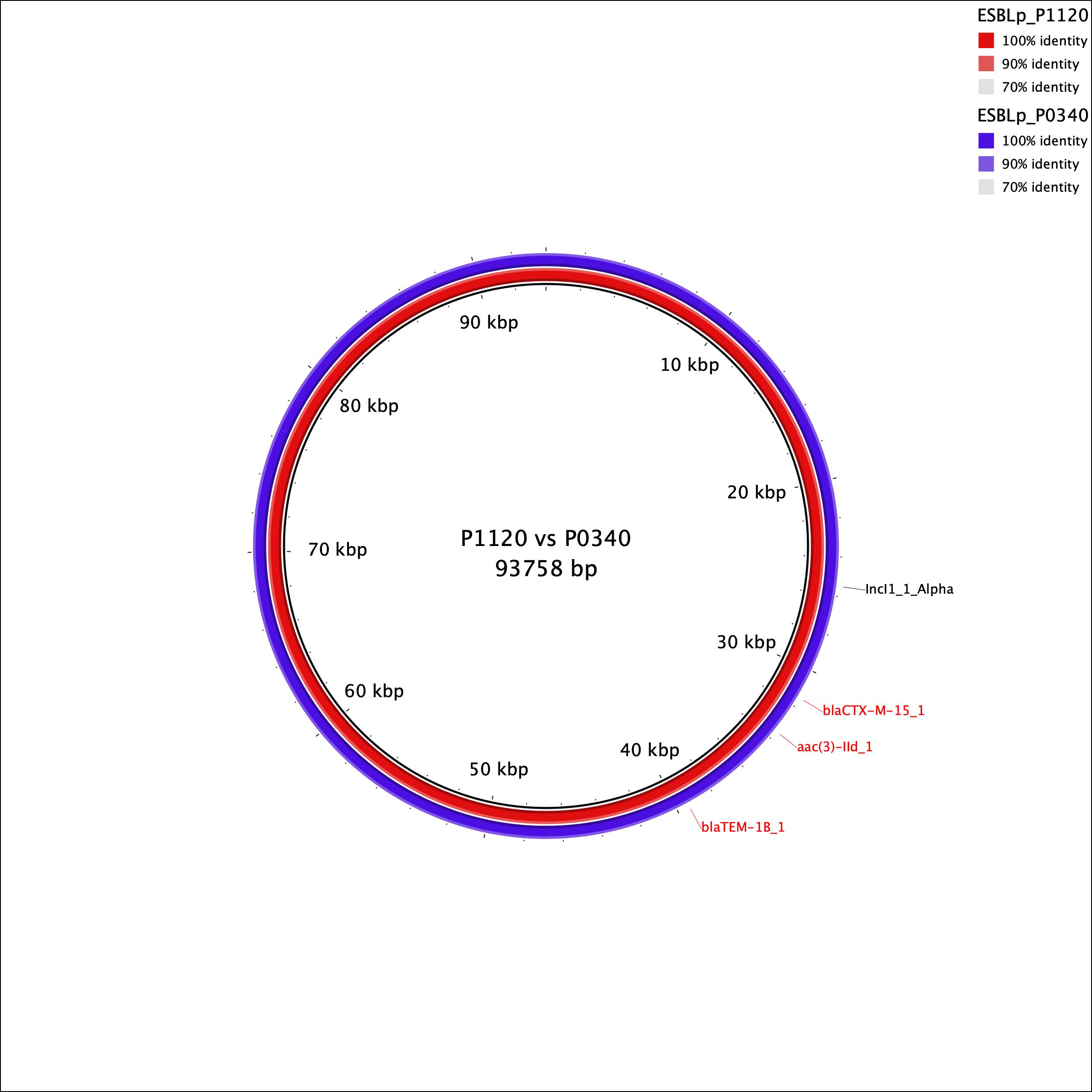

Supplement: Supplementary file 11 — Source Data [file 41467_2023_44285_MOESM11_ESM.zip › SourceDataFile/ESBLp_figures/Ecoli_ESBLp_BRIG_figures_allPacBio/P1120_ESBLp.fasta.jpg]

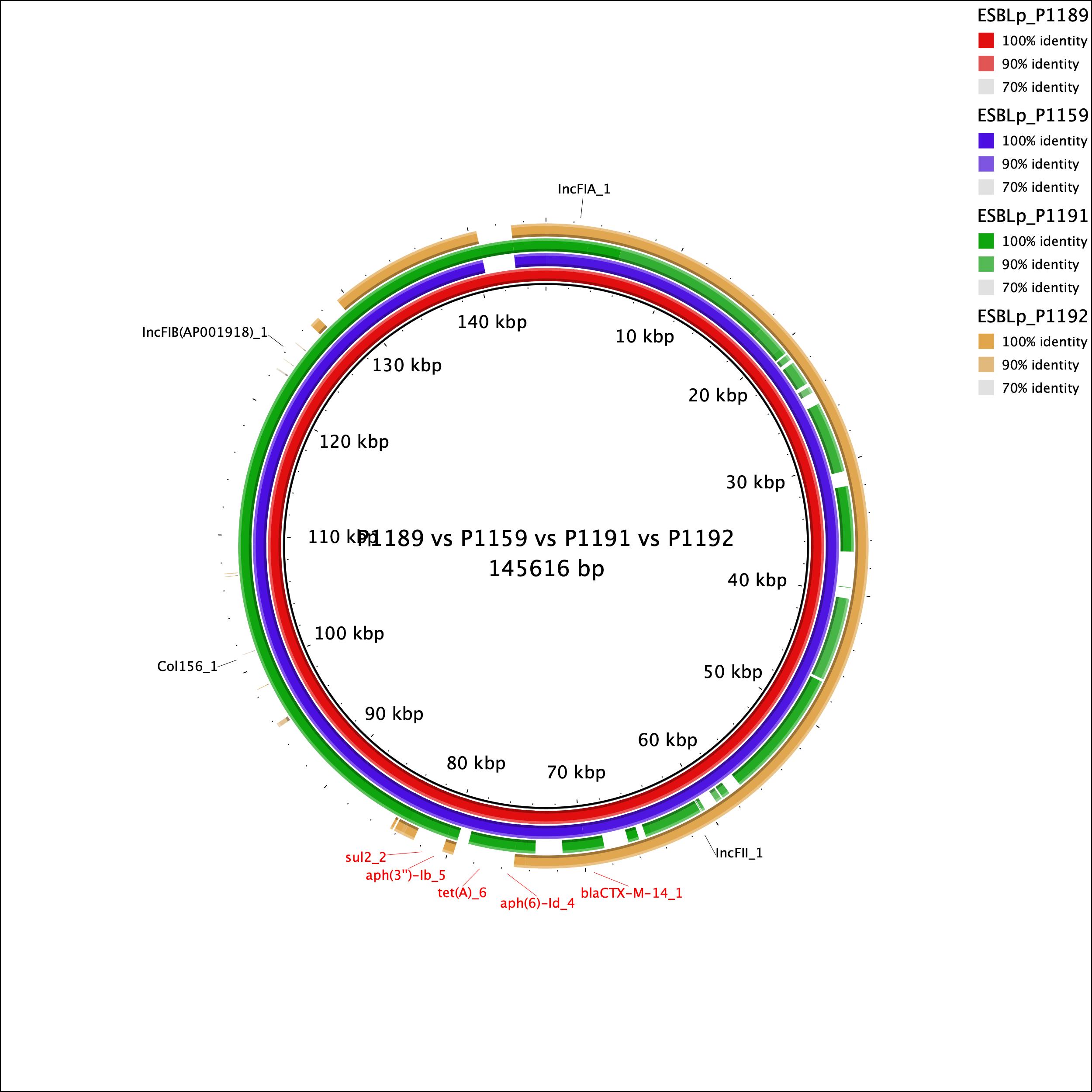

Supplement: Supplementary file 11 — Source Data [file 41467_2023_44285_MOESM11_ESM.zip › SourceDataFile/ESBLp_figures/Ecoli_ESBLp_BRIG_figures_allPacBio/P1189_ESBLp.fasta.jpg]

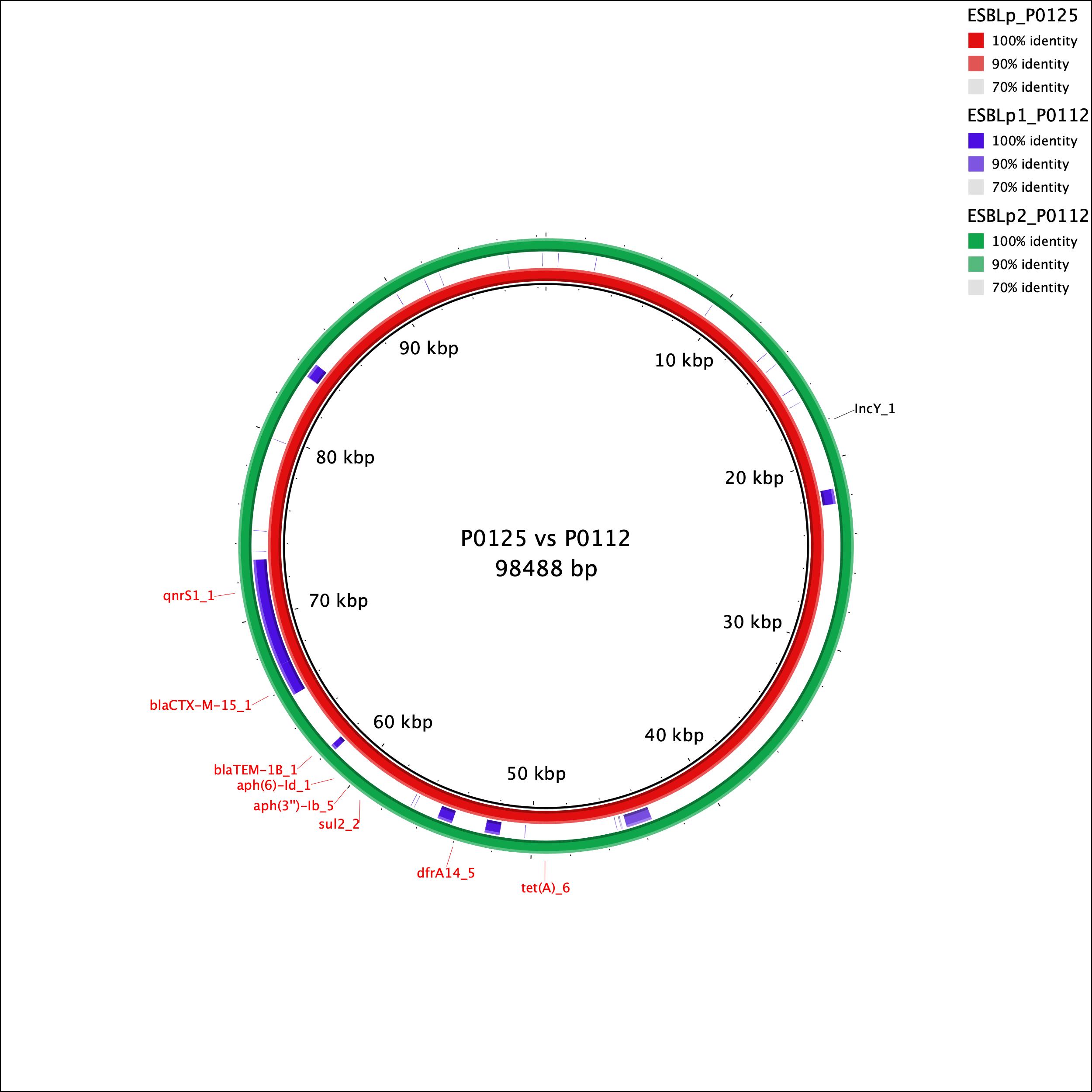

Supplement: Supplementary file 11 — Source Data [file 41467_2023_44285_MOESM11_ESM.zip › SourceDataFile/ESBLp_figures/Ecoli_ESBLp_BRIG_figures_allPacBio/P0125_ESBLp.fasta.jpg]

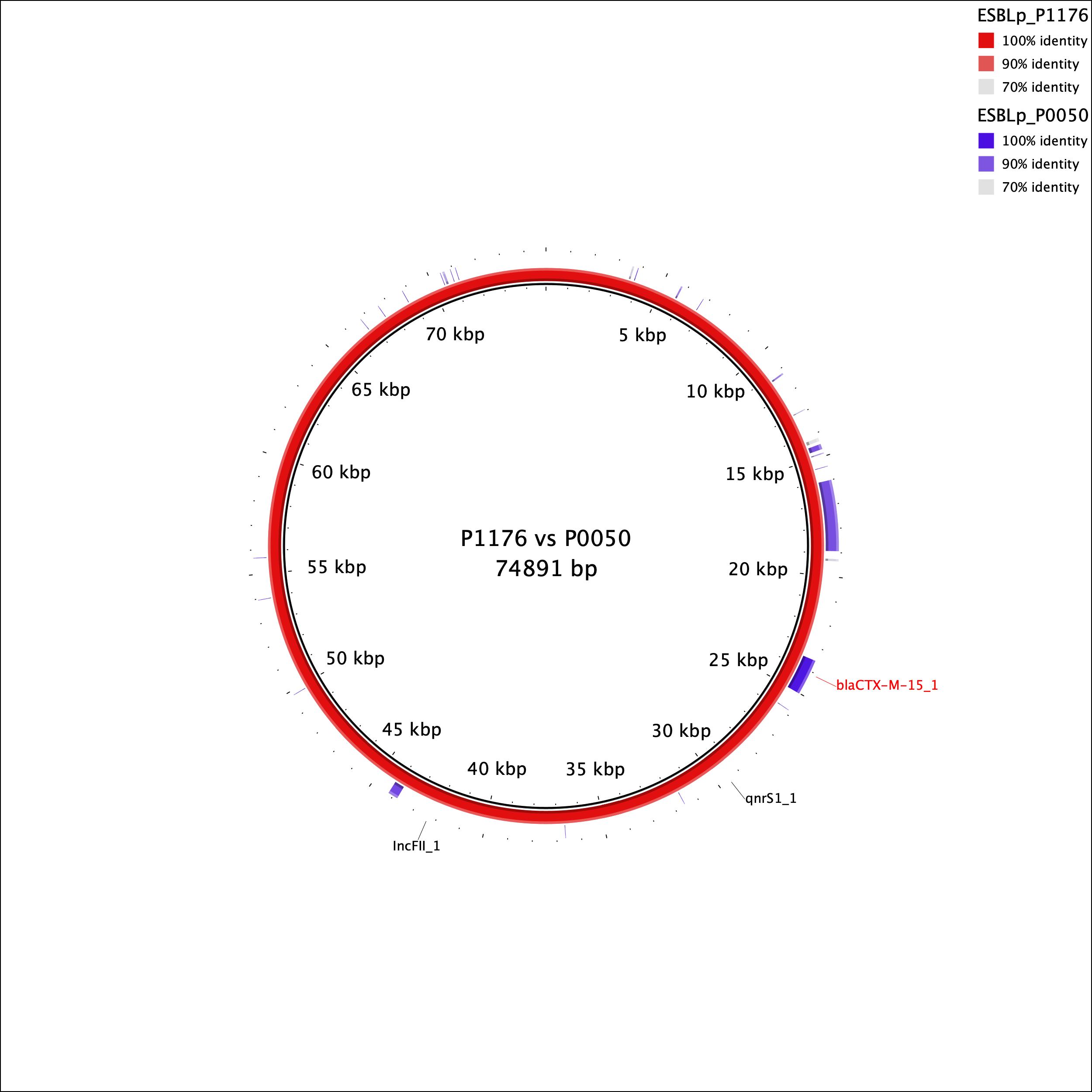

Supplement: Supplementary file 11 — Source Data [file 41467_2023_44285_MOESM11_ESM.zip › SourceDataFile/ESBLp_figures/Ecoli_ESBLp_BRIG_figures_allPacBio/P1176_ESBLp.fasta.jpg]

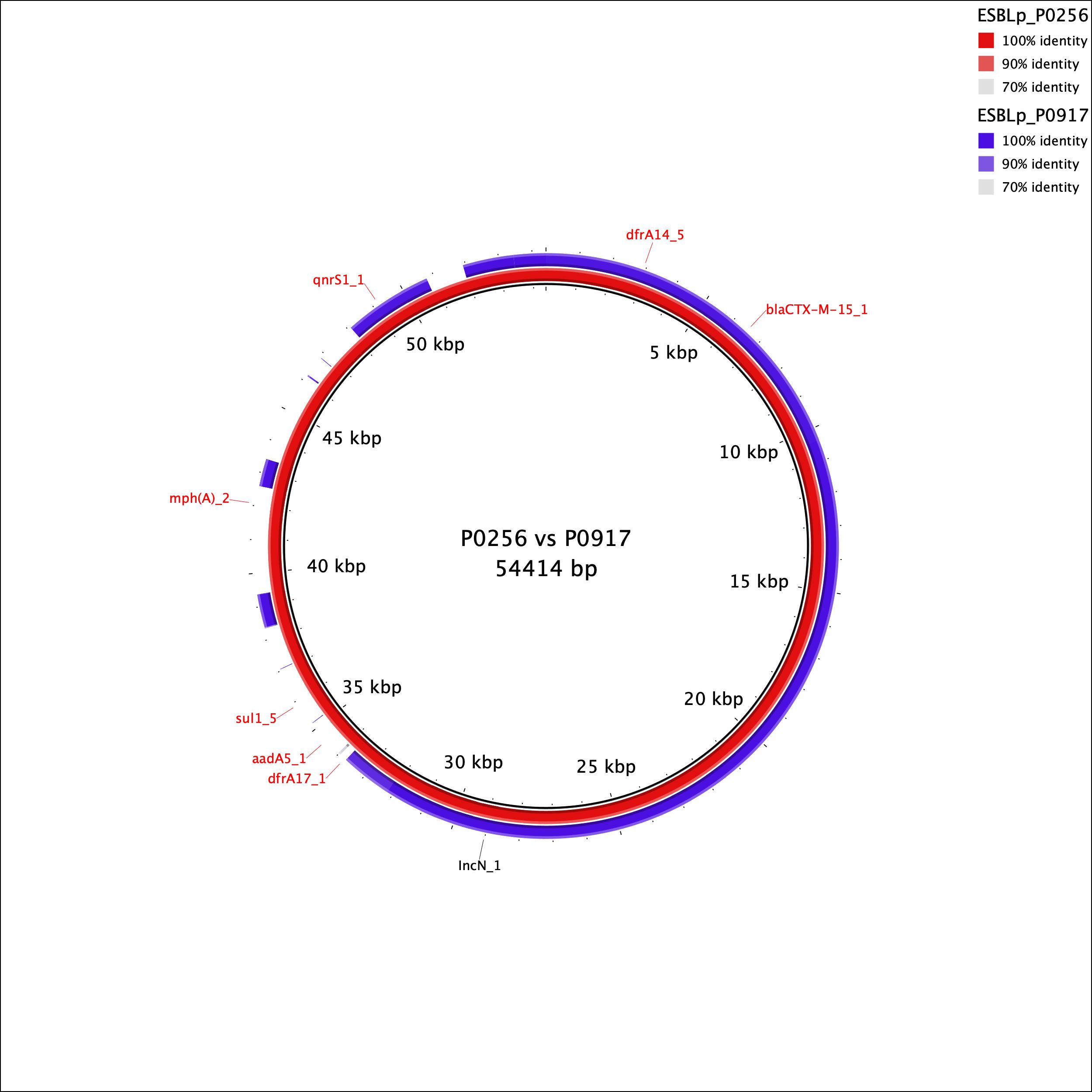

Supplement: Supplementary file 11 — Source Data [file 41467_2023_44285_MOESM11_ESM.zip › SourceDataFile/ESBLp_figures/Ecoli_ESBLp_BRIG_figures_allPacBio/P0256_ESBLp.fasta.jpg]

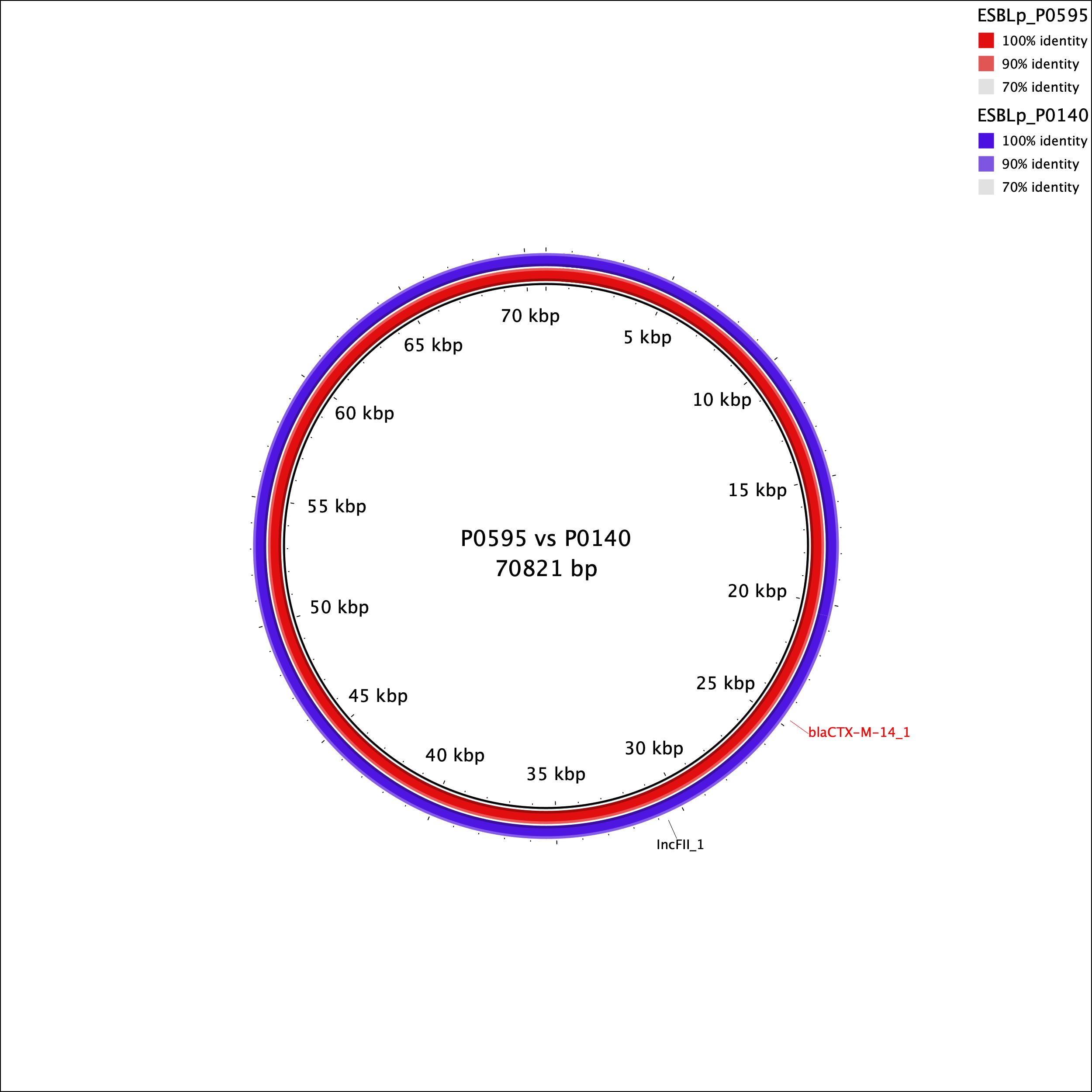

Supplement: Supplementary file 11 — Source Data [file 41467_2023_44285_MOESM11_ESM.zip › SourceDataFile/ESBLp_figures/Ecoli_ESBLp_BRIG_figures_allPacBio/P0595_ESBLp.fasta.jpg]

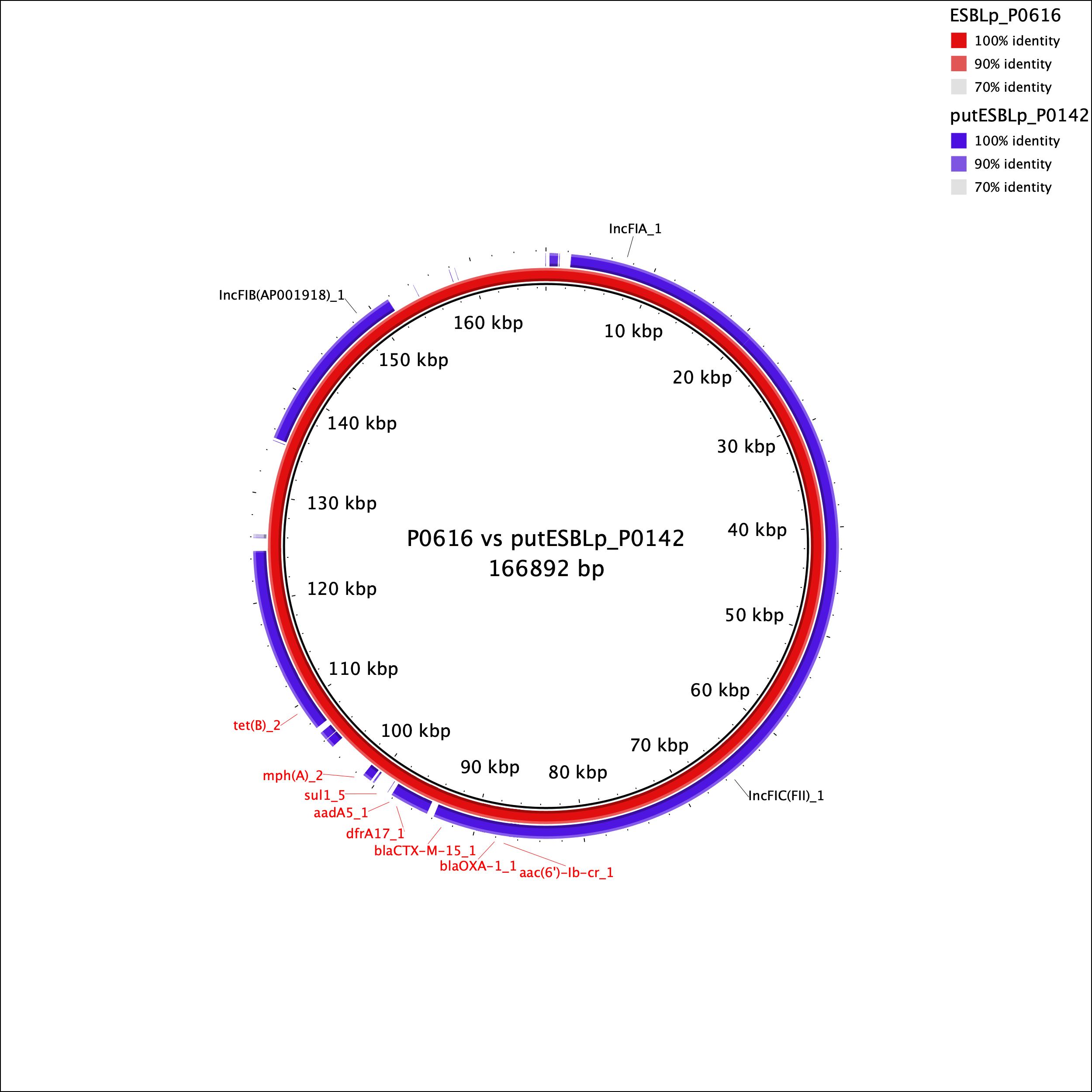

Supplement: Supplementary file 11 — Source Data [file 41467_2023_44285_MOESM11_ESM.zip › SourceDataFile/ESBLp_figures/Ecoli_ESBLp_BRIG_figures_allPacBio/P0616_ESBLp.fasta.jpg]

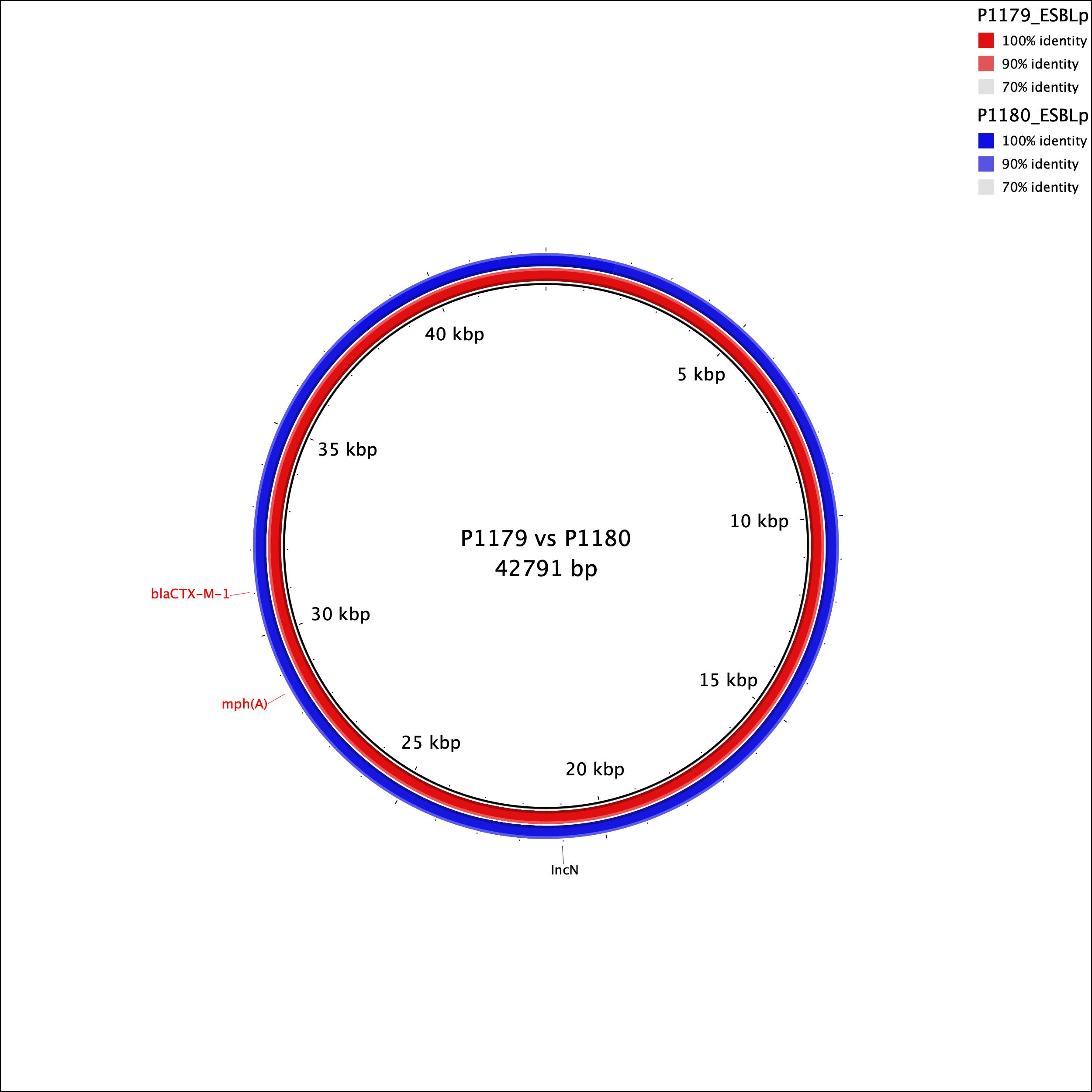

Supplement: Supplementary file 11 — Source Data [file 41467_2023_44285_MOESM11_ESM.zip › SourceDataFile/ESBLp_figures/Ecoli_ESBLp_BRIG_figures_allPacBio/P1179_ESBLp.fasta.jpg]

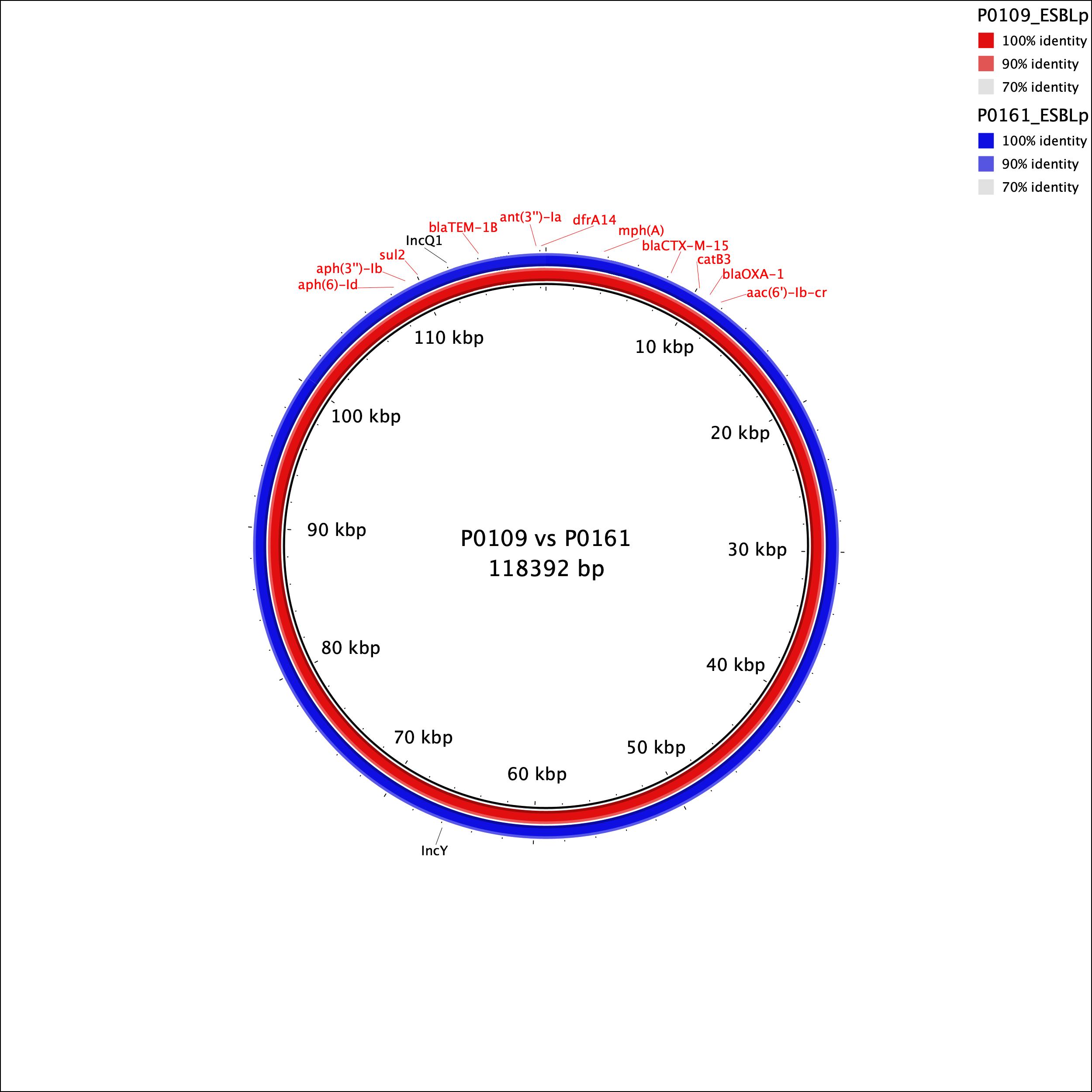

Supplement: Supplementary file 11 — Source Data [file 41467_2023_44285_MOESM11_ESM.zip › SourceDataFile/ESBLp_figures/Ecoli_ESBLp_BRIG_figures_allPacBio/P0109_ESBLp.fasta.jpg]

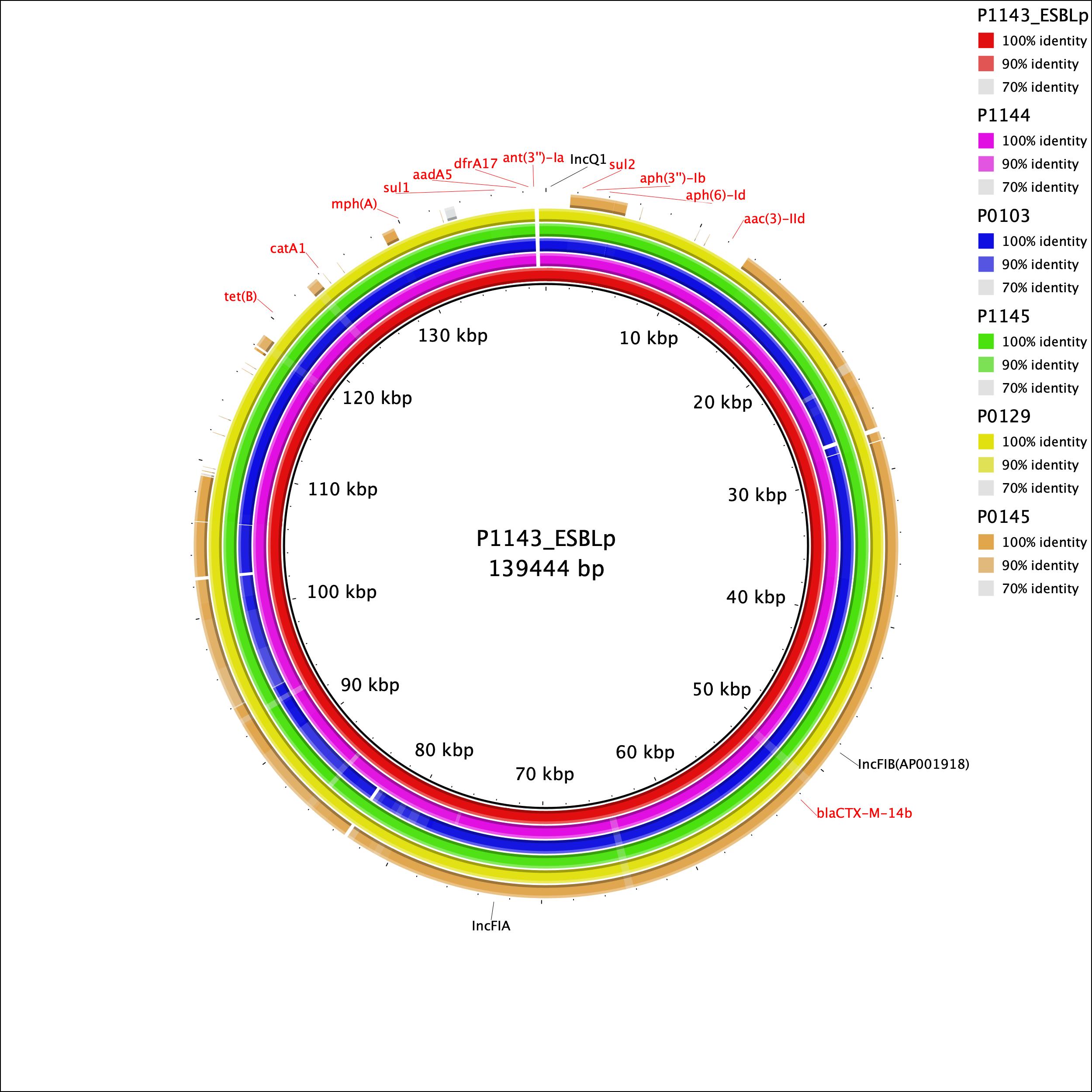

Supplement: Supplementary file 11 — Source Data [file 41467_2023_44285_MOESM11_ESM.zip › SourceDataFile/ESBLp_figures/Ecoli_BRIG_figures_refPacBio_othersIllumina/P1143_ESBLp.jpg]

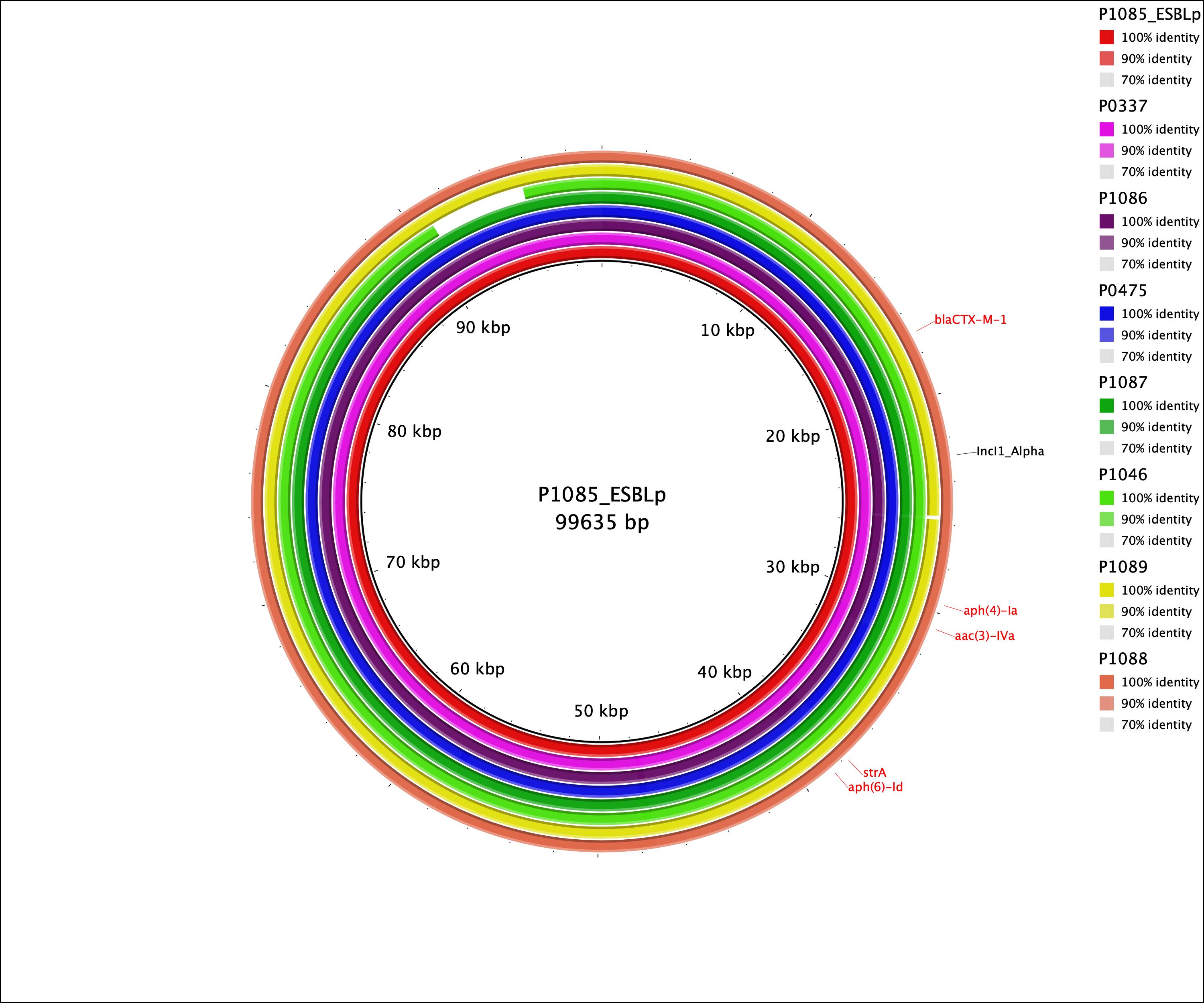

Supplement: Supplementary file 11 — Source Data [file 41467_2023_44285_MOESM11_ESM.zip › SourceDataFile/ESBLp_figures/Ecoli_BRIG_figures_refPacBio_othersIllumina/P1085_ESBLp.jpg]

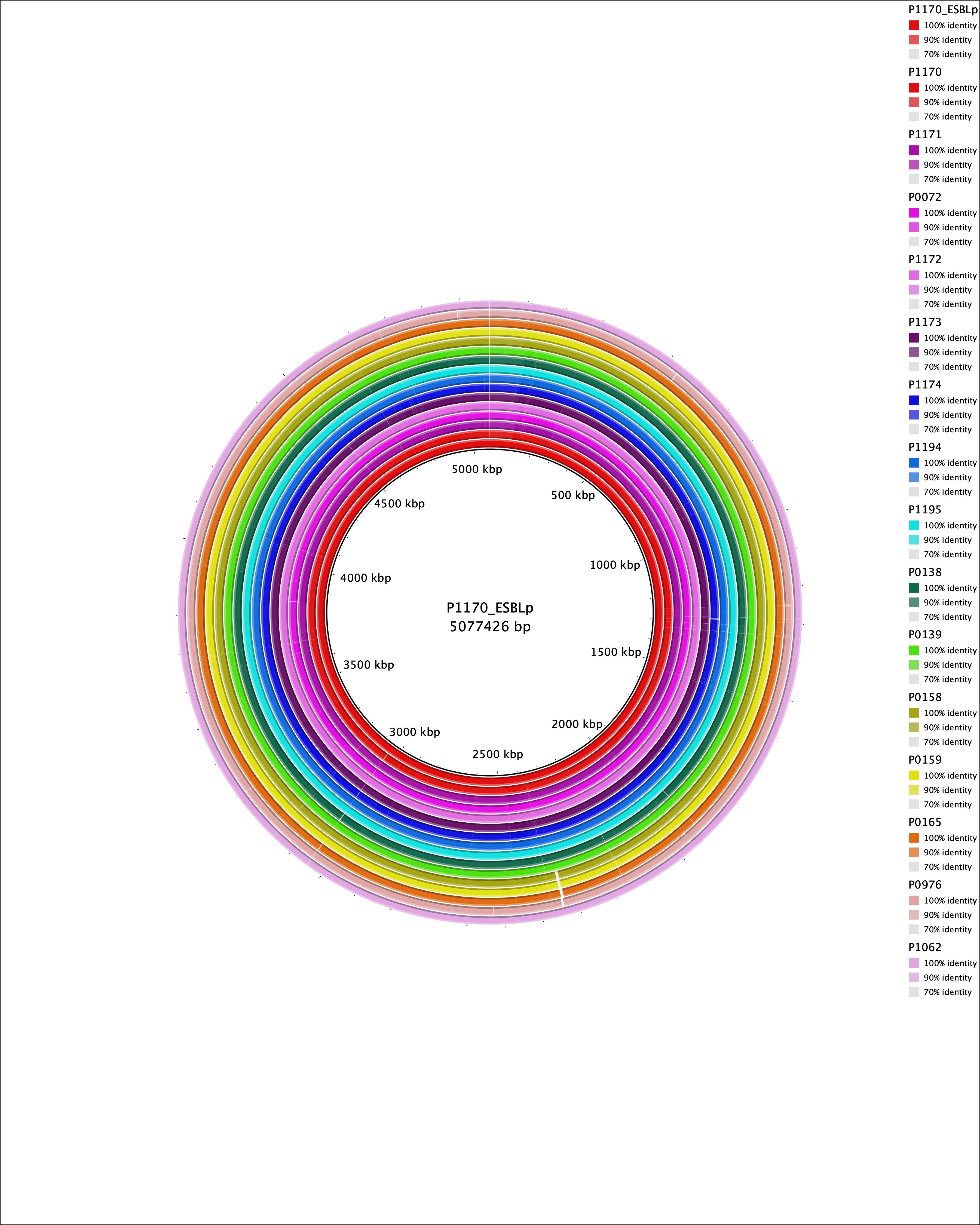

Supplement: Supplementary file 11 — Source Data [file 41467_2023_44285_MOESM11_ESM.zip › SourceDataFile/ESBLp_figures/Ecoli_BRIG_figures_refPacBio_othersIllumina/P1170_ESBLp.jpg]

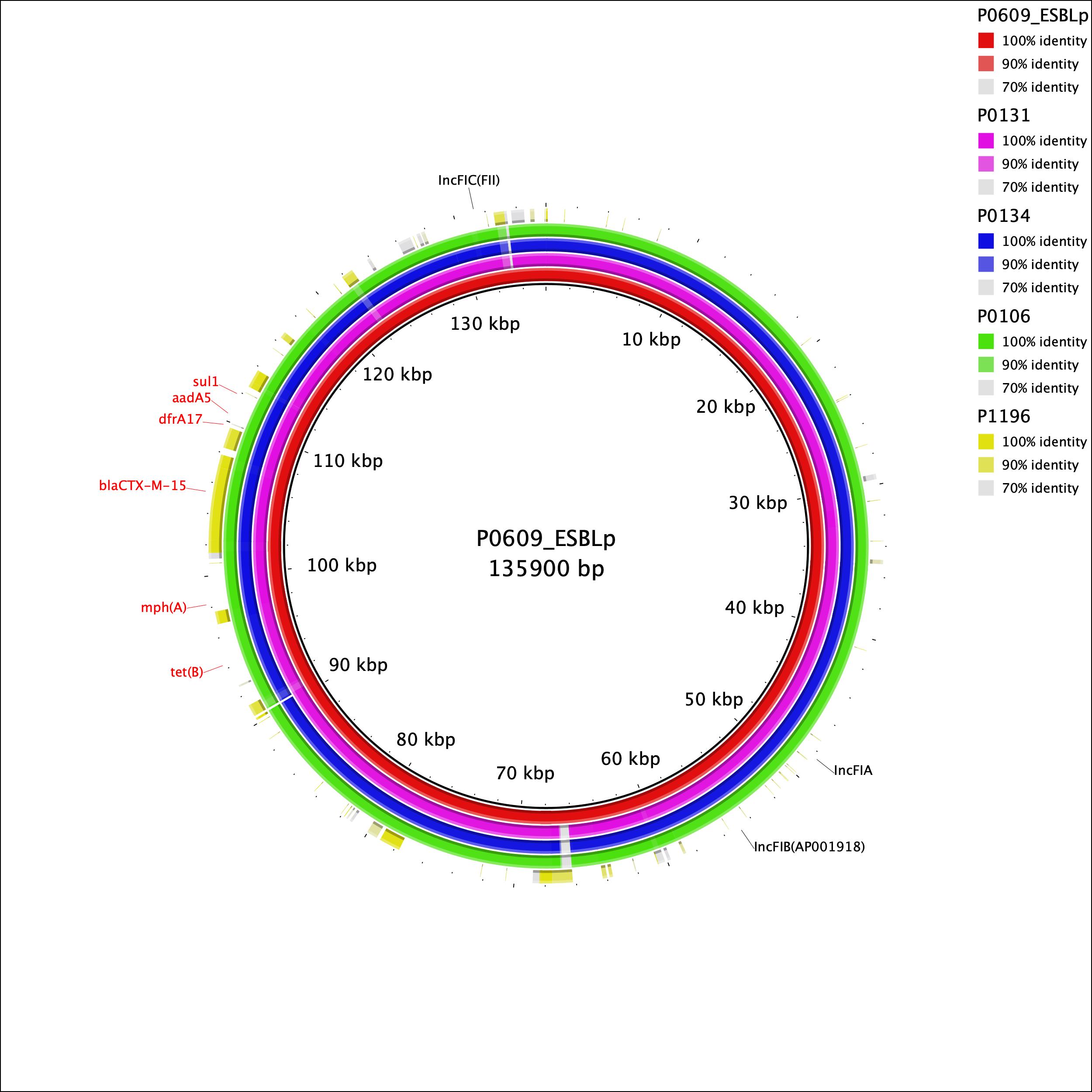

Supplement: Supplementary file 11 — Source Data [file 41467_2023_44285_MOESM11_ESM.zip › SourceDataFile/ESBLp_figures/Ecoli_BRIG_figures_refPacBio_othersIllumina/P0609_ESBLp.jpg]

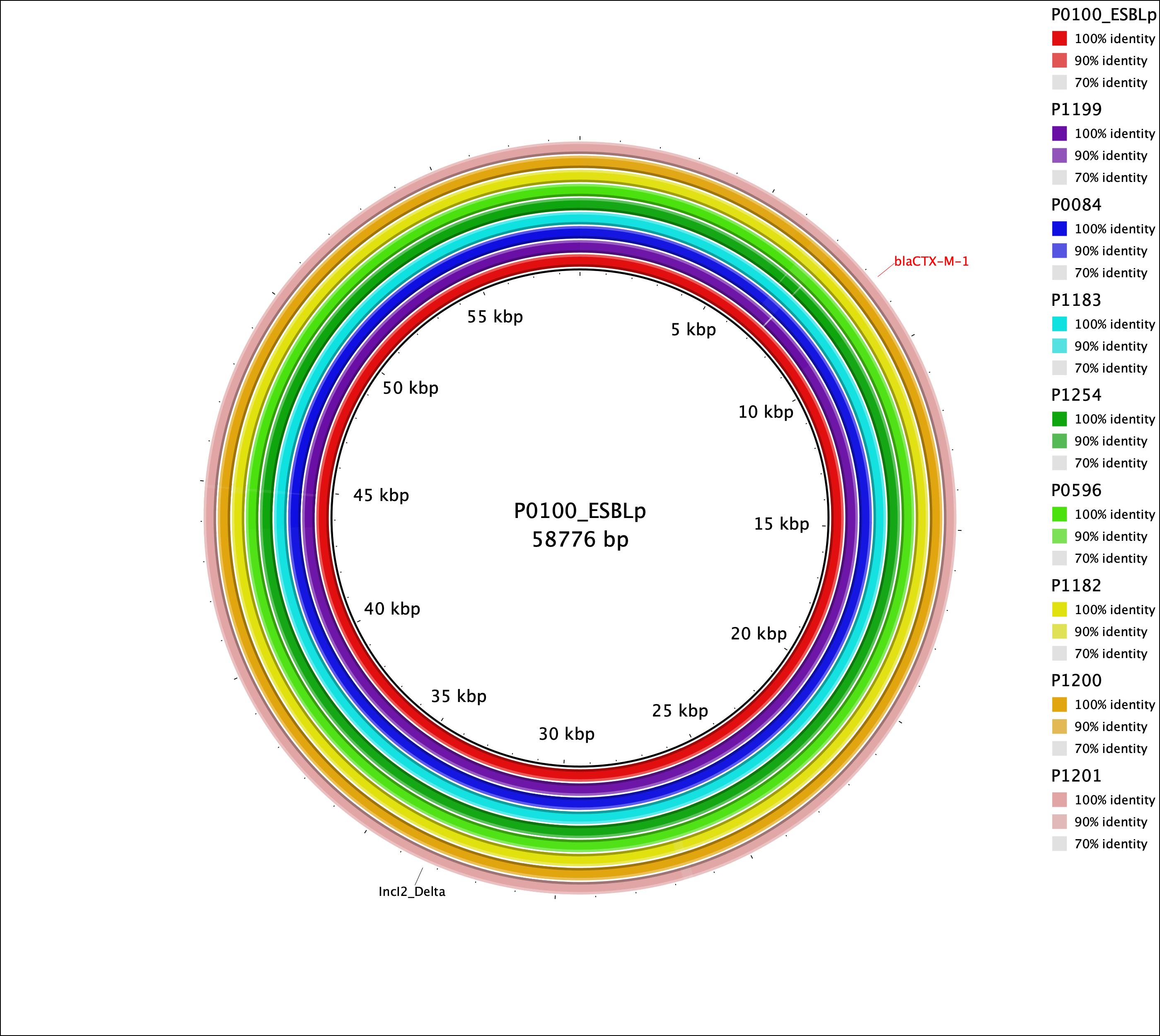

Supplement: Supplementary file 11 — Source Data [file 41467_2023_44285_MOESM11_ESM.zip › SourceDataFile/ESBLp_figures/Ecoli_BRIG_figures_refPacBio_othersIllumina/P0100_ESBLp.jpg]

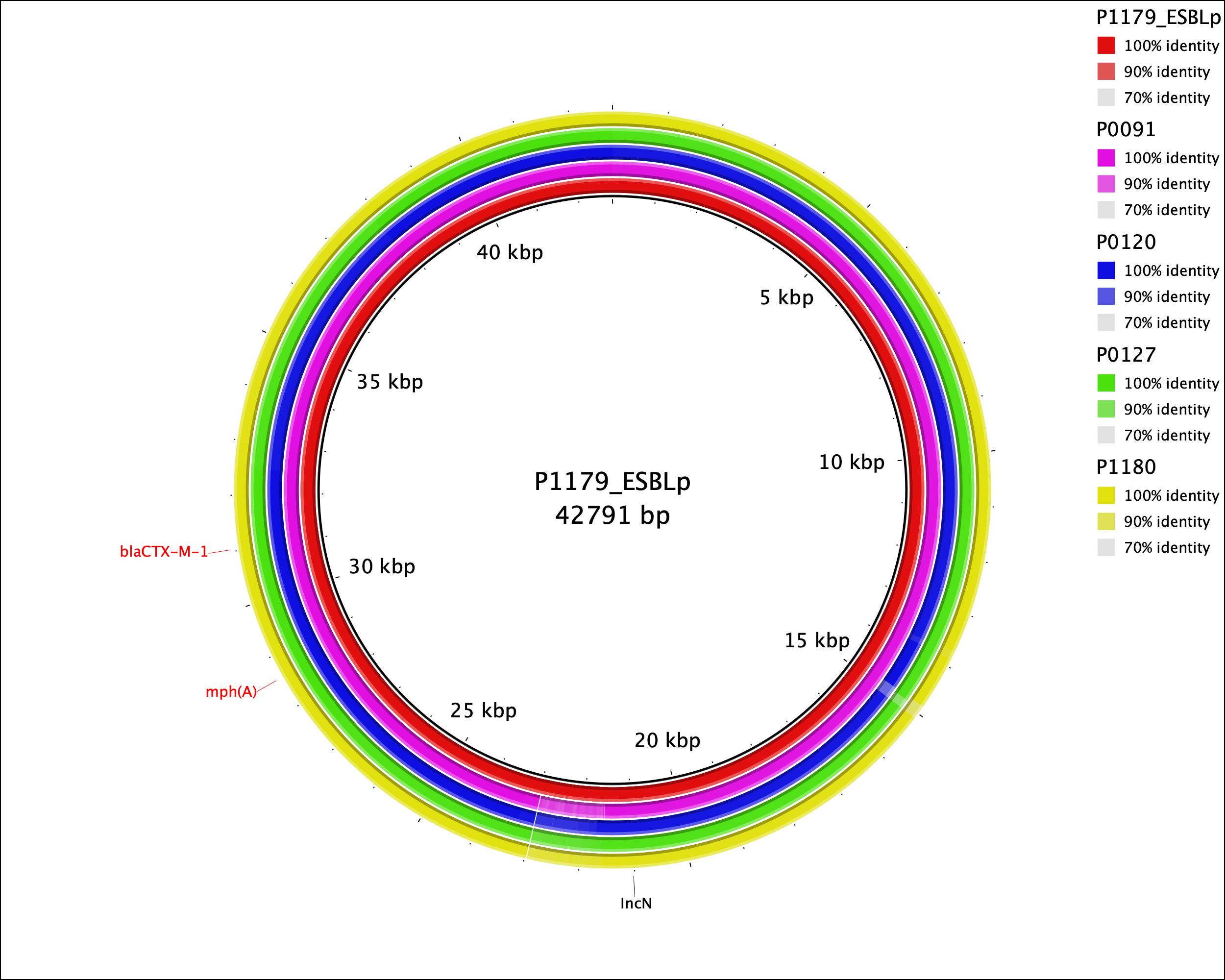

Supplement: Supplementary file 11 — Source Data [file 41467_2023_44285_MOESM11_ESM.zip › SourceDataFile/ESBLp_figures/Ecoli_BRIG_figures_refPacBio_othersIllumina/P1179_ESBLp.jpg]

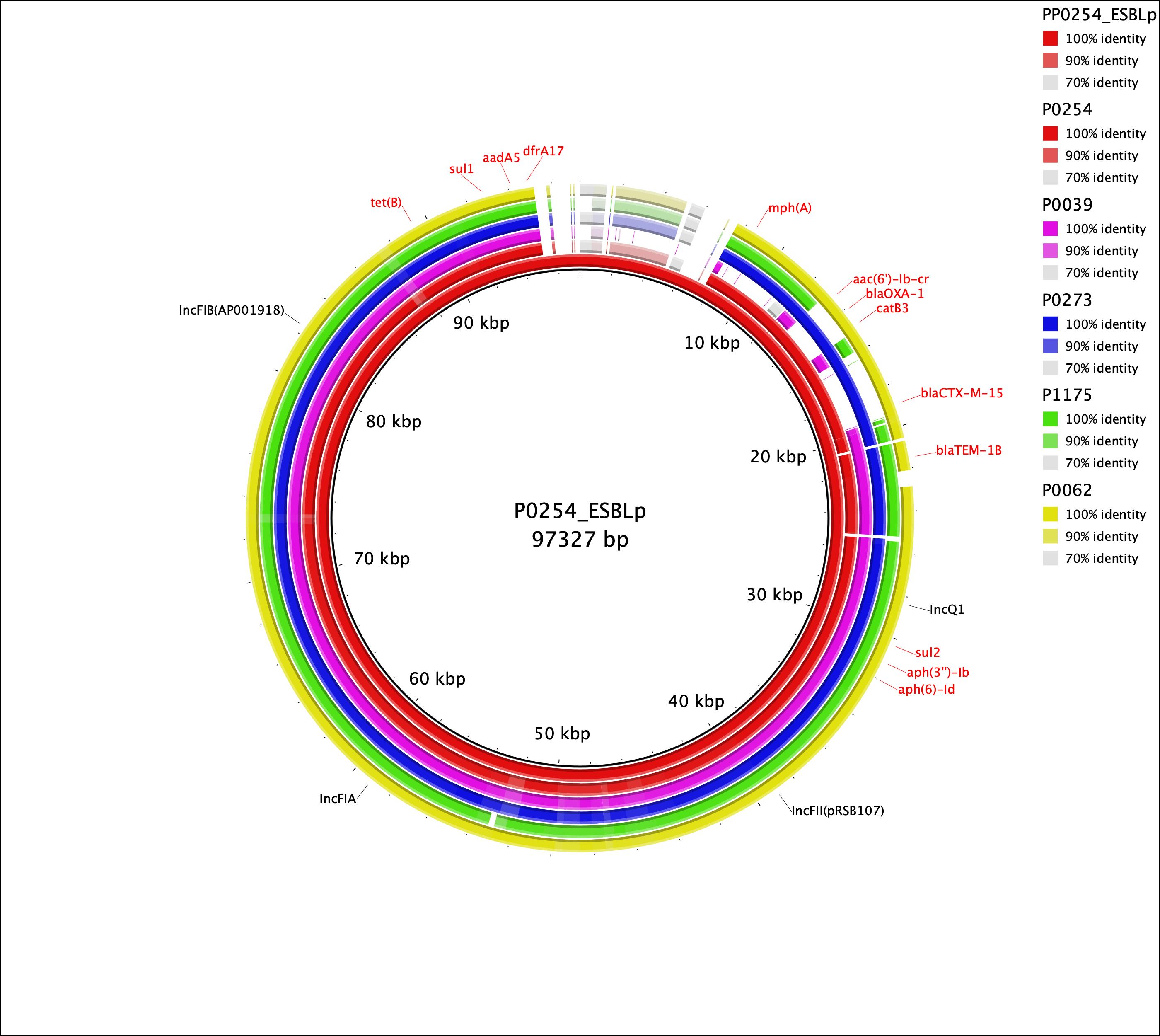

Supplement: Supplementary file 11 — Source Data [file 41467_2023_44285_MOESM11_ESM.zip › SourceDataFile/ESBLp_figures/Ecoli_BRIG_figures_refPacBio_othersIllumina/P0254_ESBLp.jpg]

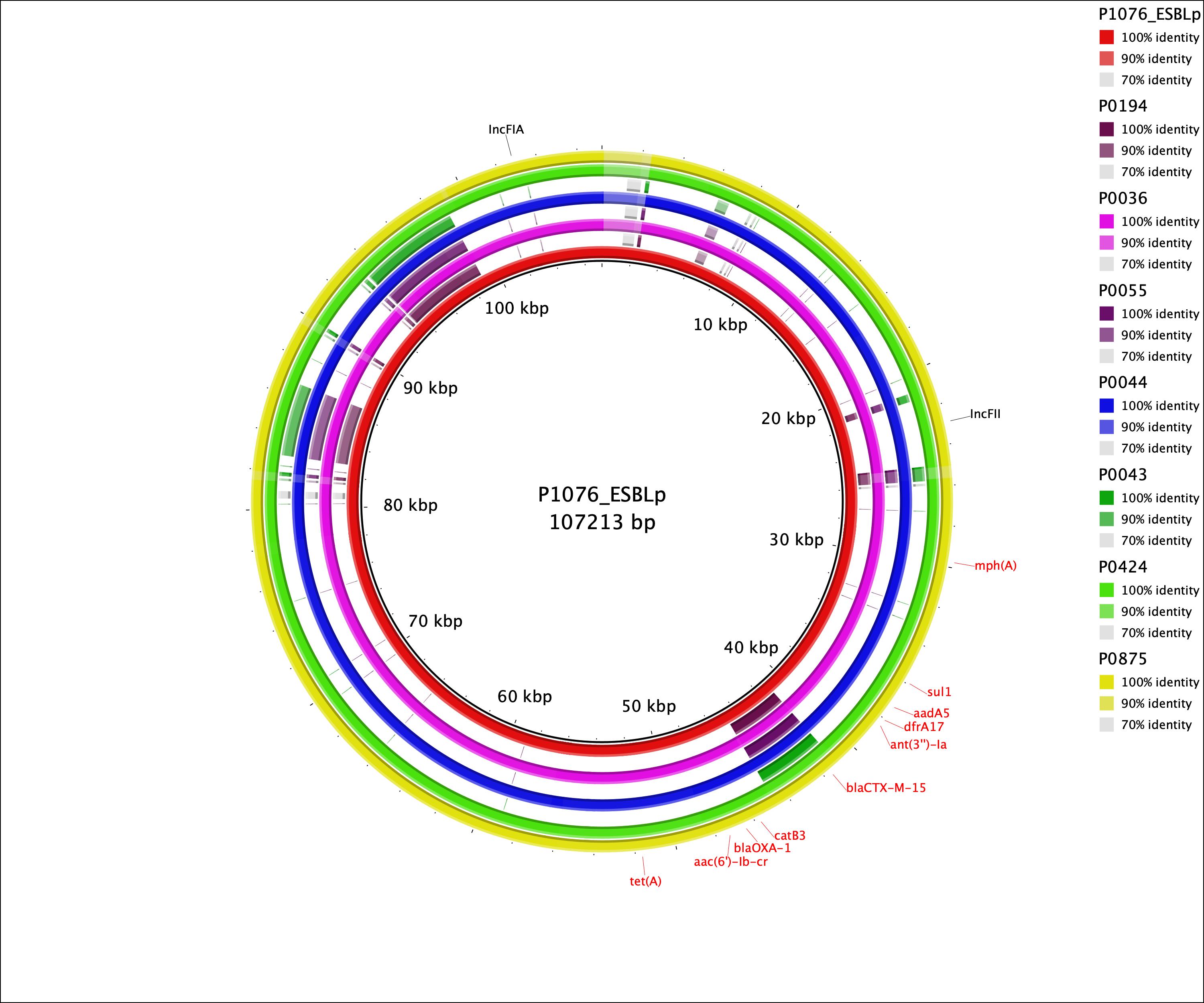

Supplement: Supplementary file 11 — Source Data [file 41467_2023_44285_MOESM11_ESM.zip › SourceDataFile/ESBLp_figures/Ecoli_BRIG_figures_refPacBio_othersIllumina/P1076_ESBLp.jpg]

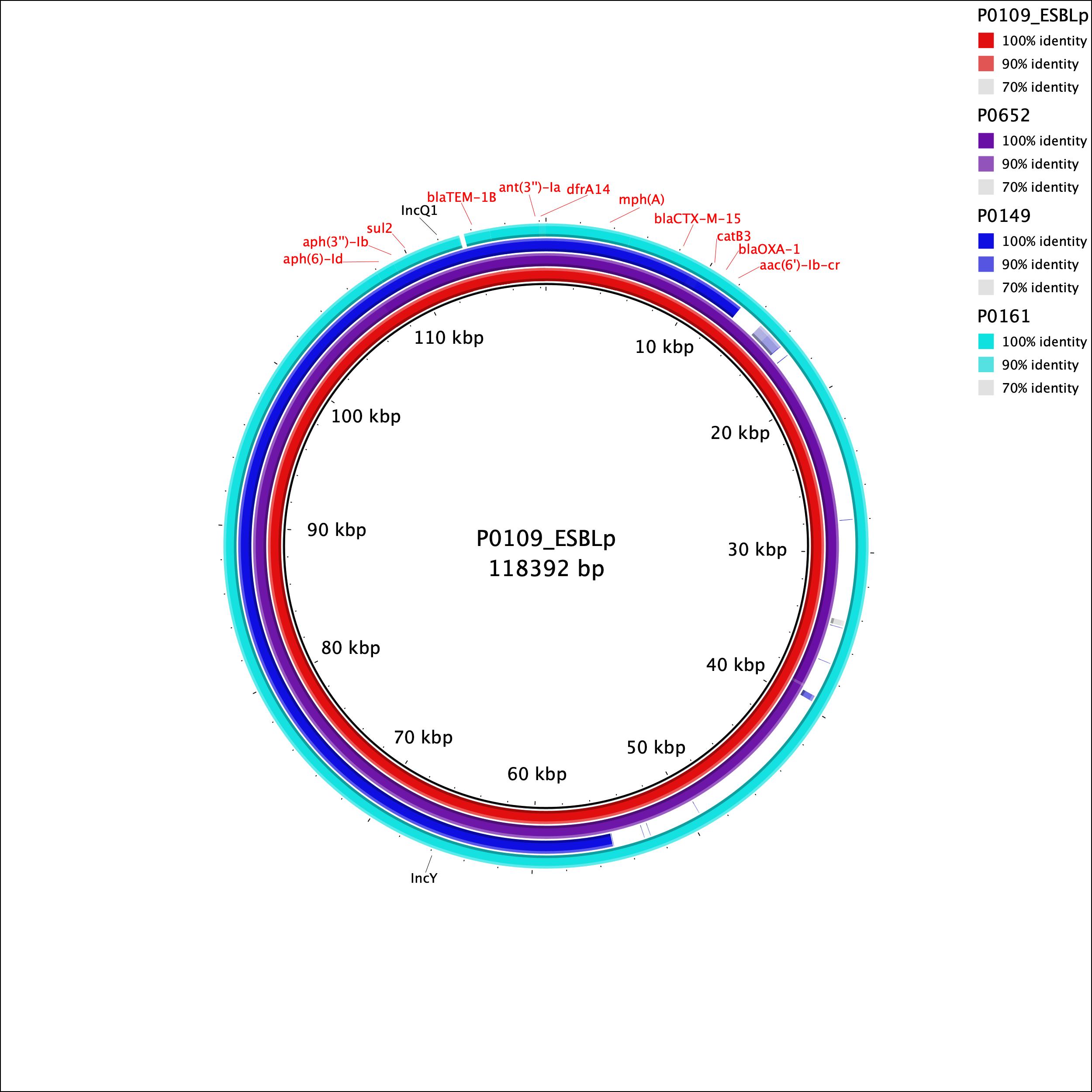

Supplement: Supplementary file 11 — Source Data [file 41467_2023_44285_MOESM11_ESM.zip › SourceDataFile/ESBLp_figures/Ecoli_BRIG_figures_refPacBio_othersIllumina/P0109_ESBLp.jpg]

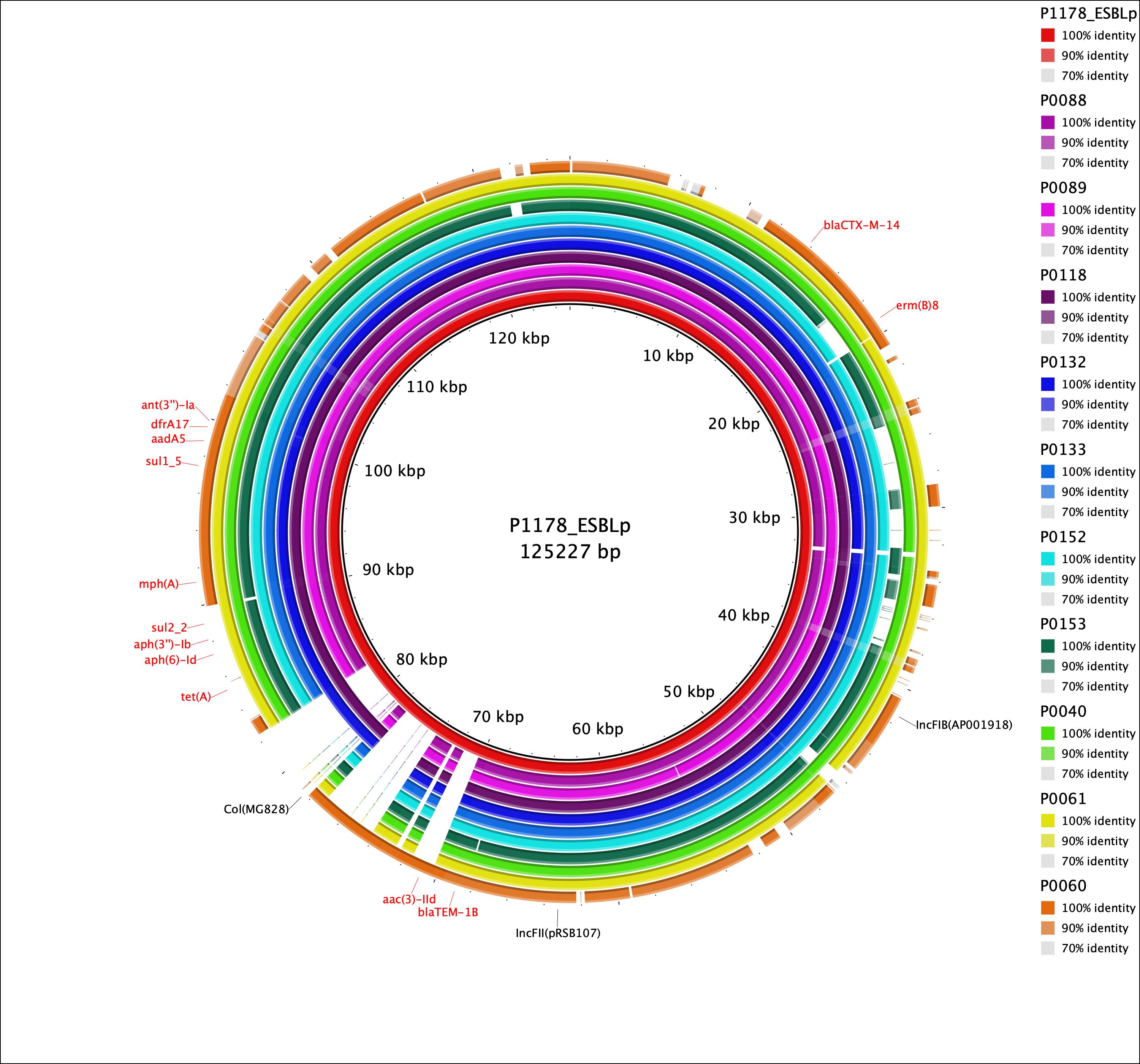

Supplement: Supplementary file 11 — Source Data [file 41467_2023_44285_MOESM11_ESM.zip › SourceDataFile/ESBLp_figures/Ecoli_BRIG_figures_refPacBio_othersIllumina/P1178_ESBLp.jpg]

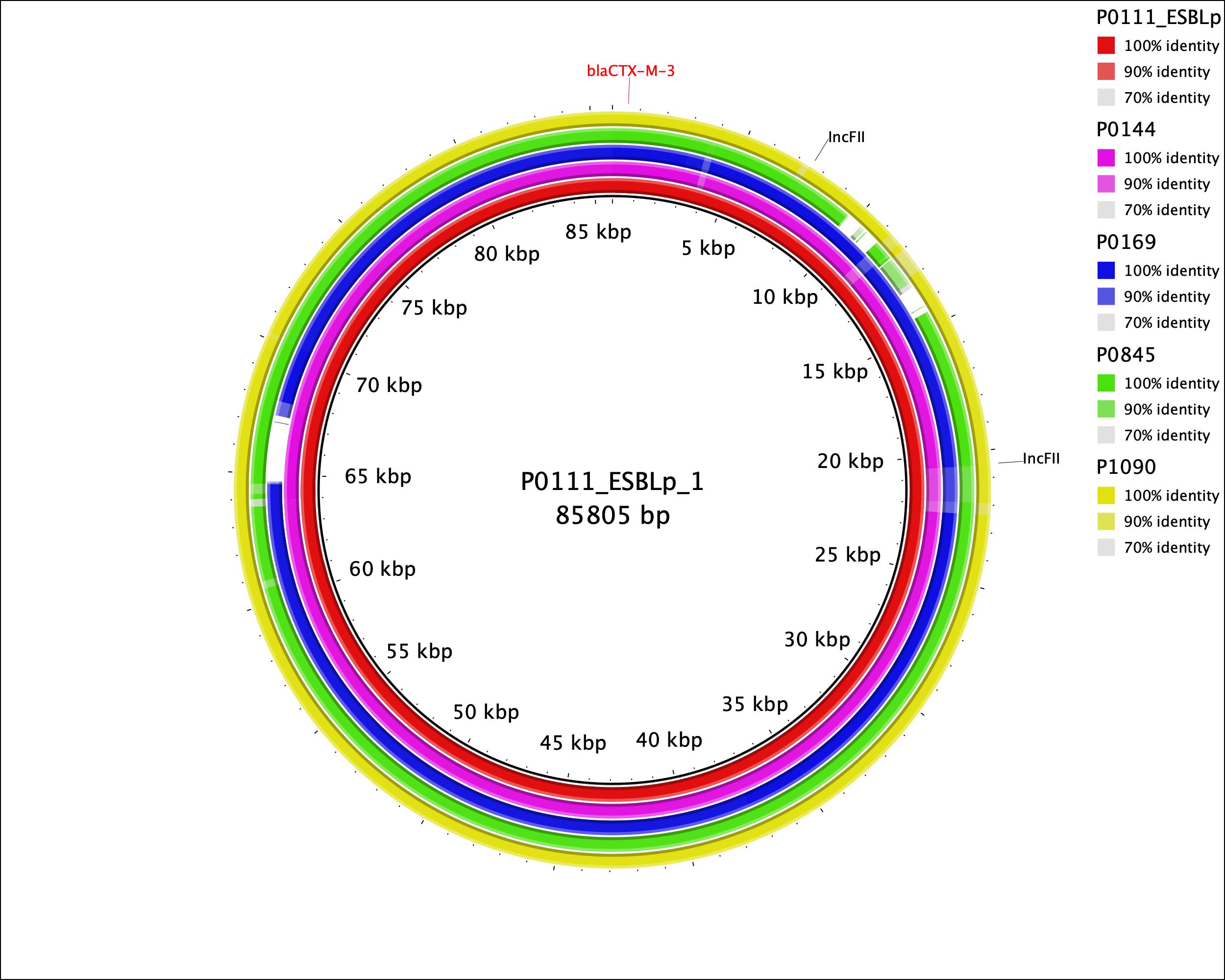

Supplement: Supplementary file 11 — Source Data [file 41467_2023_44285_MOESM11_ESM.zip › SourceDataFile/ESBLp_figures/Ecoli_BRIG_figures_refPacBio_othersIllumina/P0111_ESBLp1.jpg]

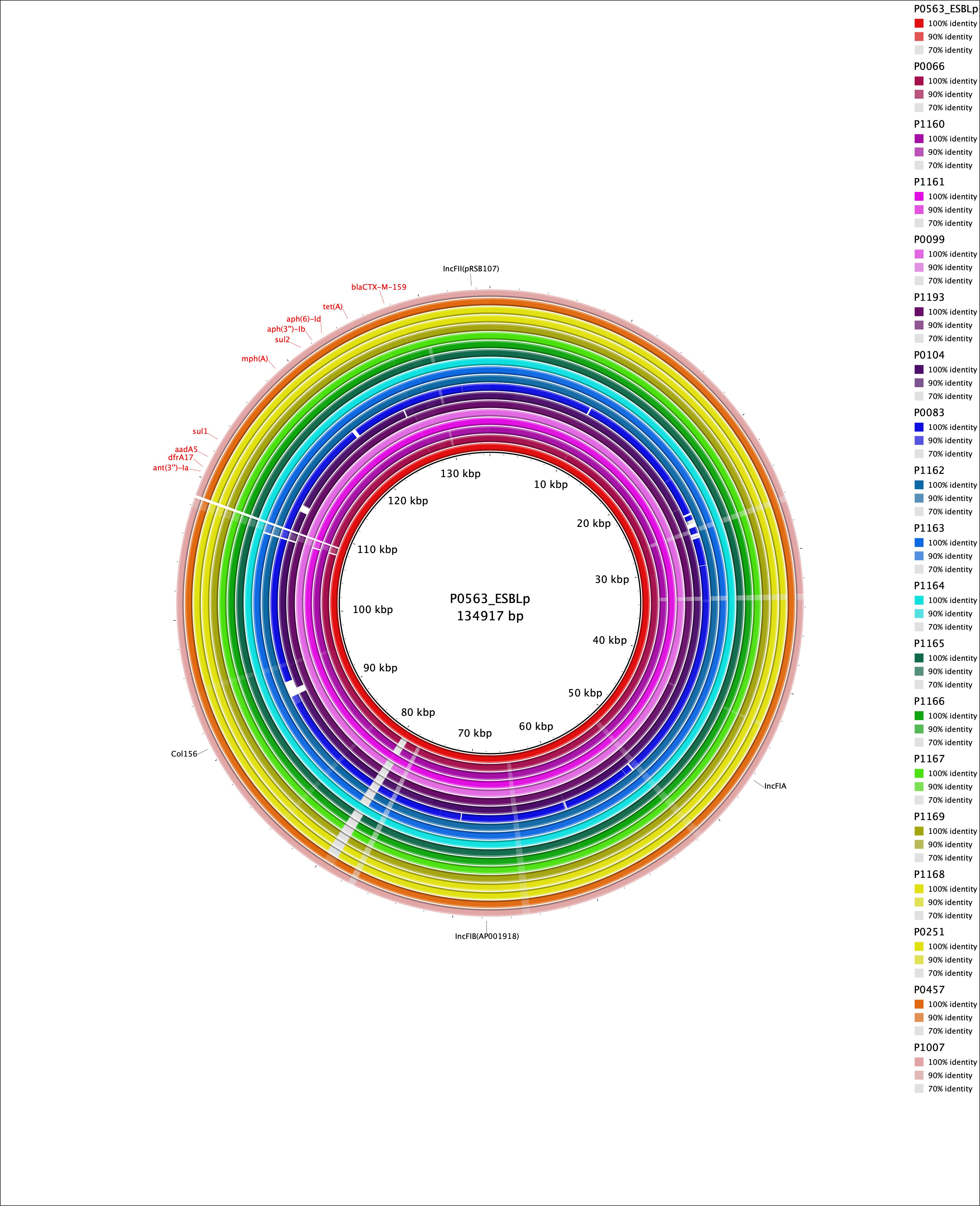

Supplement: Supplementary file 11 — Source Data [file 41467_2023_44285_MOESM11_ESM.zip › SourceDataFile/ESBLp_figures/Ecoli_BRIG_figures_refPacBio_othersIllumina/P0563_ESBLp.jpg]

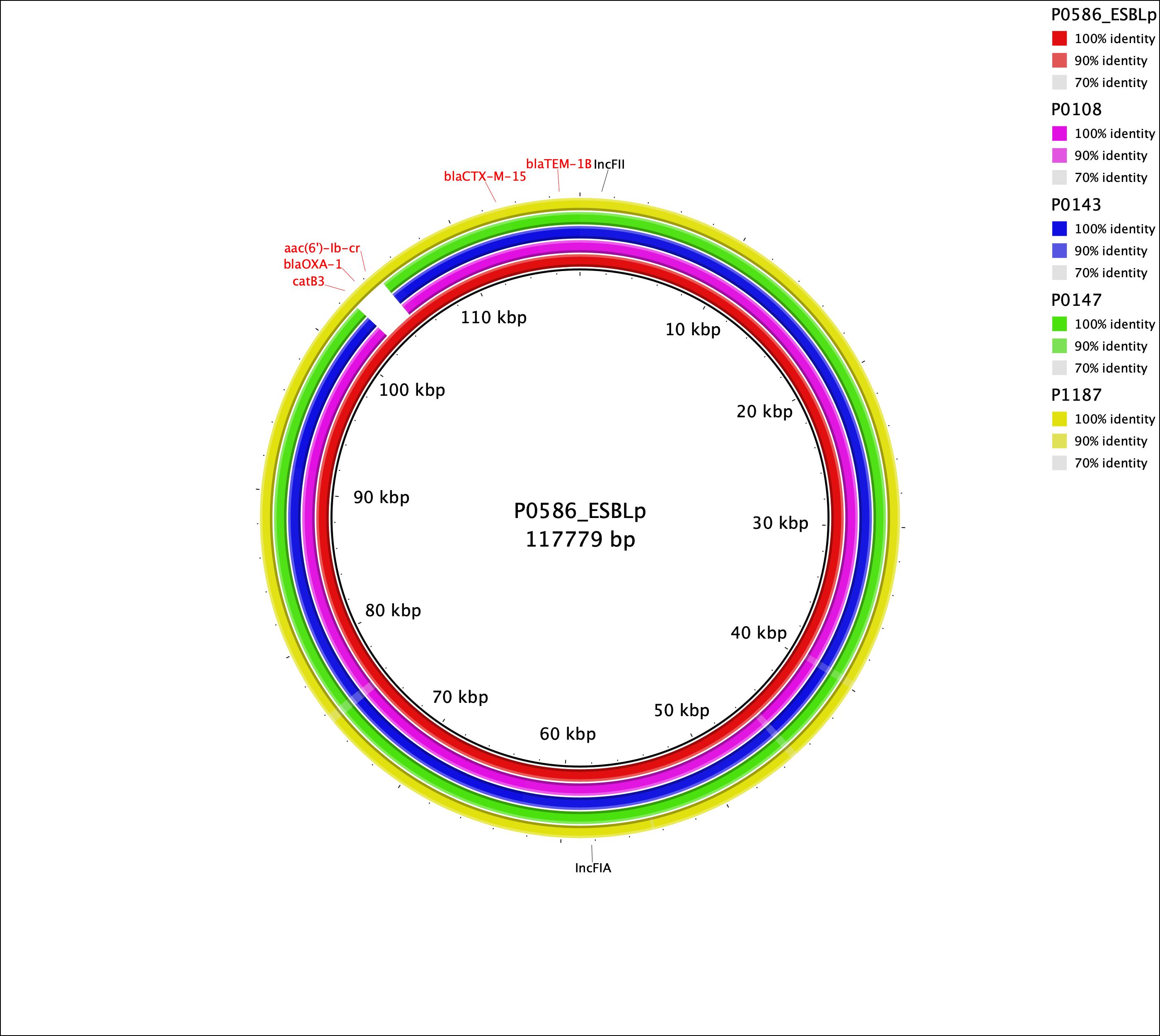

Supplement: Supplementary file 11 — Source Data [file 41467_2023_44285_MOESM11_ESM.zip › SourceDataFile/ESBLp_figures/Ecoli_BRIG_figures_refPacBio_othersIllumina/P0586_ESBLp.jpg]

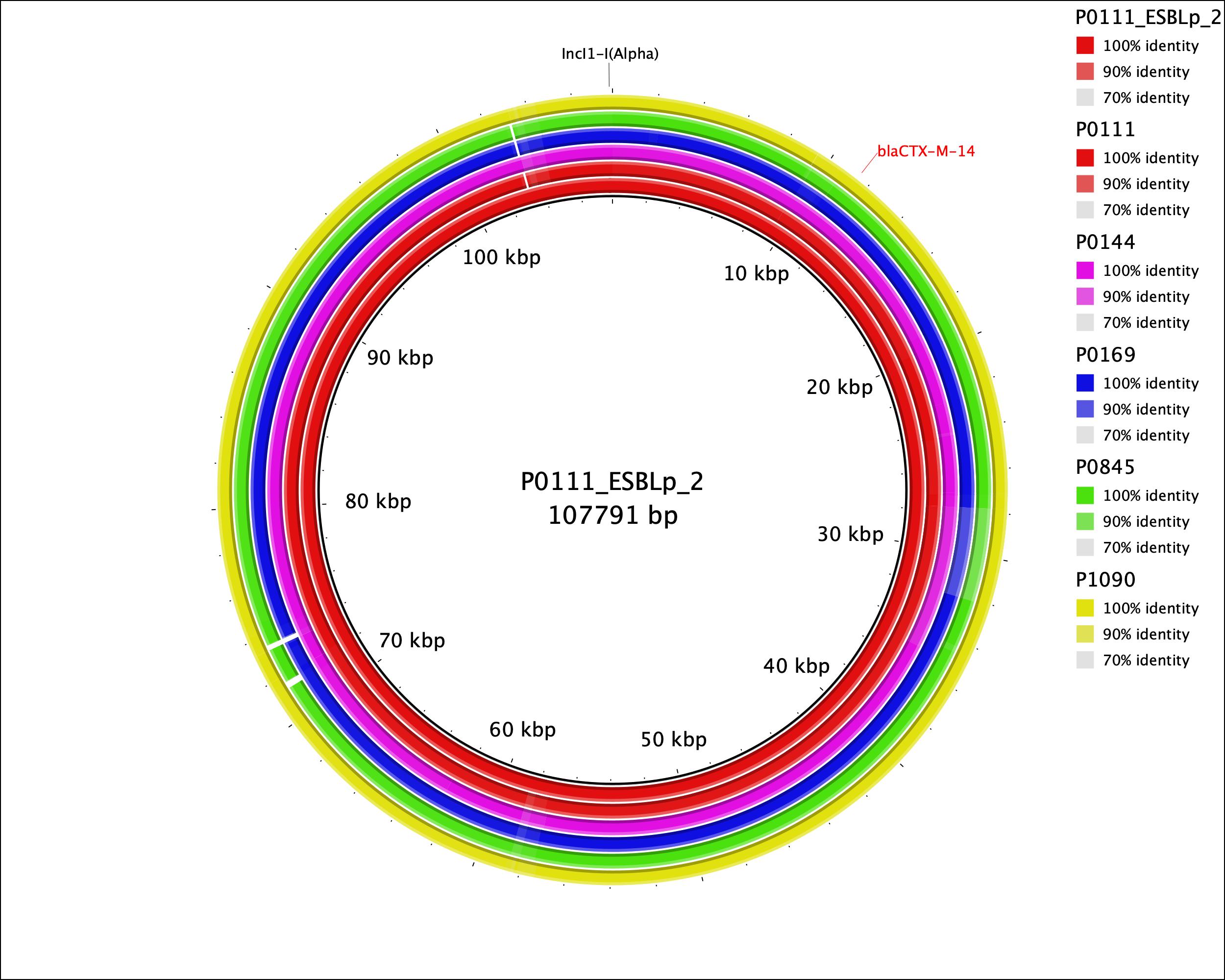

Supplement: Supplementary file 11 — Source Data [file 41467_2023_44285_MOESM11_ESM.zip › SourceDataFile/ESBLp_figures/Ecoli_BRIG_figures_refPacBio_othersIllumina/P0111_ESBLp2.jpg]
